# The first year of the COVID-19 pandemic in humanitarian settings: epidemiology, health service utilization, and health care seeking behavior in Bangui and surrounding areas, Central African Republic

**DOI:** 10.1186/s13031-023-00523-y

**Published:** 2023-05-20

**Authors:** Chiara Altare, Natalya Kostandova, Gbètoho Fortuné Gankpe, Patricia Nalimo, Abdoul Azizi Almoustapha Abaradine, IMPACT CAR Team, Sophie Bruneau, Caroline Antoine, Paul B. Spiegel

**Affiliations:** 1 Johns Hopkins Bloomberg School of Public Health, Baltimore, MD USA; 2 Johns Hopkins Center for Humanitarian Health, Baltimore, MD USA; 3 Action Contre la Faim, Bangui, Central African Republic; 4 IMPACT Initiative, Geneva, Switzerland; 5 Action Contre la Faim, Paris, France

**Keywords:** COVID-19, Humanitarian settings, Central African Republic, Health care utilization, Health care seeking behavior

## Abstract

**Background:**

Despite increasing evidence on COVID-19, few studies have been conducted in humanitarian settings and none have investigated the direct and indirect effects of the pandemic in the Central African Republic. We studied the COVID-19 epidemiology, health service utilization, and health care seeking behavior in the first year of the pandemic in Bangui and surrounding areas.

**Methods:**

This mixed-methods study encompasses four components: descriptive epidemiological analysis of reported COVID-19 cases data; interrupted time series analysis of health service utilization using routine health service data; qualitative analysis of health care workers’ perceptions of how health services were affected; and health care seeking behavior of community members with a household survey and focus group discussions.

**Results:**

The COVID-19 epidemiology in CAR aligns with that of most other countries with males representing most of the tested people and positive cases. Testing capacity was mainly concentrated in Bangui and skewed towards symptomatic cases, travelers, and certain professions. Test positivity was high, and many cases went undiagnosed. Decreases in outpatient department consultations, consultations for respiratory tract infections, and antenatal care were found in most study districts. Cumulative differences in districts ranged from − 46,000 outpatient department consultations in Begoua to + 7000 in Bangui 3; − 9337 respiratory tract infections consultations in Begoua to + 301 in Bangui 1; and from − 2895 antenatal care consultations in Bimbo to + 702 in Bangui 2. Consultations for suspected malaria showed mixed results while delivery of BCG vaccine doses increased. Fewer community members reported seeking care at the beginning of the pandemic compared to summer 2021, especially in urban areas. The fear of testing positive and complying with related restrictions were the main obstacles to seeking care.

**Conclusions:**

A large underestimation of infections and decreased health care utilization characterized the first year of the COVID-19 pandemic in Bangui and surrounding area. Improved decentralized testing capacity and enhanced efforts to maintain health service utilization will be crucial for future epidemics. A better understanding of health care access is needed, which will require strengthening the national health information system to ensure reliable and complete data. Further research on how public health measures interact with security constraints is needed.

**Supplementary Information:**

The online version contains supplementary material available at 10.1186/s13031-023-00523-y.

## Background

The COVID-19 pandemic declared by the World Health Organization (WHO) on March 20, 2020 has affected almost all countries and all aspects of societies. With more than 643 million cases and 6.6 million deaths reported by December 1, 2022 [1], the COVID-19 pandemic has challenged every health system in the world and led to a variety of governmental responses that aimed to contain the spread of the disease, maintain routine health services, and minimize societal disruptions while protecting their populations.

Since the identification of the novel SARS-CoV-2 virus in December 2019, extraordinary progress has occurred in understanding how the virus operates in the human body, transmission chains, risk factors for adverse outcomes up to the development of treatment strategies, and the production at scale of multiple effective vaccines. Effects on countries, economies, and communities varied across regions and over time as multiple waves of cases occurred at different times in different parts of the world.

Health systems in low- and middle-income countries (LMICs) and in humanitarian settings were considered at higher risk at the beginning of the pandemic due to their limited capacity to prepare and respond to epidemics and pandemics [2], pre-existing vulnerabilities ranging from already fragile, understaffed and underfunded health systems, limited available emergency care capacity, poor living conditions, limited access to water and sanitation, and potentially vulnerable population with precarious health status [3, 4]. Several modeling studies attempted to estimate the burden of infections in various LMICs and forced displacement settings in Africa and worldwide, depicting quite gloomy scenarios [5, 6]. Fortunately, these dire forecasts did not occur, although several waves occurred in all countries. Twenty million cases and 389,000 deaths were reported in LMICs hosting humanitarian settings by December 1, 2022 [7], with the majority of cases being asymptomatic and a low proportion of patients experiencing severe outcomes and death [8, 9]. Due to limited testing capacity, the actual number of cases and deaths is likely much higher, as confirmed by serological studies [10]. The underlying causes for the heterogeneity in disease spread across countries remain unclear. Several factors likely contributed to such different scenarios, including early introduction of response measures, previous experience with epidemics and emergencies, demographic factors, host genetics and cross-reactivity with other pathogens, and climate and environmental factors [9, 11].

Besides the direct effects of the spread of the SARS-CoV-2 virus, particularly concerning was the capacity of governments to maintain routine health services when resources and attention were diverted to a single disease. In previous large-scale epidemics in humanitarian contexts (e.g., Ebola in West Africa and cholera in Yemen) [12, 13], there was excess morbidity and mortality from communicable and non-communicable diseases. When the COVID-19 pandemic started, health systems attempted to cope by adapting service provision to minimize infections while ensuring continuity of services [14], with limited guidance and mixed results.

Despite the increasing evidence on COVID-19, fewer studies have been conducted in humanitarian settings and very few in the Central African Republic (CAR); a short report from Bossangoa [15], two opinion papers about response strategy and lessons learned from HIV [16, 17], and one commentary about vaccine outreach [18]. To our knowledge, no studies have been conducted on the direct and indirect effects of the COVID-19 pandemic in CAR. We aim to contribute to filling this gap by investigating the COVID-19 epidemiology, health service utilization, and health care seeking behavior in the first year of the pandemic in Bangui and surrounding areas. This is one of three case studies in humanitarian and fragile settings that were conducted within the USAID-funded collaboration among Johns Hopkins Center for Humanitarian Health, Action Contre la Faim (ACF), and IMPACT. The other two case studies focused on Mweso health zone, Democratic Republic of Congo, and Cox’s Bazar, Bangladesh.

## Methods

### Study setting

The study comprised five health districts in Bangui (Bangui 1, Bangui 2, Bangui 3) and surrounding areas (Bimbo and Begoua) with a total estimated population of 1.15 million, including mainly urban and peri-urban areas; most of the population is non-displaced. ACF has implemented nutrition programs in Bangui since 2006, which have been complemented over time with health, food security, and water, sanitation and hygiene activities in Bangui and surrounding areas.

### Study design and objectives

This mixed-methods study aims to investigate the epidemiology of COVID-19 and how health service utilization was affected during the first year of the pandemic. Specifically, the study encompasses four components: (a) descriptive epidemiological analysis of reported COVID-19 cases data; (b) quantitative assessment of changes in health service utilization using routine health service data; (c) qualitative analysis of health care workers’ (HCWs) perceptions of how health services were affected during COVID-19; and (d) health care seeking behavior of community members through a household survey and focus group discussions (FGDs).

### Data sources and study outcomes

The study used four data sources. More details about data and data collection methods can be found in Additional file 1: Sect. 1 Methods.

1. COVID-19 data

Two separate line lists were maintained in CAR, one by the *Institut Pasteur* (IP), which started COVID-19 testing in March 2020, and the second by the *Laboratoire National de Biologie Clinique de Santé Publique* (LNBCSP), where COVID-19 testing started in May 2020. Each line list includes confirmed cases of COVID-19 identified between March 14, 2020, and March 31, 2021, and individual-level information such as patient demographic information; clinical presentation; test-related information; test results; presence of comorbidities or other underlying conditions; and exposure risks. The complete list of variables, their definition, and level of completeness are included in Additional file 1: 1.1.1–1.1.2. The datasets are anonymized and use different identifiers, hindering cases matching. The IP line list includes both negative and positive results, while the LNCBSP only has confirmed cases. No information about clinical management and disease outcomes was recorded in any line lists. Due to a mistake identified in the age variable from the IP line list that could not be rectified, this variable was not used for the analysis. Population estimates for 2021 were obtained from the United Nations Population prospects [19].

2. Routine health data

Routine health data on consultations for selected health services originated from the national health information system. As the system is only partially digitalized, data were extracted from paper-based monthly health facility reports available at the district health offices within the study period (January 1, 2017, or 2018 depending on data availability, to March 31, 2021) using KoboCollect software. Quality checks were performed daily and consisted in checking data completeness, correcting errors, removing duplicates, and ensuring data consistency and accuracy. Poorly completed extraction forms were returned to the enumerator the following day, and mistakes were addressed before uploading the data. One of the authors (FG) coordinated data extraction with the support of two ACF colleagues and eight data collectors. Electronic versions of the data existed for 12, 10, and 13 months in Bangui 1, Bangui 2, and Begoua, respectively, and were obtained from the district health office. After assessing usability, electronic data were added to the manually extracted data. Data collection lasted three months, from April 1, 2021, to June 30, 2021. Outcomes of interest included new outpatient department (OPD) consultations; first antenatal care visit (ANC1); consultations for respiratory tract infections (RTI); consultations for malaria; Bacille Calmette-Guerin (BCG) vaccination. Definitions of outcome indicators used in the analysis are presented in Additional file 1: Table S2.

3. Qualitative interviews with HCWs

A purposive sample of HCWs from 10 health facilities supported by ACF in Bangui 2 and Begoua districts was selected to ensure the inclusion of various profiles, facility types and sizes, and rural and urban settings. Twenty-six HCWs were interviewed, including district health officers, medical doctors, nurses, midwives, pharmacists, and other support staff. Interviews occurred between June 30, 2021, and July 15, 2021, and were conducted either in French or Sango, depending on the respondent’s preference, and recorded upon the respondent’s consent. The topics addressed in the interviews included whether health services were affected during COVID-19 and how (e.g., delivery modality, human resources, closure of health facilities); Infection Prevention and Control (IPC) measures introduced at the health facility; perceptions and acceptance of the identified changes.

4. Community perspective

Primary quantitative data were collected via a two-stage random sampling household survey. In the first stage, villages were selected using probability proportional to the size, and households (second stage) were selected via random allocation of GPS points within the village. One respondent per household was interviewed, either the head of the household or another consenting adult. Sample size was calculated with a ± 5% margin of error and 95% confidence interval. Total sample size was 1045. Qualitative data were collected through 24 semi-structured FGDs with 192 participants, including community members, local chiefs, religious leaders, representatives of youth and women groups, and merchants, to ensure a variety of profiles were represented. Participants were purposively selected by IMPACT, in consultation with community leaders, local authorities and local actors. FGDs were stratified by age, sex, and displacement status. Both quantitative and qualitative components focused on health care seeking behavior at the beginning of the COVID-19 pandemic and how this changed over time (i.e., compared to the time of data collection). More details about primary data collection can be found in Additional file 1: Sect. 1.1.4.

### Analytical approach

1. COVID-19 Epidemiology

We performed a descriptive analysis to calculate the number of cumulative cases, testing and incidence rates, age and gender distribution, and clinical presentation. Quantitative variables were presented as mean ± confidence interval (CI), and categorical variables were expressed in frequencies. Multivariable logistic regression was used to identify risk factors associated with positive test results (using the IP database only). Analyses were conducted in Microsoft Excel and Stata version 14.

2. Changes in health service utilization

Health facilities were included in the analysis if they met all the following criteria: at least 12 months of data prior to the beginning of the COVID-19 period; at least three months of data during the COVID-19 period; at least six months of non-zero entries in the pre-COVID-19 period; not missing all last 12 months of data preceding the COVID-19 period. Outliers, defined as ± 3 standard deviations from the mean value by indicator and for each health facility, were removed in the pre-COVID-19 period.

To estimate how health service utilization has changed at the beginning and during the COVID-19 period, we fitted a generalized additive model to each health facility *i* using *mgcv* package (R software V.4.0.5) [20]:

$$\begin{aligned} y_{ij} & = NB\left( {y_{ij} |\mu_{ij} ,\theta_{i} } \right) \\ \log \left( {\mu_{ij} } \right) & = \alpha_{0i} + \alpha_{1i} S\left( {month} \right) + \beta_{1i} COVID\;period_{j} + \beta_{2i} COVID\;month_{j} \\ & \quad + \beta_{3i} S\left( {calemdar\;month_{j} ,cc,k = 3} \right) \\ \end{aligned}$$

where $y_{ij}$ is the number of consultations at health facility *i* in month *j*; NB denotes negative binomial distribution; $\alpha_{0i}$ is the facility-specific intercept; $\alpha_{1i}$ is the facility-specific coefficient for long-term trend; *month* is the variable for month of study, centered at beginning of COVID-19 period and smoothed; $COVID period$ is a variable taking value 0 in the pre-COVID-19 period (January 2018 – March 2020), and value of 1 in April 2020 onwards; *COVID month* is the month since beginning of COVID-19 period; $s\left( {calendar month_{j} , {\text{cc}}, k = 3} \right)$ is a cubic spline with three knots to capture seasonality (where applicable). For each of the five health districts, district-level estimates were obtained by pooling the facility-level estimates of *β*_1i_ and *β*_2i_ using inverse-variance meta-analysis approach, using *meta* package [21]. We report parameter estimates using the incidence rate ratio (IRR) and related 95% CI. For each outcome, we present the immediate change at the beginning of the COVID-19 period, and the trend change for the COVID-19 period. Classification of heterogeneity is also reported (details in Additional file 1: Sect. 1.2.1). We also calculated two measures of the difference with expected values: (1) the cumulative difference between observed and expected number of consultations (by type) over the study period; and (2) the average monthly percent change in consultations for each month of the COVID-19 period at each facility (details in Additional file 1: Sect. 1.2.2).

3. Health care workers’ perceptions

Framework analysis was used to explore qualitative data. A matrix output with cases as rows and codes as columns was developed to summarize data and facilitate comparisons between respondents and topics [22].

4. Health care seeking behavior

Qualitative data was analyzed using a saturation matrix. This process involves listing unique discussion points raised during all FGDs and counting the mentions to identify the most common opinions expressed. A weighted analysis of survey responses was conducted, disaggregated by age, sex, residence, and displacement status of respondents. Descriptive statistics (frequencies, means, proportions) were calculated; multivariable logistic regression investigated factors associated with seeking care (age, sex, displacement status, residence, setting, religion, education, profession). Analysis was conducted in R software with the “hypegrammR” [23], “koboquest” [24], and “surveyweights” [25] packages.

## Results

1. COVID-19 epidemiology

**Fig. 1:**
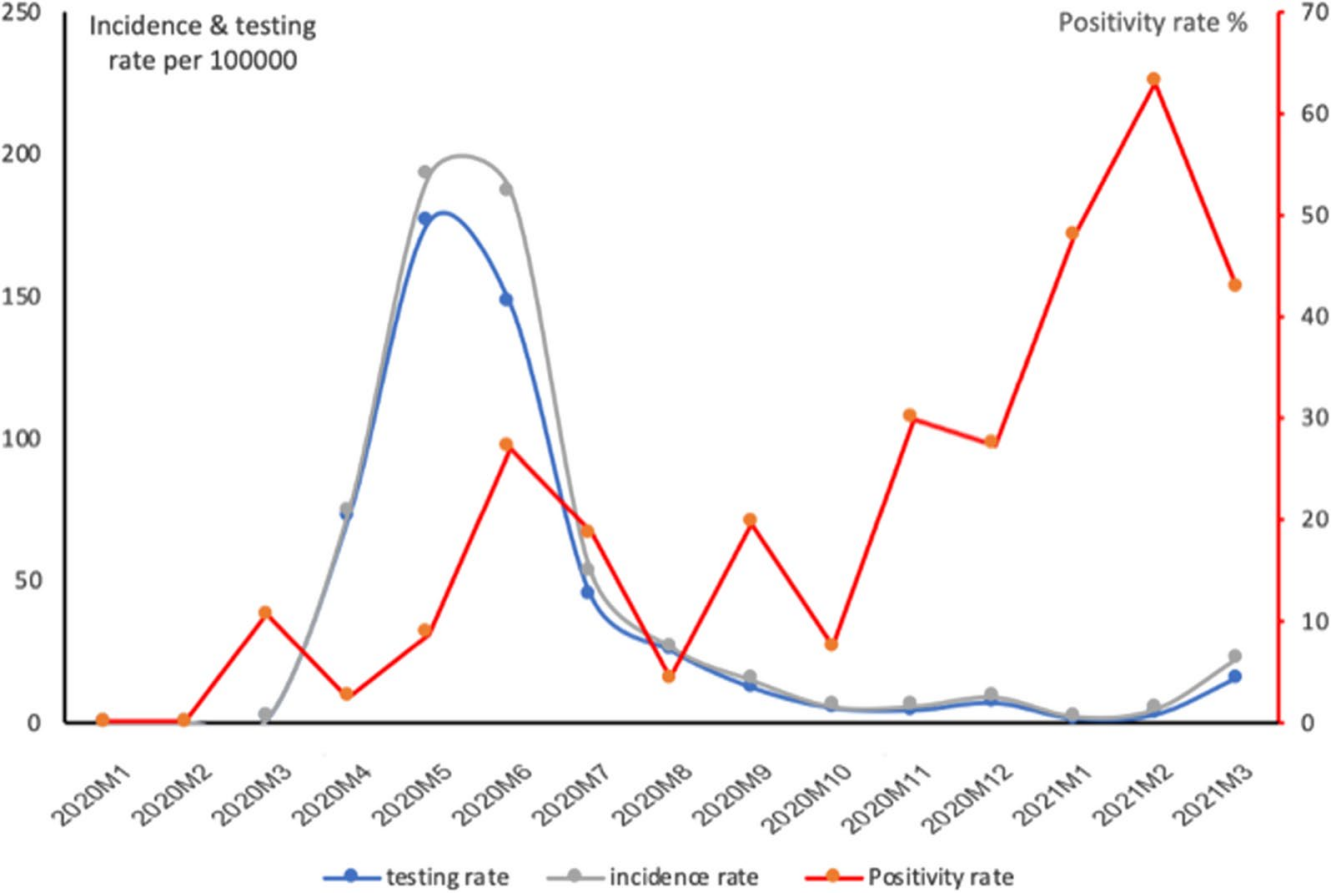
Trend of testing, incidence, and positivity rate per 100,000 population, March 2020 to April 2021, Central African Republic. *Note*: Testing rate only reflects tests conducted at the Institut Pasteur as the total number of conducted tests at the LNBCSP was unavailable

Table 1 outlines key measures related to cases, incidence rate, and testing capacity, and Fig. 1 shows their evolution over time. Additional results, including descriptive statistics of the cases, tests and factors associated with testing positive, are presented in Additional file 1: Tables S5 to S7. Males comprised most of the tested people and most of the COVID-19 cases. Testing capacity was mainly concentrated in Bangui; it increased for 4–5 months during the first wave, then decreased, corresponding to an increasing test positivity rate. Symptomatic people and travelers were more likely to test positive, and the incidence rate was higher among the elderly. Clinical presentation of cases aligns with global epidemiology. The two combined datasets have an epidemiological curve consistent with WHO-reported data (usually based on official data aggregated at country level), possibly implying that datasets have limited overlap (Additional file 1: Fig. S1).

2. Health service utilization

**Table 1:** Incidence and testing rates for the entire population and by age groups, CAR, March 14, 2020, to March 31, 2021

	IP	LNBCSP			
	Total population	Total population	0–19 years	20–59 years	60 + years
Number of tests conducted	25,188	3340	–	–	–
Positive results (Cases (n))	3992	3339	243	2734	191
Population (in thousands)	4920	4920	2727	1970	222
Incidence Rate (per 100,000 per year)	81.1	68.9	8.91	138.78	86.04
95% CI	81.13–81.15	66.58–71.22	7.79–10.03	133.58–143.98	78.83–98.23
Testing rate (per 100,000 per year)	511.9	NA	NA	NA	NA
Positivity rate	15.9%	NA	NA	NA	NA

Source of population estimate [19]

**Fig. 2:**
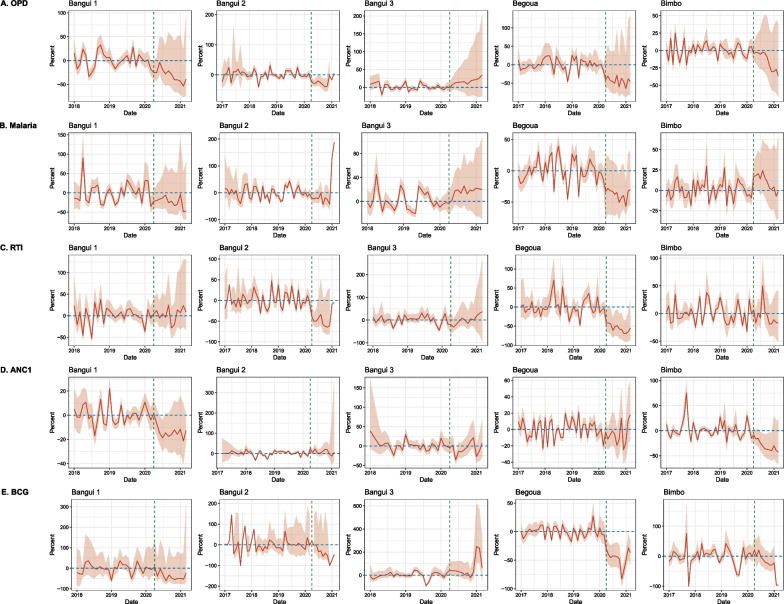
Percent deviation from expected values by indicator and health district, 2017–2021, Central African Republic

Table 2 reports the results of the interrupted time series analysis, cumulative difference, and average monthly % difference for each indicator and health district. Figure 2 shows the percent deviation from expected values by indicator and health district. Forest plots for immediate change and change in slope by indicator and district are presented in Additional file 1: Sect. 2.2.

**Table 2:** Interrupted time series results for outcome indicators, by district, CAR, 2017–2021: Incidence rate ratio for immediate change and change in slope; classification of heterogeneity; cumulative difference and average monthly change

Health district	N facilities	Measure	IRR [95%CI]	Heterogeneity	Cumulative difference [CI]	Average monthly % change [CI]
*OPD*						
Bangui 1	7	Immediate effect	0.884 [0.628; 1.244]	Moderate	− 38,121 [− 61,185; − 15,900]	− 25 [− 37; − 5]
		Change in slope	1 [0.919; 1.088]	High		
Bangui 2	10	Immediate effect	0.795 [0.615; 1.028]	Low	− 17,722 [− 23,938; − 11,348]	− 13 [− 33; 8593]
		Change in slope	0.985 [0.956; 1.016]	Low		
Bangui 3	6	Immediate effect	0.989 [0.809; 1.208]	Moderate	7250 [16,67; 12,435]	21 [6; 42]
		Change in slope	1.017 [0.97; 1.067]	Low		
Begoua	17	Immediate effect	0.983 [0.684; 1.414]	High	− 46,501 [− 82,113; − 19,922]	− 34 [− 53; − 7]
		Change in slope	1.02 [0.968; 1.074]	Low		
Bimbo	15	Immediate effect	1.052 [0.937; 1.182]	Low	− 14,390 [− 27,341; − 2950]	− 12 [− 22; 1]
		Change in slope	0.98 [0.952; 1.009]	Low		
*Malaria*						
Bangui 1	7	Immediate effect	0.734 [0.285; 1.892]	High	− 8594 [− 13,812; − 3283]	− 19 [− 31; 4]
		Change in slope	1.041 [0.941; 1.152]	High		
Bangui 2	9	Immediate effect	0.929 [0.67; 1.288]	Low	− 6593 [− 10,068; − 3299]	35 [− 20; 4080]
		Change in slope	0.979 [0.911; 1.051]	Low		
Bangui 3	8	Immediate effect	1.091 [0.885; 1.344]	Low	4345 [1153; 6998]	18 [5; 31]
		Change in slope	1.02 [0.955; 1.089]	Low		
Begoua	18	Immediate effect	1.116 [0.684; 1.82]	High	− 17,826 [− 24,192; − 11,621]	− 36 [− 46; − 24]
		Change in slope	0.973 [0.931; 1.016]	Low		
Bimbo	17	Immediate effect	1.261 [0.983; 1.617]	High	2894 [624; 4996]	12 [3; 23]
		Change in slope	0.997 [0.963; 1.032]	Low		
*RTI*						
Bangui 1	6	Immediate effect	0.984 [0.649; 1.494]	Low	301 [− 1394; 1688]	7 [− 6; 24]
		Change in slope	0.995 [0.905; 1.094]	Low		
Bangui 2	9	Immediate effect	0.639 [0.404; 1.011]	High	− 8496 [− 12,824; − 4931]	742 [− 56; 2535]
		Change in slope	1.002 [0.945; 1.063]	Low		
Bangui 3	5	Immediate effect	1.022 [0.55; 1.897]	Moderate	− 46 [− 1415; 1325]	6 [− 14; 33]
		Change in slope	1.031 [0.932; 1.141]	Low		
Begoua	13	Immediate effect	0.888 [0.496; 1.591]	High	− 9337 [− 14,858; − 5479]	− 52 [− 63; − 40]
		Change in slope	**0.972 [0.946; 0.999]**	Low		
Bimbo	17	Immediate effect	0.921 [0.72; 1.178]	Moderate	− 706 [− 2191; 869]	− 2 [− 11; 8]
		Change in slope	1.007 [0.97; 1.044]	Low		
*ANC1*						
Bangui 1	7	Immediate effect	0.882 [0.599; 1.299]	High	− 913 [− 1286; − 535]	− 13 [− 17; − 8]
		Change in slope	0.996 [0.965; 1.027]	High		
Bangui 2	8	Immediate effect	1.014 [0.784; 1.311]	High	702 [235; 1129]	11 [1; 40]
		Change in slope	0.984 [0.945; 1.025]	High		
Bangui 3	4	Immediate effect	0.852 [0.629; 1.155]	Low	− 253 [− 585; 76]	− 5 [− 12; 4]
		Change in slope	1.022 [0.953; 1.095]	Low		
Begoua	15	Immediate effect	0.882 [0.749; 1.039]	Low	− 198 [− 435; 28]	− 4 [− 10; 3]
		Change in slope	1.018 [0.991; 1.046]	Low		
Bimbo	13	Immediate effect	**0.866 [0.755; 0.994]**	Low	− 2895 [− 4169; − 1790]	− 28 [− 35; − 19]
		Change in slope	0.997 [0.977; 1.018]	Low		
*BCG*						
Bangui 1	5	Immediate effect	1.04 [0.765; 1.415]	Low	− 1574 [− 2655; − 657]	− 24 [− 43; 4]
		Change in slope	0.963 [0.84; 1.105]	Low		
Bangui 2	3	Immediate effect	1.56 [0.326; 7.466]	Low	− 659 [− 1054; − 249]	− 22 [− 47; 485]
		Change in slope	0.847 [0.664; 1.081]	Low		
Bangui 3	5	Immediate effect	1.347 [0.86; 2.11]	Low	2399 [1599; 3056]	72 [41; 121]
		Change in slope	0.996 [0.8; 1.24]	High		
Begoua	9	Immediate effect	0.905 [0.543; 1.506]	High	− 2660 [− 3172; − 2247]	− 46 [− 52; − 41]
		Change in slope	1.003 [0.793; 1.269]	High		
Bimbo	7	Immediate effect	**1.577 [1.136; 2.19]**	Low	− 1382 [− 1989; − 796]	− 22 [− 31; − 12]
		Change in slope	**0.867 [0.802; 0.938]**	High		

*Bold cells indicate that results are statistically significant at 0.05, reflected in confidence intervals not including 1

*Outpatient Department (OPD) new consultations* showed a consistent, immediate decrease within and across health districts, although results were not statistically significant. Heterogeneity was low in Bimbo but ranged from moderate to high in other health districts. Cumulative difference ranged from − 46,000 OPD consultations in Begoua to + 7000 in Bangui 3. The average monthly change ranged from − 34% in Begoua to + 21% in Bangui 3.

*Consultations for suspected malaria* showed mixed results, and no clear trend was identified across health districts. None of the results were statistically significant. Estimates were more heterogeneous for malaria than for OPD consultations. Cumulative difference ranged from − 17,826 in Begoua to + 4345 in Bangui 3. The average monthly change ranged from − 36% in Begoua to + 35% in Bangui 2.

*RTI consultations* showed a consistent decrease at the beginning of the pandemic in all districts but Bangui 3. Although the magnitude of the decrease was quite large in some health districts (36% in Bangui 2 and 11% in Begoua), results were not statistically significant. There was a statistically significant decrease in change in slope in Begoua (by 3%). Overall, the results were in line with the decrease in new OPD consultations. Heterogeneity was low in Bangui 1 for both estimates but mixed for other health districts. Cumulative difference ranged from − 9337 in Begoua to + 301 in Bangui 1. The average monthly change ranged from − 52% in Begoua to + 742% in Bangui 2.

*Consultations for ANC1* showed a consistent decrease, but results were not statistically significant except for Bimbo (IRR: 0.866, 95%CI 0.755–0.994, corresponding to a 13% drop). Bangui 2 showed a 1% increase (not statistically significant). Heterogeneity was high in both Bangui 1 and Bangui 2, and low in Bangui 3, Begoua, and Bimbo. Cumulative difference ranged from -2895 in Bimbo to + 702 in Bangui 2. The average monthly change ranged from -28% in Bimbo to + 11% in Bangui 2.

*Delivery of BCG vaccine doses* showed an increase in four districts at the beginning of the pandemic (results were statistically significant only in Bimbo (IRR: 1.577, 95%CI 1.136–2.19, corresponding to a 58% increase)). The estimate for change in slope in Bimbo was also statically different from 1 (IRR: 0.867, 95%CI 0.802–0.938) and corresponded to a 13% decrease in trend during the COVID-19 period. The estimates for change in slope in the other health districts were close to one. The number of included health facilities was low, and model fit was poor to moderate due to high variability in pre-COVID-19 period values. Cumulative difference ranged from − 2660 in Begoua to + 2399 in Bangui 3. The average monthly change ranged from − 46% in Begoua to + 72% in Bangui 3.

3. HCWs’ perceptions of health service delivery

Most respondents reported no change or a reduction in OPD consultations, maternal and child services, and consultations for infectious and chronic diseases. From the demand side, two main reasons were mentioned: the fear of being infected with SARS-CoV-2 when visiting the health facility (which was exacerbated when several HCWs fell ill); and the reluctance to comply with preventive health measures implemented at the facility (such as physical distancing, mask wearing, handwashing); this improved over time. From the offer side, service availability was sometimes reduced due to the reallocation of human resources towards COVID-19, limiting the implementation of other services. For example, ANC frequency was reduced from three times to once per week in certain facilities; community awareness activities switched to COVID-19, removing other topics; monthly reporting was disrupted as HCWs usually in charge were mobilized for COVID-19 activities. Furthermore, the need to ensure physical distancing led to fewer people being allowed in the health facilities and increased waiting time. These factors discouraged people from seeking care. Certain services reported specific challenges. For example, HCWs reported decreased demand for routine childhood vaccinations due to reluctance and confusion about the COVID-19 vaccine. Violent acts against COVID-19 vaccinators were also reported. Pharmacies experienced stockouts due to border closures and reduced support from external partners. Laboratories noted decreased demand for tests, as they were usually linked to consultations. Furthermore, decreasing consultations led to fewer resources for the health facility to implement IPC measures and ensure services. Some HCWs reported two encouraging developments: increased awareness and focus on cleaning activities, hiring of human resources, and acquiring cleaning equipment; and increased collaboration between health facilities and the health district office due to more response coordination meetings.

4. Health care seeking behavior: community perspective

Results from the household surveys are presented in Additional file 1: Table S8. Answers are provided for the first months of the COVID-19 pandemic and the 30 days before data collection (Aug–Sep 2021). Fewer households reported an illness event during the first months of the pandemic compared to the time of data collection (24% vs. 42%). Similarly, fewer of those experiencing illness sought care (61% vs. 72%). The proportion of people who sought care when sick was higher among the elderly in both periods and among non-displaced compared to displaced (61% vs. 31% early COVID and 72% vs. 60% in Sept 2021). It was higher in rural than urban settings (73% vs. 61%) during the first months of the pandemic, but higher in urban areas at the time of data collection. Fever was the most common symptom, followed by cough, diarrhea, and chronic conditions in both periods. Fewer respondents sought care in hospitals and health facilities during the first months of the COVID-19 pandemic compared to September 2021 (hospitals: 48% vs. 66%; health facilities 25% vs. 43%), while more respondents sought care in pharmacies and from traditional healers during the first months of COVID-19 compared to September 21 (pharmacies: 16% vs. 5%; traditional healers: 14% vs. 7%). Most of the respondents who did not seek care at the time of data collection indicated the cost of treatment as the main barrier (87%), especially among female-headed households (94%) vs. male-headed (71%), displaced (93%) vs. non-displaced people (87%), in Bimbo (98%) vs. Begoua (75%) and Bangui (79%) (results in Additional file 1: Table S9). Additional file 1: Table S10 shows that higher levels of education were associated with higher odds of seeking care in both periods. During the first months of the pandemic, respondents from Bégoua and Bimbo who experienced illness also had higher odds of seeking care than respondents in Bangui. At the time of data collection, female respondents had higher odds of seeking care than males, while respondents working in agriculture had lower odds of seeking care than respondents with no employment. Results should be interpreted with caution as the number of people seeking care is small and the CI large. Most respondents (80%) reported vaccinating their children during the first months of the COVID-19 restrictions. This was consistent across age groups, sex, residence, and displacement status. Interruption of services was the most common (46%) reason for not vaccinating their children, particularly in Begoua (70%). Fear of COVID-19 infection was the second reason (27%).

In more than half of the FGDs, respondents mentioned increased reluctance to seek care due to the fear of being diagnosed with COVID-19, especially for influenza-like conditions. People did not want to comply with the restrictions the diagnosis would require (quarantine, for instance). Other reasons included limited drug availability, lack of equipment, and qualified personnel. Women in Begoua and Bimbo also reported financial barriers.

## Discussion

This study combined complementary research areas to generate a comprehensive, albeit incomplete, understanding of the situation in CAR during the first year of the COVID-19 pandemic. The COVID-19 epidemiology aligns with the global picture, including higher incidence among adult population and similar clinical presentations as in other countries [26–28]. As in many other LMICs, testing capacity was limited and test positivity high, showing a bias towards testing symptomatic people, foreigners, travelers, or specific professions that required testing to leave or enter the country (for example, truck drivers were required to test before being allowed in the country or Bangui). Some differences included more men getting tested than women, with a consequent higher incidence rate reported among men, likely due to more men traveling or working in certain professions or having better access to COVID-19 testing than women.

We noted a very large discrepancy between the number of reported COVID-19 cases from this study, regardless of whether the lowest or the highest estimate was considered, and the results of the only serosurvey (to our knowledge) conducted in Bangui over the months of July–August 2021 [29]. The estimated seroprevalence reached 74.1%, indicating a high proportion of the population with SARS-CoV-2 antibodies, although vaccination started only on May 21, 2021. While the serosurvey was conducted after the end of our study period, only a few thousand more cases were officially reported by August 31, 2021 (11,307 [30]). A seroprevalence of 74% in Bangui would correspond to circa 666,000 people with previous infection, i.e., 60 times higher than the reported cases. As about 76,000 vaccine doses were administered in the entire country [31] by the time of the seroprevalence survey in Bangui, undiagnosed and unreported cases likely explain the gap. As in other African countries, the majority of COVID-19 cases in CAR were likely asymptomatic [8], triggering no health care seeking behavior. In addition, limited testing capacity and changes in testing strategy (which from July 2020 onwards targeted only suspected cases and people at risk) excluded most of the population from testing and automatically underestimated the real case count [32]. Ministry of Health (MoH) data reported 40,541 conducted tests by March 31, 2021 [33], likely including IP and LNBCSP testing capacity. This corresponded to 824 tests/100,000 and to a 13% positivity rate, which is above the recommended 5% considered by WHO [34] as a threshold for sufficient testing capacity. Additional access barriers included the fear of testing positive, compliance with preventive measures, and cost. Polymerase chain reaction testing was free at the LNBCSP until early 2021, when travelers started paying. Testing was never free at IP, and costs were borne by individuals seeking testing. Only at the end of 2021 health districts received rapid diagnostic tests that allowed decentralized testing capacity. Ensuring quick scaling up of testing capacity will be essential for future epidemics to understand the epidemiology of the disease better. Should such rapid scale-up of testing not be possible or insufficient, a limited number of tests could be undertaken from sentinel sites across districts to improve initial understanding of disease epidemiology and case fatality rates. A more realistic knowledge of the disease mortality may allay anxiety and encourage positive health-seeking behavior.

How the pandemic affected health care utilization is poorly understood in many countries as many factors play a role, ranging from how adaptations towards clinical services were implemented, government policies on quarantine and population movement and their enforcement, individual risk perception, and how risk communication and community engagement programs were created and implemented [35, 36]. We studied health care utilization in Bangui and surrounding areas during the first year of the COVID-19 pandemic using interrupted time series models and qualitative methods amongst HCWs and citizens of CAR. We found a reduction in overall OPD consultations, and specifically for RTIs and ANC. These were noted in qualitative interviews and observed in the quantitative data. Although the results were not statistically significant, likely due to limited data availability and high variability in both pre- and COVID-19 periods, decreasing trends for these indicators were seen in most of the districts we studied in CAR. This was corroborated by fewer study participants reporting being sick and seeking care in spring 2020 than in summer 2021. While, to our knowledge, no other studies have been conducted in CAR that could provide a further understanding of our findings, similar results have been found in other LMICs [37, 38] ﻿and in humanitarian settings [39]. The fear of testing positive and complying with related restrictions was the main obstacle to seeking health care, which was reported among HCWs and community members. Similar perceptions and fears were experienced in other countries across the globe [40–42]. In addition, various measures and adaptations implemented in each health facility may have influenced the individual decision to seek or postpone care in ways that are difficult to predict. For example, small health facilities with limited resources may have had less capacity to establish triage systems, hand washing stations, or enforce preventive measures. Fewer or lax measures might have represented a deterrent for specific community members (maybe those with pre-existing conditions) or an incentive for others (maybe those who could not afford to be out of work if they tested positive). The fact that violence and population displacement following presidential elections impacted the capacity to implement COVID-19 measures adds a layer of complexity in anticipating individual behavior. These and other factors, such as proximity or the number of patients per provider, may partially explain why more people preferred seeking care in pharmacies and traditional healers instead of hospitals or health facilities during the first months of the pandemic. As seen in other epidemics [43], trust and a welcoming approach in health facilities may play a more prominent role than preventive measures in guiding individual choice.

Lack of medicine, qualified health personnel, and financial barriers were additional reasons why people reported not seeking care when sick. The 2021 CAR Humanitarian Needs Overview stated, “health care is a precious commodity that many families can no longer afford.” [44].

Health facilities located in different parts of town may have been affected differently. For example, those closer to markets may have seen a more substantial reduction in attendance when movement restrictions were implemented. Facilities in urban areas may have been more affected than rural ones, as enforcement of movement restrictions was likely higher. This was reflected in the survey results, showing that more respondents from rural areas sought care during the first months of COVID-19 restrictions compared to urban respondents. The opposite was observed at the time of data collection, possibly due to easier access in urban areas under “normal” conditions. Several health facilities also reported shifting tasks and resources towards COVID-19 prevention and treatment activities, leading to the reduction or interruption of other services and increased waiting times. The frequency of ANC decreased during the first months of the pandemic; community outreach activities first stopped and then focused primarily on COVID-19 interventions, likely reducing awareness of other health needs and routine services. As in many other countries, health facilities in CAR where HCWs fell sick with COVID-19 struggled to maintain health service provision. These are all signs of low health system resilience as health facilities showed limited adaptive capacity following a shock [45]. Besides the introduction of IPC measures, health care workers reported few health program adaptations implemented to maintain health services, although organizational guidelines were developed.

The reduction in consultations for RTIs has been observed in several countries, including Vietnam, Uganda, Kenya, Zambia, and China [46–50] and refugee settings in Jordan and Uganda [51, 52]. It was likely due to a variety of reasons, including changes in health-seeking behaviors due to difficulty in reaching health facilities, fear of being infected or of being tested, as well as an effective reduction in common RTIs due to COVID-19 related preventative measures such as masks, physical distancing, and school closures.

Cold chain and the overall delivery of routine child vaccinations were not reported as being interrupted. On the contrary, quantitative results showed increased BCG vaccine doses provided at the beginning of the pandemic, which is difficult to explain. Most respondents said they brought their children for routine vaccination at the health centers, even during the first months of the pandemic. The implementation of two vaccination campaigns (against measles and tetanus-diphtheria (Td)) was delayed by a few months, and a vaccination campaign against polio was canceled and not reinstated [53]. Several other campaigns (against Td and polio) were delayed in 2021, not because of COVID-19, but rather due to lack of funding or other implementation constraints (including post-election violence that postponed a second round of a Td campaign at the end of 2020) [54].

Despite the high variability in utilization rates within and across health services, reductions did occur with varying degrees of restoration over time. These reductions in provision, access, and utilization of health services represented an impediment towards universal coverage of essential interventions, as seen in several LMICs [55]. Their effects may have been more severe amongst populations living in fragile and conflict-affected settings.

Data availability limited our analysis. The existence of two separate COVID-19 line lists introduced variability from the beginning and likely led to discrepancies and delays. Official numbers of reported cases as of March 31, 2021, ranged from 5161 [1] [Johns Hopkins University] through 5285 [33] [MoH] up to 6316 [56] or 6360 [30] [both WHO], highlighting inconsistencies in reporting. Other inaccuracies cannot be excluded. Accurate population data are not readily available in CAR, as the last census occurred in 2003. Displacement data are often outdated as internal displacement is very fluid. As much as possible, the existence and the size of targeted displacement sites in the household survey were verified by field teams. However, certain large displacement sites were found empty in Bimbo and could not be replaced, leading to an increased margin of error for IDPs. Without a functioning electronic health information system, reporting disruptions and archiving issues may have contributed to varying levels of completeness across years and districts. Heterogeneity was high for some indicators and districts, as several factors likely affected health service provision during the study period. Furthermore, a fundamental assumption of interrupted time series is that, had the COVID-19 pandemic not happened, the long-term trends observed in the pre-COVID period would have continued. We cannot rule out that other events, such as attacks on health care or generalized conflict, may have affected the evolution of indicators during the study period. The parametric model we used assumed the COVID-19 effects could be captured with terms for immediate change and change in slope. However, the COVID-19 effects may have varied throughout the study period as different mitigation strategies were implemented. Finally, results from Bangui and surrounding areas are likely not generalizable to other urban or rural areas, given CAR’s strong regional differences in terms of service availability, access, population behavior, and security situation. Primary data collection was conducted more than one year after the end of COVID-19 restrictions in CAR and several months after a second wave of COVID-19 infections where there were no government restrictions. Recall and social desirability biases can, therefore, not be excluded. FGDs participants were purposively selected by IMPACT, likely introducing a bias.

## Conclusions

A large underestimation of infections and decreased health care utilization characterized the first year of the COVID-19 pandemic in Bangui and surrounding area. Improved decentralized testing capacity and enhanced efforts to maintain health service utilization will be crucial for future epidemics. A better understanding of health care access is needed, which will require strengthening the national health information system to ensure reliable and complete data and further research on how public health measures interact with security constraints.

## Supplementary Information

**Additional file 1.** Additional methods and results.

## Data Availability

The COVID-19 line lists and routine health data were accessed after obtaining ethical clearance and governmental authorization in CAR. Access should be requested from CAR MoH. Household survey data can be made available upon request to the authors (humanithealth@jhu.edu) once main analyses are concluded and upon submission of a concept note.

## Abbreviations

ACF: Action Contre la Faim
ANC1: Antenatal care first visit
BCG: Bacille Calmette-Guerin vaccine
CAR: Central African Republic
CI: Confidence interval
COVID-19: Coronavirus Disease 2019
FGD: Focus group discussion
HCWs: Health care workers
IP: *Institut Pasteur*
IPC: Infection prevention and control
IRB: Institutional review board
IRR: Incidence rate ratio
LMICs: Low- and middle-income countries
LNBCSP: *Laboratoire National de Biologie Clinique de Santé Publique*
MoH: Ministry of Health
OPD: Outpatient Department
RTI: Respiratory tract infections
Td: Tetanus-diphtheria
WHO: World Health Organization
