# Supplementary material for: The first year of the COVID-19 pandemic in humanitarian settings: epidemiology, health service utilization, and health care seeking behavior in Bangui and surrounding areas, Central African Republic
Source: Confl Health. 2023 May 20;17:24. doi: 10.1186/s13031-023-00523-y (PMC10199293; doi:10.1186/s13031-023-00523-y)
Supplement: Supplementary file 1 — Additional file 1. Additional methods and results. [file 13031_2023_523_MOESM1_ESM.docx]

**Supplementary Material to the article**

**“The first year of the COVID-19 pandemic in humanitarian settings: epidemiology, health service utilization, and health are seeking behavior in Bangui and surrounding areas, Central African Republic.”**

Table of Contents

[1 Methods 2](#_Toc130216193)

[1.1 Data 2](#_Toc130216194)

[1.1.1 Variables included in COVID-19 line lists 2](#_Toc130216195)

[1.1.2 Completeness of variables in the COVID-19 line lists 2](#_Toc130216196)

[1.1.3 Definition of indicators used in the ITS analysis 3](#_Toc130216197)

[1.1.4 Primary data collection methods 4](#_Toc130216198)

[1.2 Analytical approach 6](#_Toc130216199)

[1.2.1 Classification of heterogeneity in meta-analysis estimates 6](#_Toc130216200)

[1.2.2 Differences with expected values 6](#_Toc130216201)

[2 Additional results 6](#_Toc130216202)

[2.1 COVID-19 epidemiology 6](#_Toc130216203)

[2.2 Changes in health care utilization 8](#_Toc130216204)

[2.2.1 Outpatient consultations 8](#_Toc130216205)

[2.2.2 Malaria consultations 13](#_Toc130216206)

[2.3.1 All RTI 17](#_Toc130216207)

[2.3.2 ANC1 21](#_Toc130216208)

[2.3.3 BCG vaccination 25](#_Toc130216209)

[2.4 Community perspective 35](#_Toc130216210)

**List of Tables**

[Table S1: Completeness of variables in the two COVID-19 line lists. 2](#_Toc130216211)

[Table S2: Definitions of outcome indicators used in the analysis of changes in health service utilization 3](#_Toc130216212)

[Table S3: Characteristics of focus group discussions' participants 4](#_Toc130216213)

[Table S4: Surveyed population by age, district, population displacement status, Central African Republic 2021. 5](#_Toc130216214)

[Table S5: Descriptive statistics of COVID-19 cases, March 14, 2020 to March 31, 2021, CAR 6](#_Toc130216215)

[Table S6: Distribution of test results by reason for being tested (Institut Pasteur database). 7](#_Toc130216216)

[Table S7: Adjusted odds ratio for multiple risk factors for confirmed COVID-19 cases 7](#_Toc130216217)

[Table S8: Household survey results about health care seeking behavior, comparing reported behavior at the beginning of the COVID-19 pandemic and the month before the survey, CAR 35](#_Toc130216218)

[Table S9: Barriers for not seeking care during the month before data collection (Aug-Sept 2021), CAR 36](#_Toc130216219)

[Table S10: Factors associated with seeking care during the first months of the COVID-19 pandemic and at the time of data collection, CAR 37](#_Toc130216220)

**List of Figures**

[Figure S1: Epidemiological curve of COVID-19 cases in CAR from March 14, 2020 to March 31, 2021 8](#_Toc130216221)

# Methods

## Data

### Variables included in COVID-19 line lists

The anonymized line lists included the following individual level information (although definitions differed between the two lists and completeness varied):

- Patient demographic information: age, sex, nationality, residence, address, district;
- Clinical presentation: date of first symptom, cough, fever, sore throat, dyspnea, chills, headache, loss of taste and smell, diarrhea;
- Information related to the test: dates of sample collection, test, results, whether patient was asymptomatic at sample collection, reason for being tested, site of sample collection (nasal or oropharyngeal);
- RT-PCR testing results: negative and positive in IP dataset; only positive in the LNBCSP dataset;
- Presence of comorbidities or other underlying conditions; and
- Exposure risks (travels, suspect contact with a confirmed case, country, local or imported case).

Both line lists included a "Comments" field, which captured details about travel, origin and some comorbidities. They were used to define the following two variables: i) “Travel” (Yes/No) meaning a patient who had been travelling (no details about timing or destination available); ii) “origin” (the country or city where patient came from).

### Completeness of variables in the COVID-19 line lists

Table S1: Completeness of variables in the two COVID-19 line lists.

| Variable | Institute Pasteur | National Laboratory |
| --- | --- | --- |
| N of cases included in each data set | 25,187 | 3,340 |
| Unique Code |  | 100% |
| Health region |  | 0.8% |
| Health district |  | 0.83% |
| Facility |  | 0.23% |
| Origin |  | 99.43% |
| Address | 99.72% |  |
| Sex | 99.64% | 98.74% |
| Age | 95.68% | 94.88% |
| Entry number |  | 100% |
| Provider |  | 89.28% |
| Phone |  | 0.45% |
| Nationality |  | 0.89% |
| Date of symptoms onset | 6.39% | 0.02% |
| Symptomatic |  | 19.13% |
| Temperature | 77.03% | 10.47% |
| Headache | 94.93% | 19.13% |
| Short breath | 93.66% | 19.13% |
| Cough | 94.79% | 19.13% |
| Sore throat | 94.22% | 19.13% |
| Chills | 93.97% | 19.13% |
| Ageusia | 92.91% | 19.16% |
| Anosmia | 92.92% | 19.13% |
| Ageusia/anosmia |  |  |
| Diarrhea |  | 19.13% |
| Comorbidity | 0.48% |  |
| Date of sample collection | 99,88% | 100% |
| Reception date of sample | 23.89% |  |
| Time between COVID-19 test and result | 1.30% |  |
| Result of PCR sars Cov-2 | 99.95% |  |
| Date of PCR | 99.94% |  |
| Comments | 99.97% | 45.71% |
| Influenza/ others respiratory virus | 0.56% |  |
| Case contact | NA |  |
| Initial sample/no | 33.44% | 99.16% |
| Suspect cases |  | 19.13% |
| Urgent cases |  | 19.13% |
| Post mortem cases |  | 19.13% |
| Travel |  | 42.21% |
| Confirmed case contact |  | 19.13% |
| International trip |  | 19.13% |
| Others |  | 0.30% |
| Control |  | 99.16% |
| Country |  | 0.02% |
| Local or imported cases |  | 96.46% |
| Arrive date in CAR | 21.74% | 5.32% |
| Date of last contact with confirmed case |  | 5.95% |
| Site of sample |  | 100% |
| N GENE |  | 100% |
| Ct1 |  | 94.37% |
| DATE1 |  | 100% |
| ORF1ab |  | 100% |
| CT2 |  | 97.24% |
| DATE2 |  | 100% |
| CONCLUSION |  | 45.71% |

### Definition of indicators used in the ITS analysis

Table S2: Definitions of outcome indicators used in the analysis of changes in health service utilization

| **Indicator name** | **Definition** |
| --- | --- |
| Outpatient consultations | Number of outpatient consultations, monthly |
| ANC1 coverage | Number of first antenatal care consultations, monthly |
| Bacille Calmette-Guerin (BCG) vaccination | Number of children under 11 months of age who received the BCG vaccine dose |
| Respiratory tract infections (RTI) | Number of consultations for respiratory tract infections, monthly |
| Malaria consultations | Total number of consultations for malaria (including severe and non-severe), monthly |

### Primary data collection methods

Data collection took place between September 6 and 24, 2021.

#### Qualitative data collection

Qualitative data was collected via 24 semi-structured FGDs, with approximately six to eight participants for a total of 192 participants. The groups were stratified by sex, age group and vulnerability (see table S3 for details).

FGD participants were selected in consultation with community leaders, local authorities, and non-governmental organizations (NGOs) to ensure that participants were capable of providing precise and reliable information. To ensure a variety of opinions, key informants (for example local chiefs, community and religious leaders, leaders of women’s and youth associations, camp representatives, merchants, or local authorities) as well as community members, participated in FGDs.

Six FGDs were conducted in each of the three targeted districts with men and women of two age groups (18-30 and 31-59 years). In addition, two FGDs in each district were comprised of vulnerable persons: either those over the age of 60 years or those considered vulnerable by their community (e.g., those living with chronic illnesses, physically disabled, or pregnant and nursing women).^[[1]](#footnote-1)^ The locations of FGDs varied: six FGDs were in displacement camps, six in rural zones and 12 in urban zones. FGDs lasted on average 150 minutes.

Table S3: Characteristics of focus group discussions' participants

| **Health District** | **Area Type** | **Sex** | **Age (yr) / Category** | **Status** | **# FGD**  **participants** |
| --- | --- | --- | --- | --- | --- |
| Bégoua | Urban | Female | 31-59 | non-displaced | 7 |
| Bégoua | Urban | Male | 18-30, 31-59 | non-displaced | 8 |
| Bégoua | Rural | Mixed | 60+ | non-displaced | 8 |
| Bégoua | Rural | Male | 31-59 | non-displaced | 10 |
| Bégoua | Urban | Female | 31-59 | non-displaced | 7 |
| Bégoua | Urban | Female | 18-31, Pregnant, nursing Female | non-displaced | 7 |
| Bégoua | Rural | Male | 31-59 | non-displaced | 10 |
| Bégoua | Rural | Female | 31-59 | non-displaced | 8 |
| Bimbo | Rural | Male | 18-30 | non-displaced | 9 |
| Bimbo | Rural | Female | 31-59 | non-displaced | 8 |
| Bimbo | Urban | Male | 18-30 | IDPs (site) | 8 |
| Bimbo | Urban | Female | 31-59 | IDPs (site) | 9 |
| Bimbo | Urban | Male | 31-59 | non-displaced | 8 |
| Bimbo | Urban | Female | 18-30 | non-displaced | 9 |
| Bimbo | Urban | Mixed | 60+ | non-displaced | 6 |
| Bimbo | Urban | Mixed | Vulnerable | non-displaced | 4 |
| Bimbo | Urban | Female | 31-59 | IDPs (site) | 8 |
| Bangui | Urban | Female | 18-30 | IDPs (site) | 8 |
| Bangui | Urban | Mixed | 60+ | IDPs (site) | 9 |
| Bangui | Urban | Male | 31-59 | IDPs (site) | 9 |
| Bangui | Urban | Male | 31-59 | non-displaced | 8 |
| Bangui | Urban | Male | 18-30 | non-displaced | 8 |
| Bangui | Urban | Mixed | Vulnerable | non-displaced | 8 |
| Bangui | Urban | Female | 18-30 | non-displaced | 8 |

#### Quantitative data collection

Sampling was carried out on two levels. The first level was represented by localities (districts and villages): the number of households to survey in each locality was identified with a probability proportional to the population size of the locality. Secondly, individual households within each locality were selected via random allocation of a GPS point per household. Sampling was stratified by location (rural or urban) and displacement status. Sample size was calculated for a +/- 5% margin of error at a 95% confidence level.

Population data created by Facebook and the Center for International Earth Science Information Network from April 2018,^[[2]](#footnote-2)^ supplemented by census data of displacement camps conducted by the Camp Coordination and Camp Management (CCCM) Cluster (some of them checked and updated by REACH before data collection) were used to inform this sampling process.

Final sample size for the household survey comprised 1,045 households. See table S4 for details of the surveyed population.

Table S4: Surveyed population by age, district, population displacement status, Central African Republic 2021.

|  | **Bangui** | | | **Bégoua** | | **Bimbo** | | | | **Total** |
| --- | --- | --- | --- | --- | --- | --- | --- | --- | --- | --- |
|  | **Urban** | | | **Rural** | **Urban** | **Rural** | **Urban** | | |  |
|  | **IDP** | **Res** | **Total** | **Res** | **Res** | **Res** | **IDP** | **Res** | **Tot** |  |
| **18-29** | **29** | **89** | **118** | **34** |  | **67** | **18** | **34** | **52** | **271** |
| Female | 28 | 64 | 92 | 24 |  | 50 | 15 | 27 | 42 | 208 |
| Male | 1 | 25 | 26 | 10 |  | 17 | 3 | 7 | 10 | 63 |
| **30-59** | **77** | **218** | **295** | **99** | **2** | **149** | **54** | **63** | **117** | **662** |
| Female | 41 | 129 | 170 | 53 | 1 | 83 | 26 | 41 | 67 | 374 |
| Male | 36 | 89 | 125 | 46 | 1 | 66 | 28 | 22 | 50 | 288 |
| **60+** | **3** | **40** | **43** | **10** |  | **34** | **9** | **16** | **25** | **112** |
| Female | 1 | 22 | 23 | 4 |  | 13 | 6 | 8 | 14 | 54 |
| Male | 2 | 18 | 20 | 6 |  | 21 | 3 | 8 | 11 | 58 |
| **Grand Total** | **109** | **347** | **456** | **143** | **2** | **250** | **81** | **113** | **194** | **1045** |

Data was collected on tablets with ODK technology.

## Analytical approach

### Classification of heterogeneity in meta-analysis estimates

We include a classification of the level of heterogeneity for each estimate in the results. While acknowledging that each heterogeneity statistic is difficult to interpret on its own, we attempted to classify heterogeneity using I^2^ statistic and p-value for the Chi2 test for heterogeneity, roughly following the suggested interpretation in the Cochrane handbook.[15] Thus, if p-value <0.10, or I2>50%, we classified this as having high evidence of heterogeneity (“high heterogeneity”). If 0.10≤p-value<0.20 40%<I2≤50%, we classified this as “moderate heterogeneity”; and if I2<40%, and p-value ≥0.20, we considered this as low evidence of heterogeneity (“low heterogeneity”).

### Differences with expected values

We calculate two measures of the difference with expected values: 1) the cumulative difference between observed and expected number of consultations (by type) over the study period; and 2) the average monthly percent change in consultations for each month of the COVID-19 period and at each facility within each health zone.

To do so, we first generated 1,000 predicted values (“expected”) had there been no changes during the COVID-19 period using the base model, after setting the and terms to 0. If values less than 1 were generated, they were replaced with 1, as we expected at least one consultation at each health facility on a monthly basis. For months where observed data were missing, we imputed 1,000 possible observed values from the predicted mean and standard error from the base model. Difference between observed and expected values at each facility for each month was calculated for the 1,000 draws. For each of the draws, the cumulative difference between observed and expected values was calculated by summing up the difference from each of the health facilities in the health zone. From these 1,000 differences, median, 2.5^th^, and 97.5^th^ quantiles were obtained. To calculate the average monthly percentage difference at health zone level, for each of the 1000 draws, for each month, we calculated the percent difference between cumulative observed and cumulative expected number of consultations for specific service. For each of the draws, we then obtained the average percent difference. The median, 2.5^th^, and 97.5^th^ quantiles were then obtained for the average percent difference.

# Additional results

## COVID-19 epidemiology

Table S5: Descriptive statistics of COVID-19 cases, March 14, 2020 to March 31, 2021, CAR

|  | **National laboratory** | | **Institute Pasteur** | |
| --- | --- | --- | --- | --- |
| **Total** | 3,339 | | 3,992 | |
| **Sex** |  | |  | |
| Female | 916 (27.8%) | | 1037 (26.1%) | |
| Male | 2382 (72.2%) | | 2941 (73.9%) | |
| **Age** (mean + sd) [ range] | 37.7 ± 13.7 [1, 131] | | NA | |
| Most affected age groups | 30-39 (32.3%) | | NA | |
| **Residence** |  | |  | |
| Bangui | 2,692 (81.2%) | | 2,458 (66.2%) | |
| Bimbo | 13 (0.4%) | | 244 (6.6%) | |
| Begoua | 211 (6.4%) | | 133 (3.6%) | |
| Other cities | 391 (11.8%) | | 427 (11.5%) | |
| Other countries | 9 (0.3%) | | 451 (12.1%) | |
| **Symptomatic** |  | |  | |
|  | Female | Male | Female | Male |
| Fever | 804 (27.1%) | 2163 (72.9%) | 453 (11.4%) | 1,108 (27.8%) |
| Cough | 60 (35.5%) | 109 (64.5%) | 187 (5.1%) | 463 (12.57%) |
| Chills | 36 (34.6%) | 68 (65.4%) | 80 (2.2%) | 138 (3.8%) |
| Dyspnea | 26 (28.3%) | 66 (71.7%) | 57 (1.6%) | 218 (6%) |
| Loss of taste | 9 (45%) | 11 (55%) | 58 (1.7%) | 91 (2.7%) |
| Loss of smell | 4 (30.8%) | 9 (69.2%) | 43 (1.3%) | 82 (2.4%) |
| Sore throat | 35 (38.9%) | 55 (61.1%) | 94 (2.6%) | 179 (4.9%) |
| Overall | 127 (30.4%) | 291 (69.6%) | 257 (6.5%) | 787 (19.8%) |

Table S6: Distribution of test results by reason for being tested (Institut Pasteur database).

|  | PCR test conducted | | | | | p-value |
| --- | --- | --- | --- | --- | --- | --- |
|  | Negative | Invalid | Positive | Total |  | |
| N | 21,057 | 125 | 3,922 | 25,188 |  | |
| Travel (N= 12,132) |  |  |  |  |  | |
| Yes | 6,776 (88.7%) | 3 (0.0%) | 858 (11.2%) | 7,637 (100%) | p< 0.0001 | |
| No | 3,434 (76.4%) | 116 (2.58%) | 945 (21.0%) | 4,495 (100%) |  | |
| Being a contact (N=3,875) |  |  |  |  | p< 0.0001 | |
| Yes | 3,040 (78.5%) | 106 (2.7%) | 729 (18.8%) | 3,875 (100%) |  | |
| No | 18,014 (84.6%) | 19 (0.1%) | 3,262 (15.3%) | 21,295 (100%) |  | |

Table S7: Adjusted odds ratio for multiple risk factors for confirmed COVID-19 cases

| Risk factors for positive test results | Odds Ratio | p | 95% CI |
| --- | --- | --- | --- |
| Male (Ref: female) | 1.14 | 0.049 | 1.0 – 1.3 |
| Residence (Ref: Bangui) |  |  |  |
| Bimbo | 1.17 | 0.19 | 0.92 – 1.48 |
| Begoua | 0.66 | 0.007 | 0.48 – 0.89 |
| Other cities | 1.02 | 0.76 | 0.87 – 1.21 |
| Other countries | 2.52 | <0.0001 | 2.15 – 2.94 |
| Symptomatic (Ref: No symptom) | 1.57 | <0.0001 | 1.36 – 1.81 |
| Travel (Ref: no travel) | 0.36 | <0.0001 | 0.31 – 0.41 |

Figure S1: Epidemiological curve of COVID-19 cases in CAR from March 14, 2020 to March 31, 2021

## Changes in health care utilization

### Outpatient consultations

#### Bangui 1

Note: we excluded Mama Carla from analysis, because of unreasonable counterfactual.


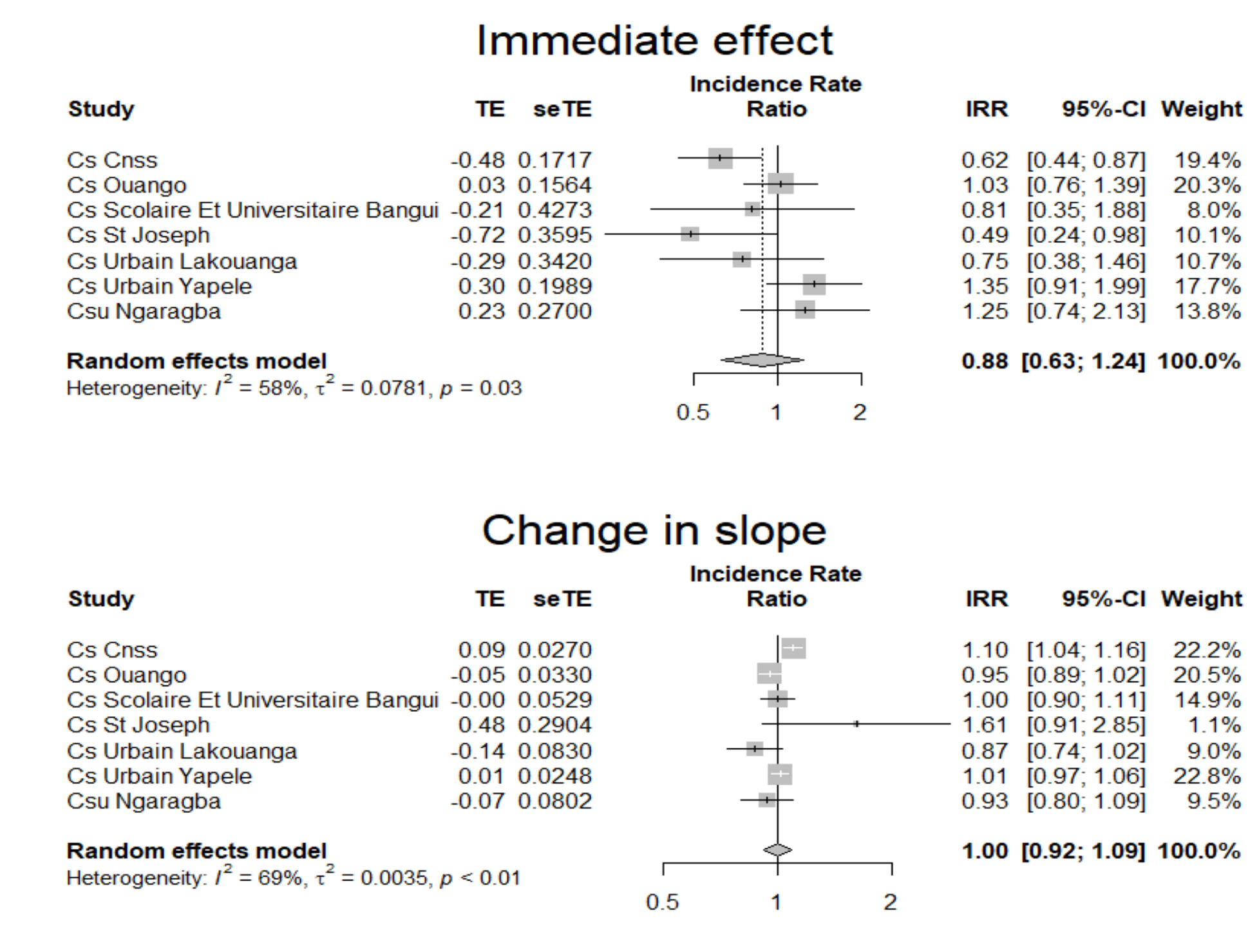


#### Bangui 2


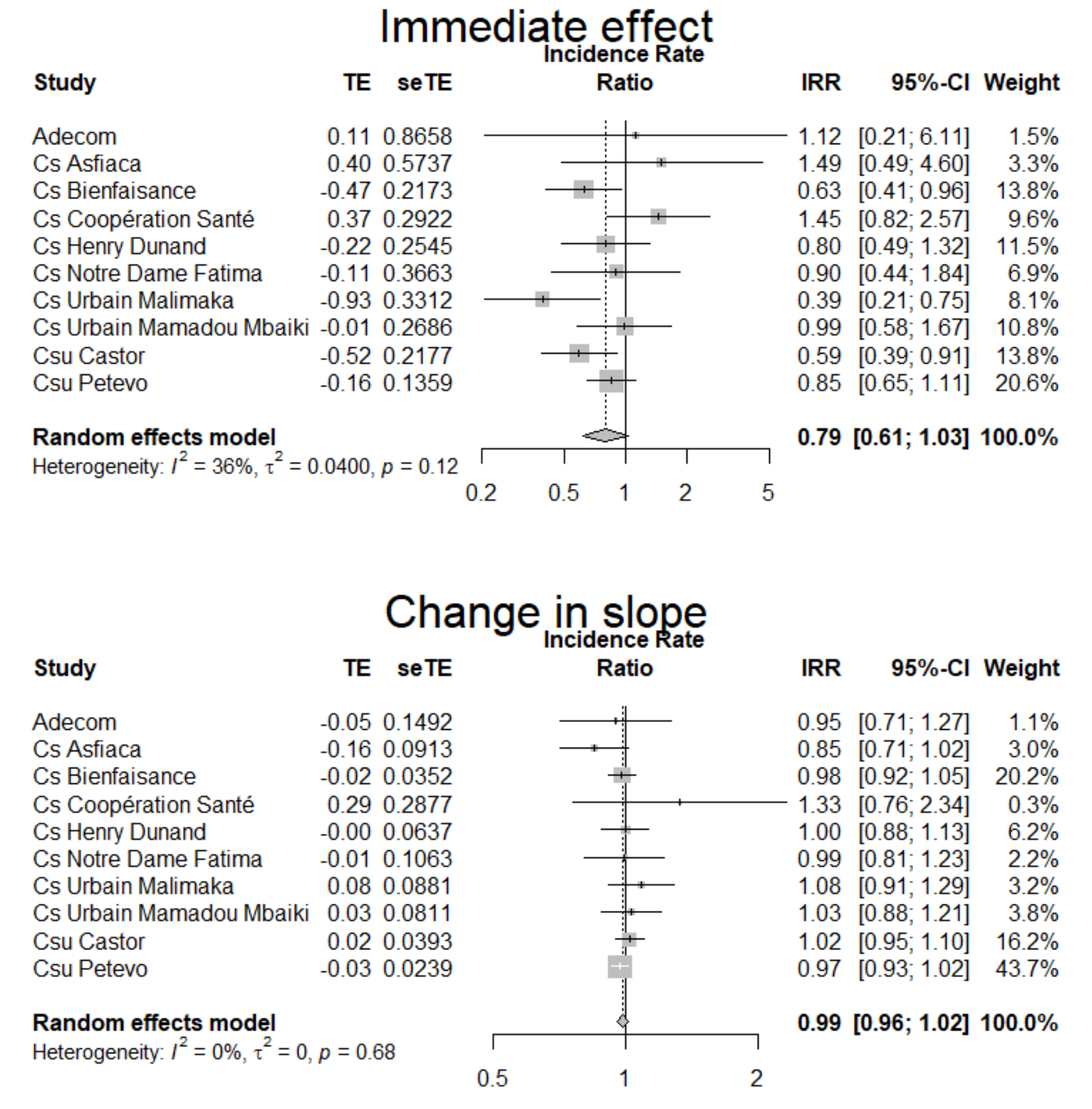


#### Bangui 3

Note: we excluded CS SAINT LUC MONASTERE and CSU BEDE COMBATTANT because of unreasonable counterfactual


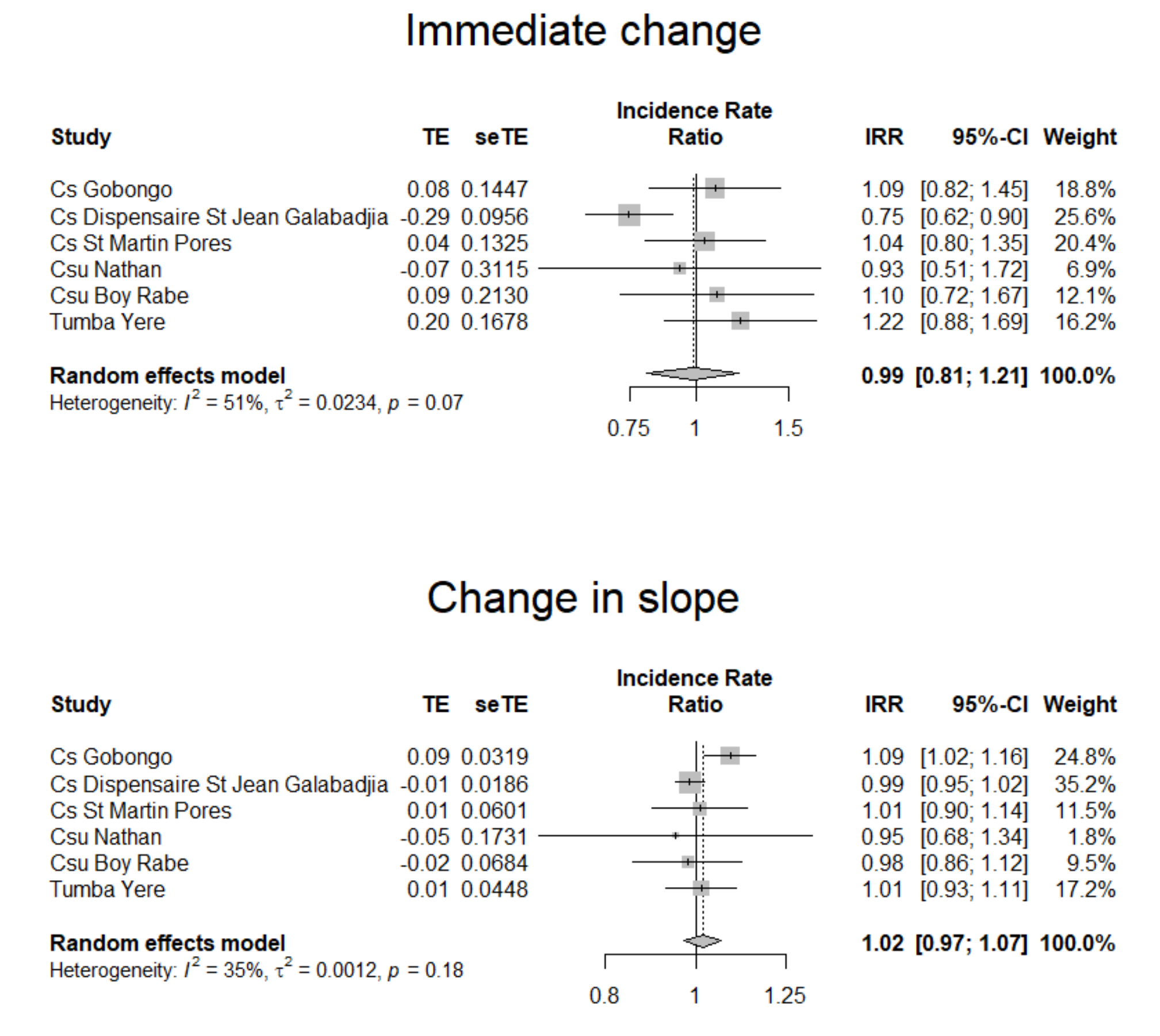


#### Begoua

Note: we excluded CS IMOHORO because it was missing data for 12 months before COVID period and first few months of COVID period; CS TREE-STRAND because of unreasonable counterfactual


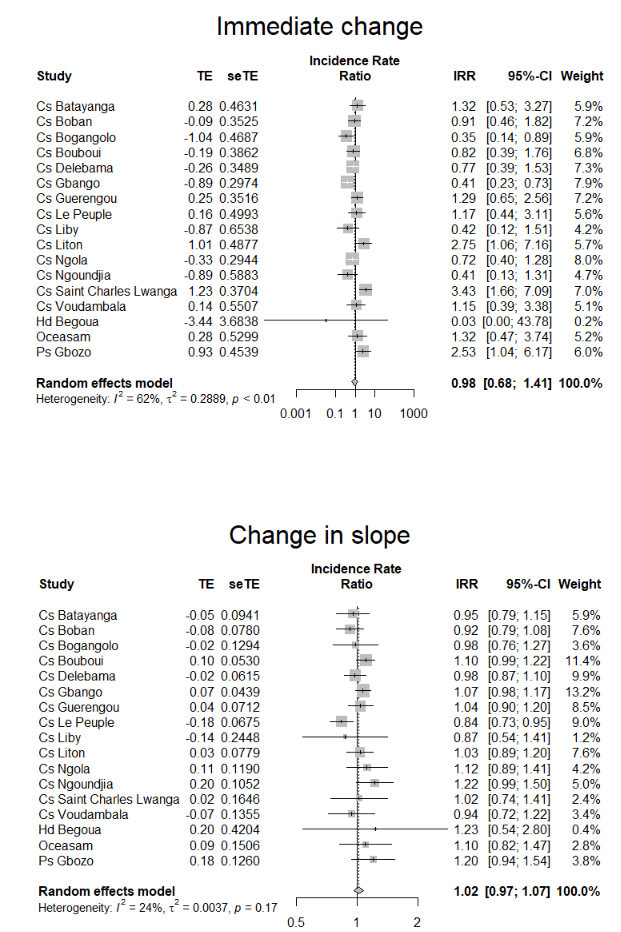


#### Bimbo

Note: excluded CS SALANGA because of unreasonable counterfactual


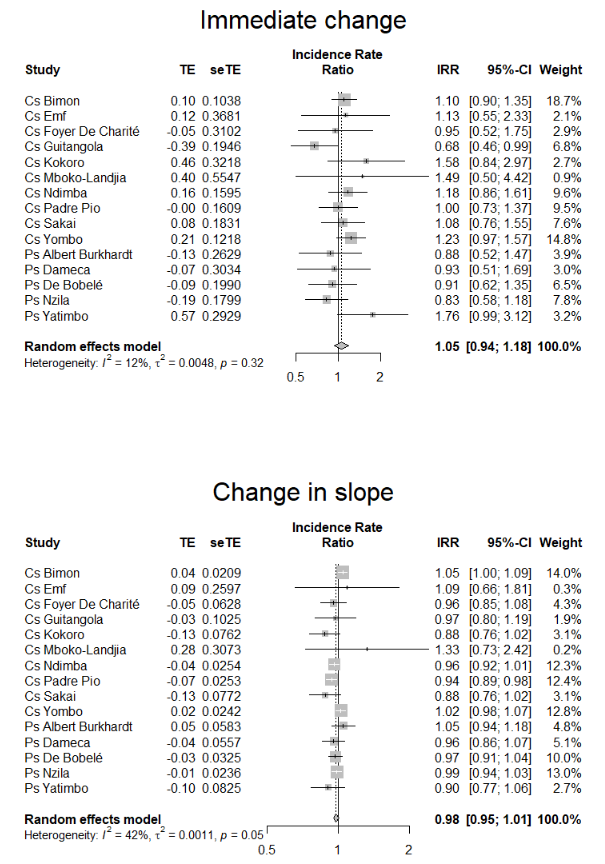


### Malaria consultations

#### Bangui 1

Note: excluded MAMA CARLA because of unreasonable counterfactual


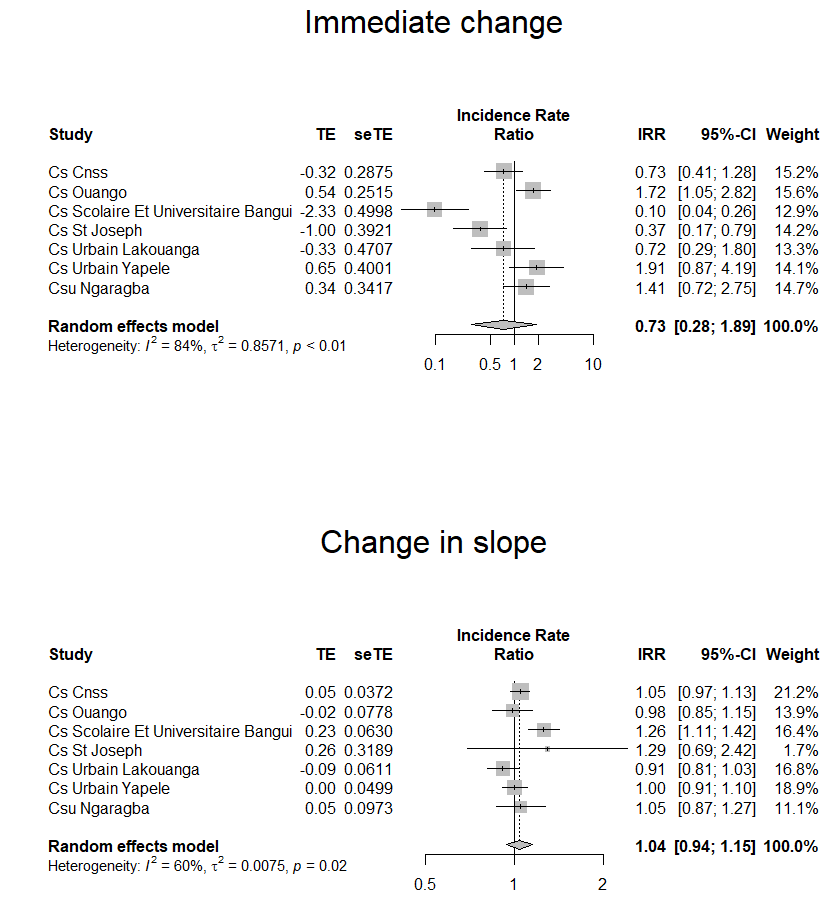


#### Bangui 2


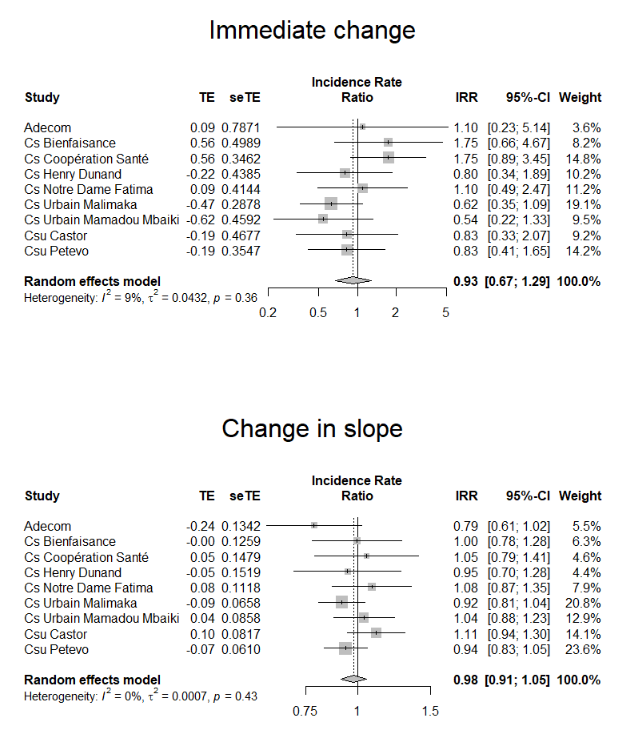


#### Bangui 3


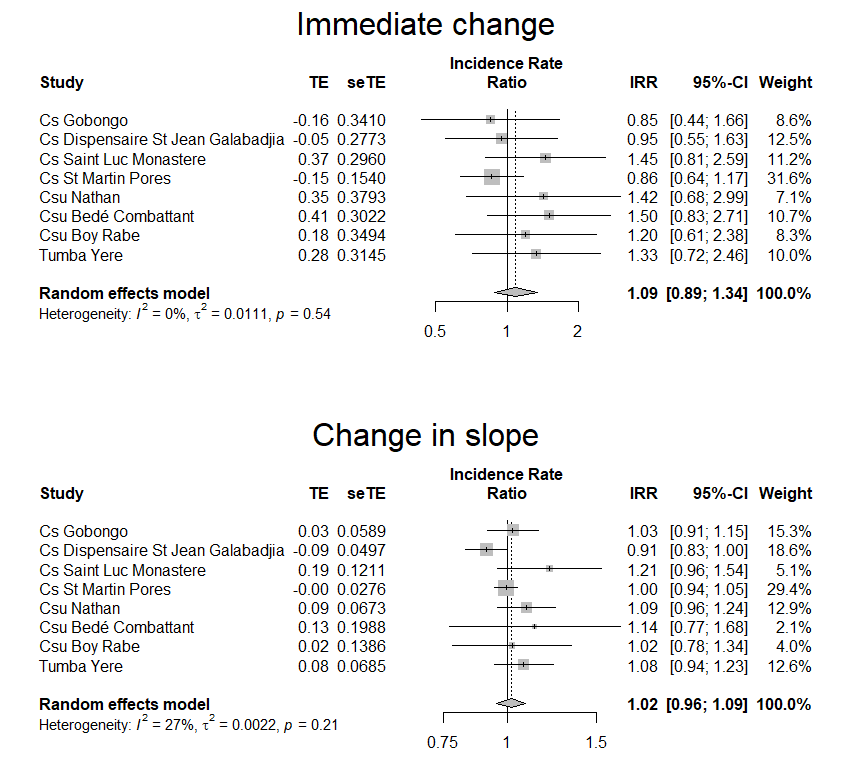


#### Begoua

Note: excluded CS IMOHORO because missing all of 2019 and 2020 data


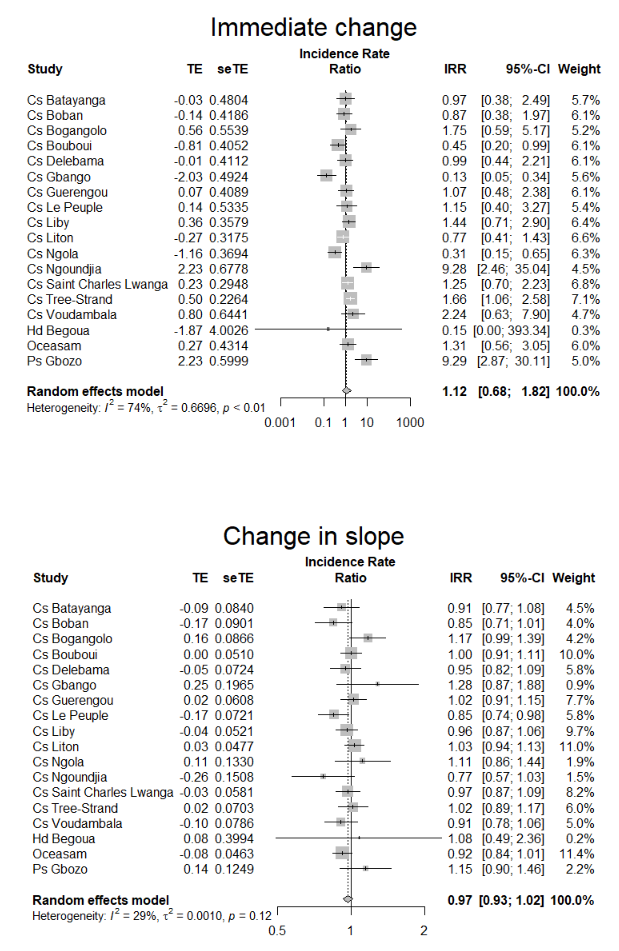


#### Bimbo


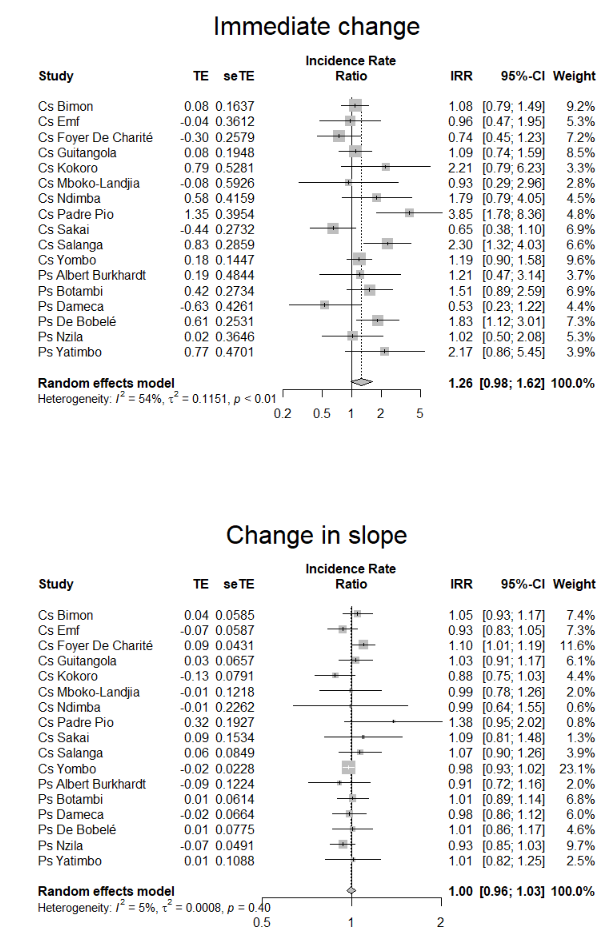


### All RTI

#### Bangui 1

Note: removed CS Ubrain Lalounga because of unreasonable counterfactual, and CS Scolaire et universitaire because the RTI consultations were too few


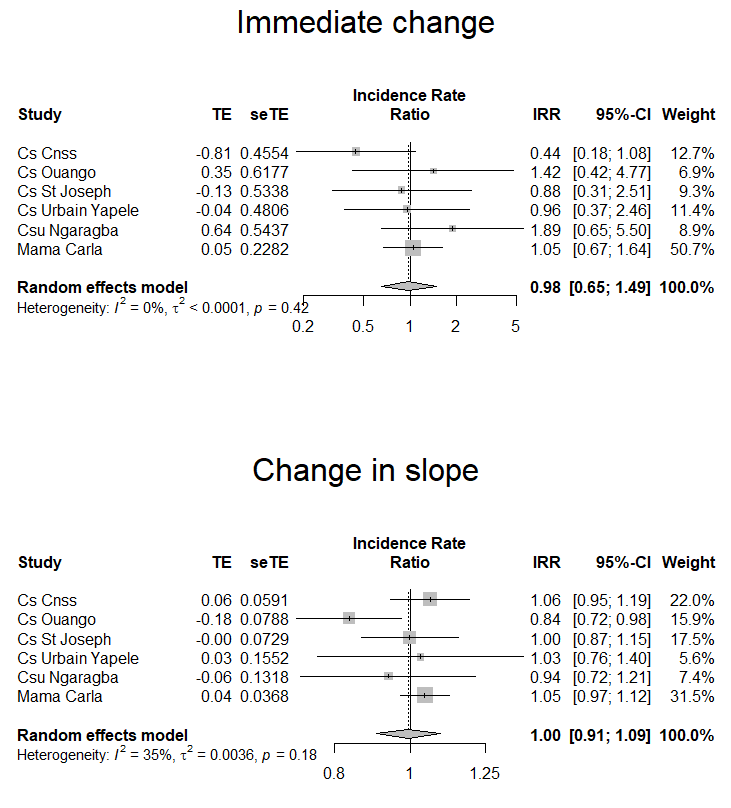


#### Bangui 2


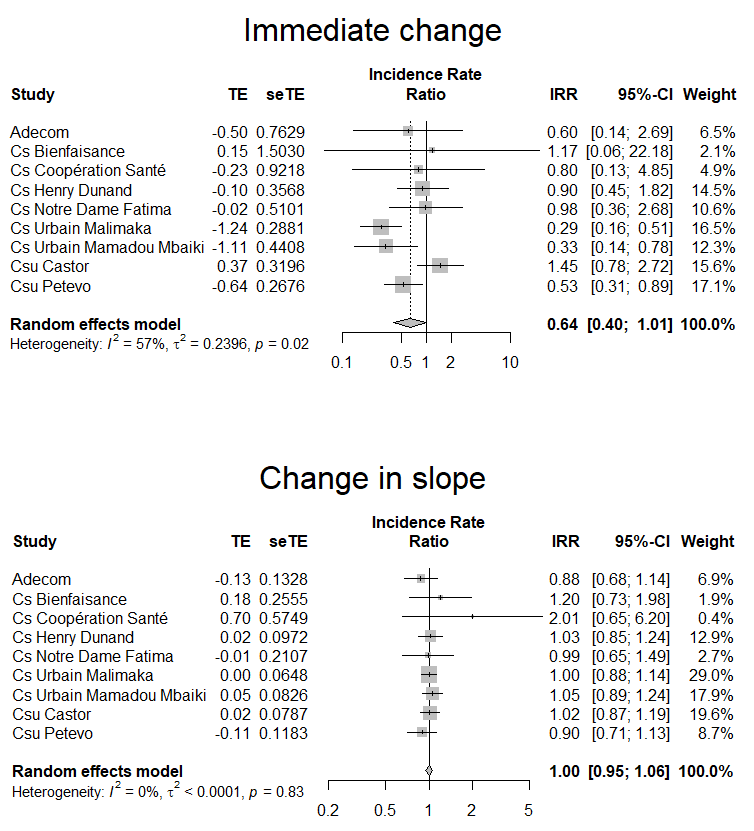


#### Bangui 3

Note: Excluded TUMBA YERE because too small volume of consultations


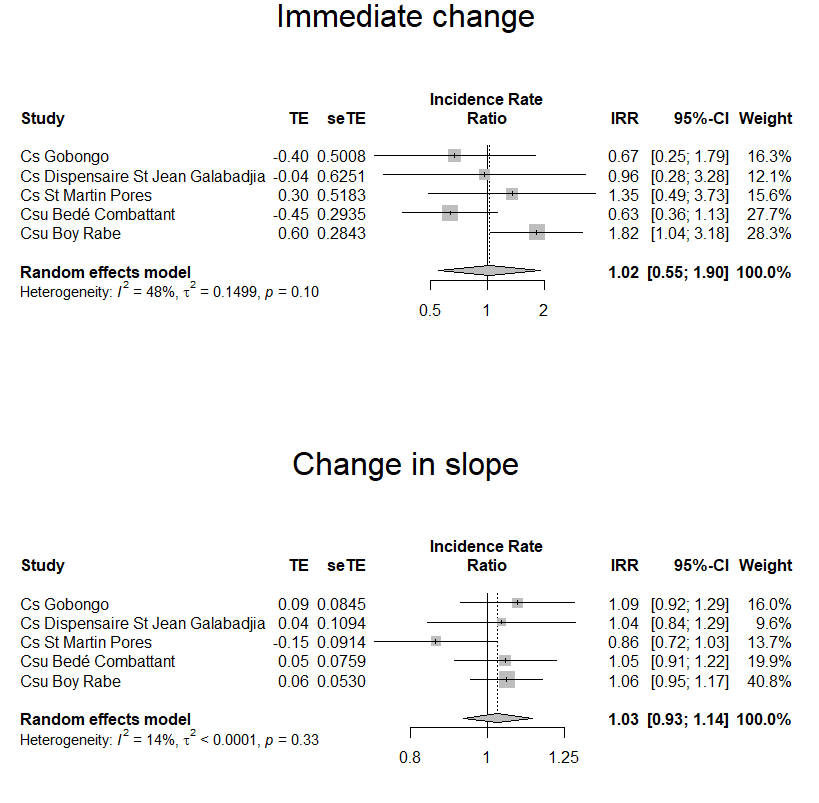


#### Begoua

Note: CS Boban and CS Bogangolo were excluded.


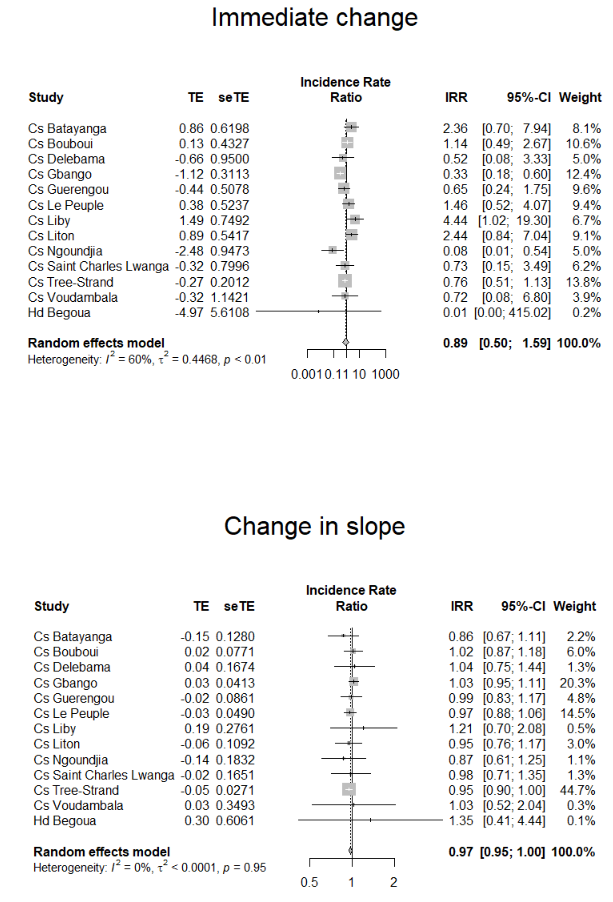


#### Bimbo


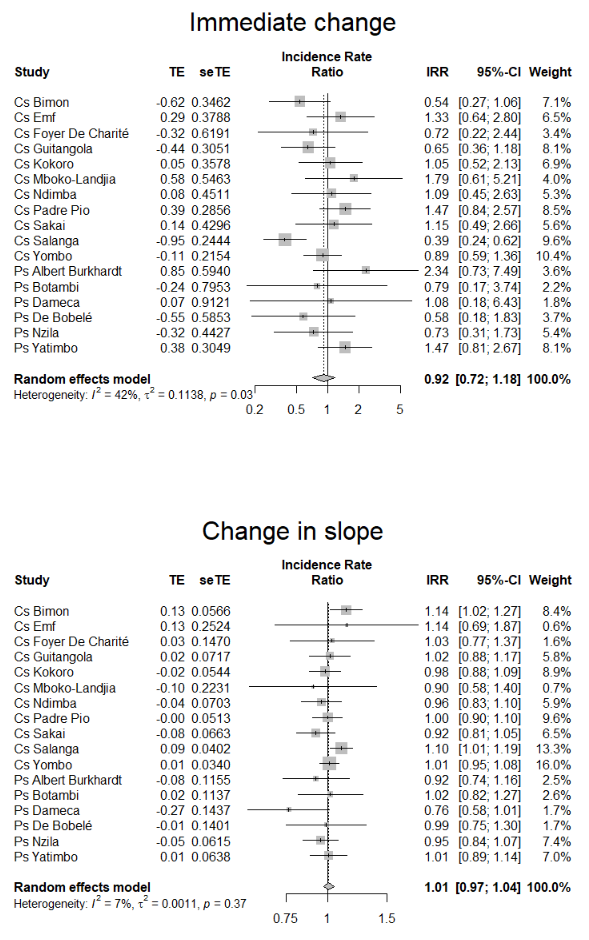


### ANC1

#### Bangui 1


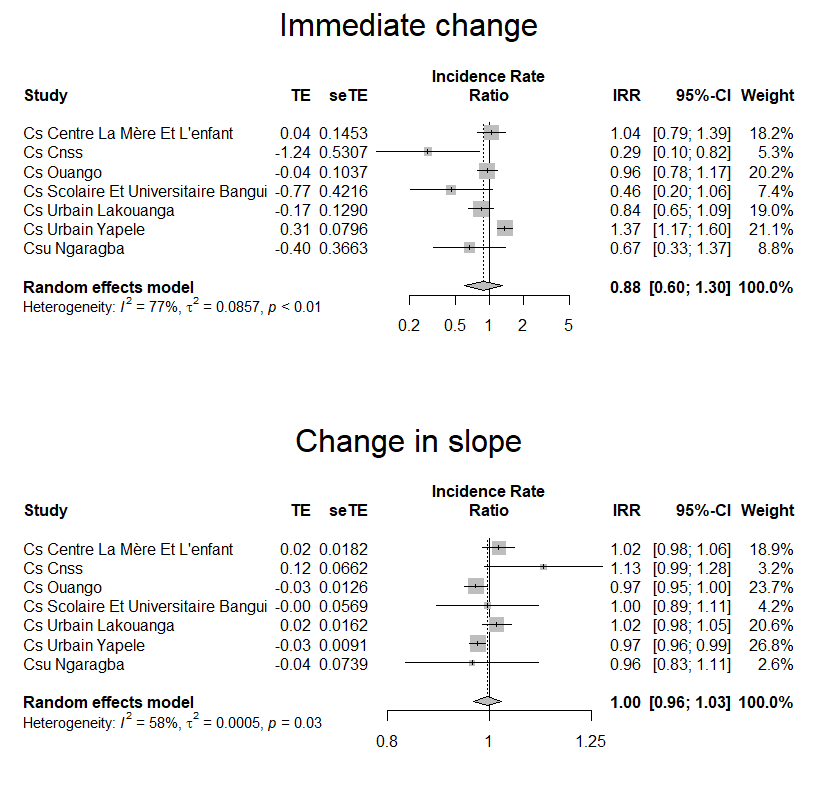


#### Bangui 2

Note: excluded CS BIENFAISANCE, CS HENRY DUNAND because of poor fit


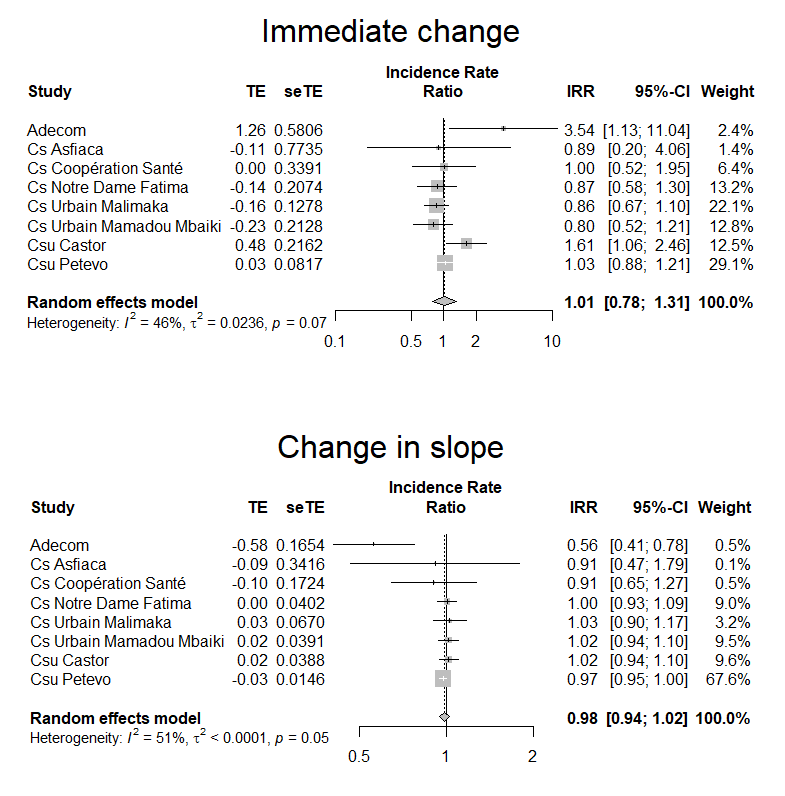


#### Bangui 3

Note: excluded CS DISPENSAIRE ST JEAN GALABADJIA. CSU NATHAN, CSU BEDE COMBATTANT, TUMBA YERE because of poor fit


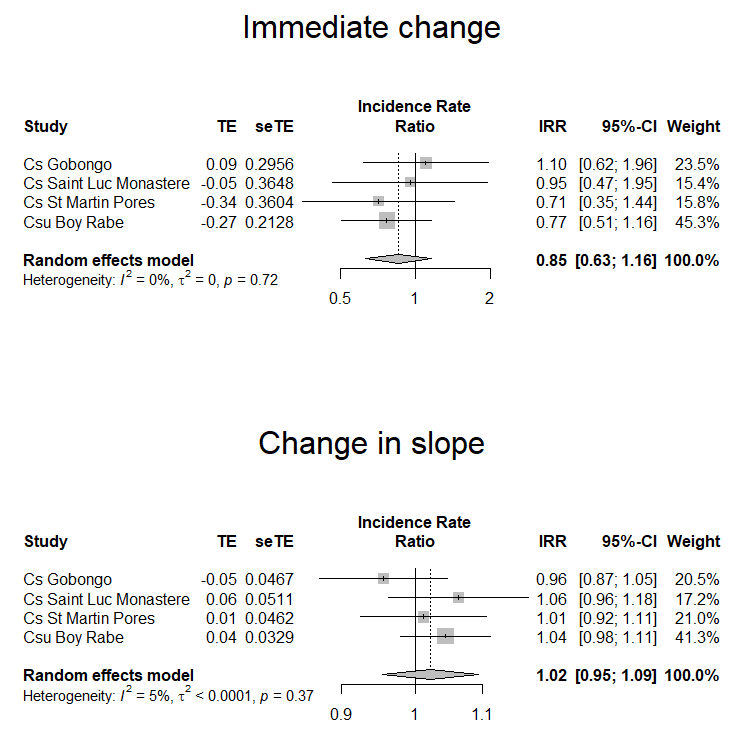


#### Begoua

Note: excluded CS LE PEUPLE (more than 1 year of data missing immediately pre-COVID) and HD BEGOUA (missing all COVID-period data in 2020)


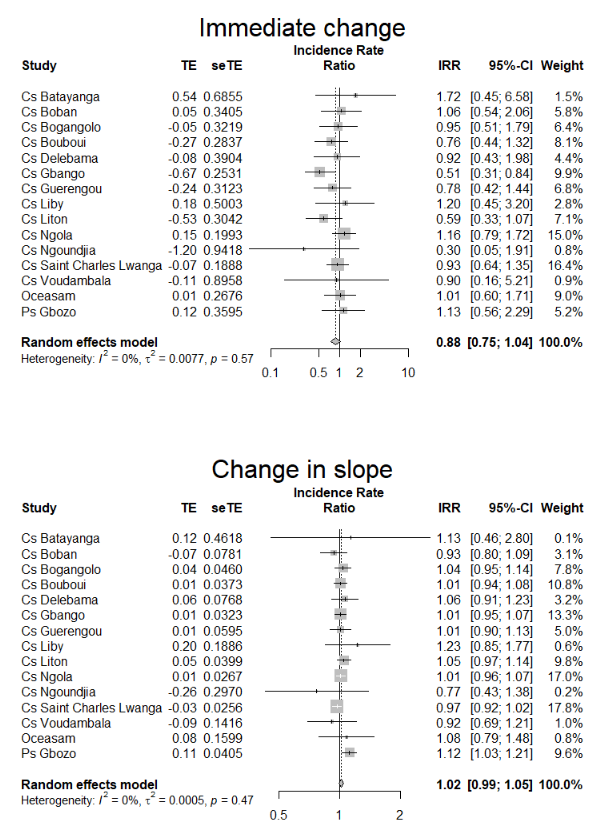


#### Bimbo

Note: Excluded PS NZILA because missing all COVID 2020 data; CS FOYER DE CHARITÉ and CS SAKAI because of unreasonable estimates


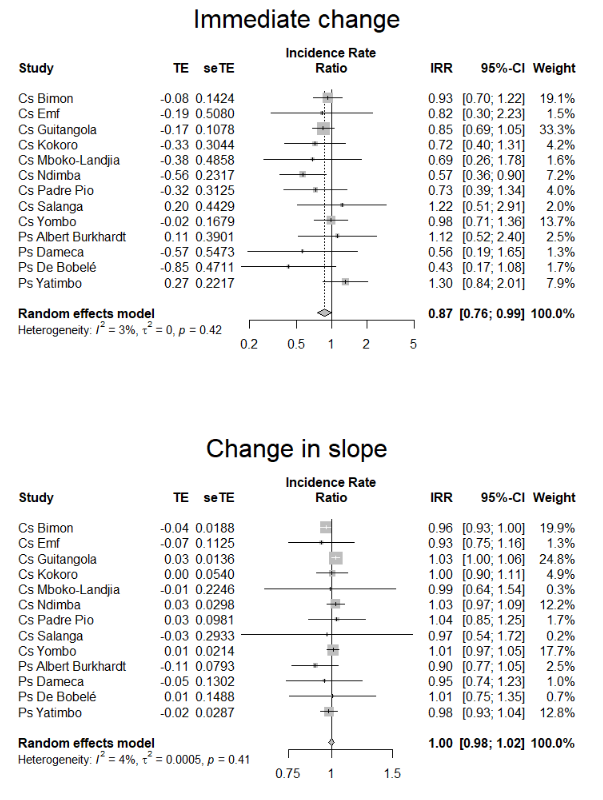


### BCG vaccination

#### Bangui 1


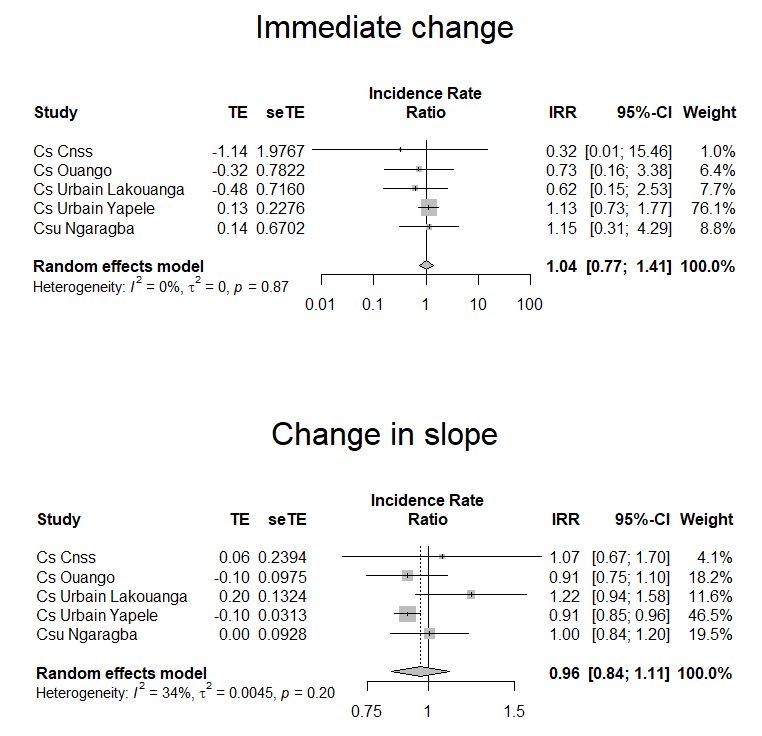


Model fit and residuals

| Facility | Model fit | Residuals |
| --- | --- | --- |
| CS CNSS | 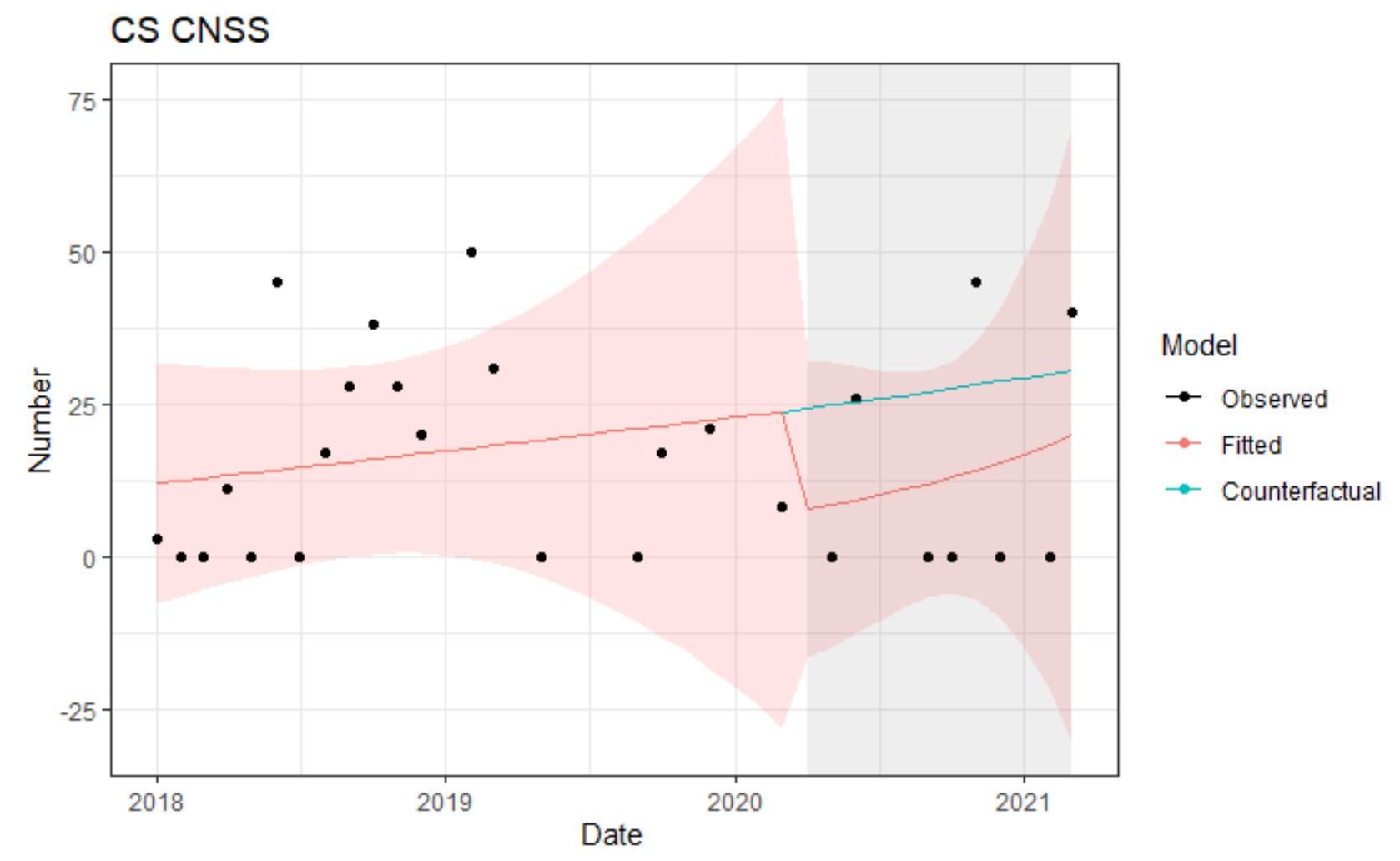 | 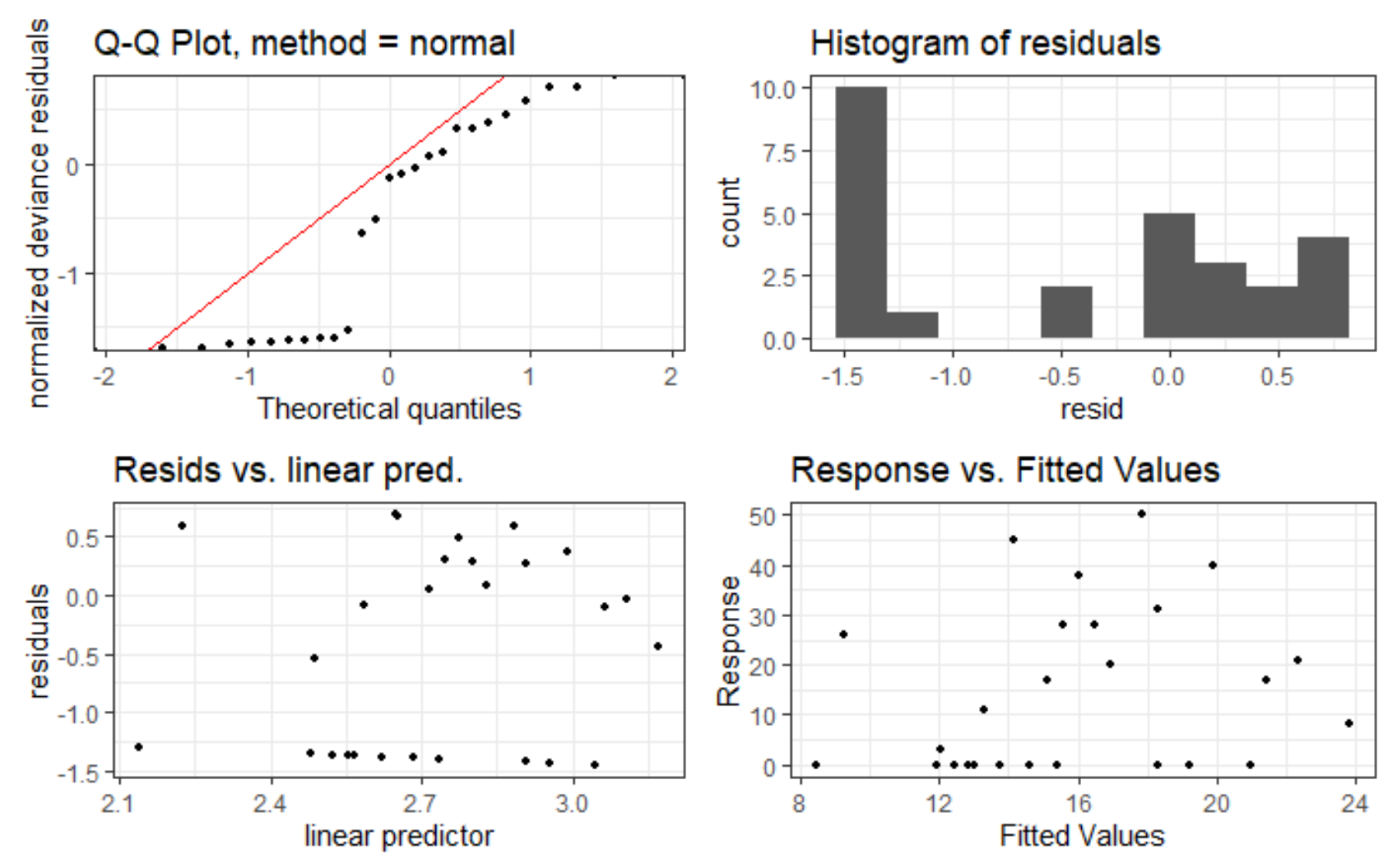 |
| CS Ouango | 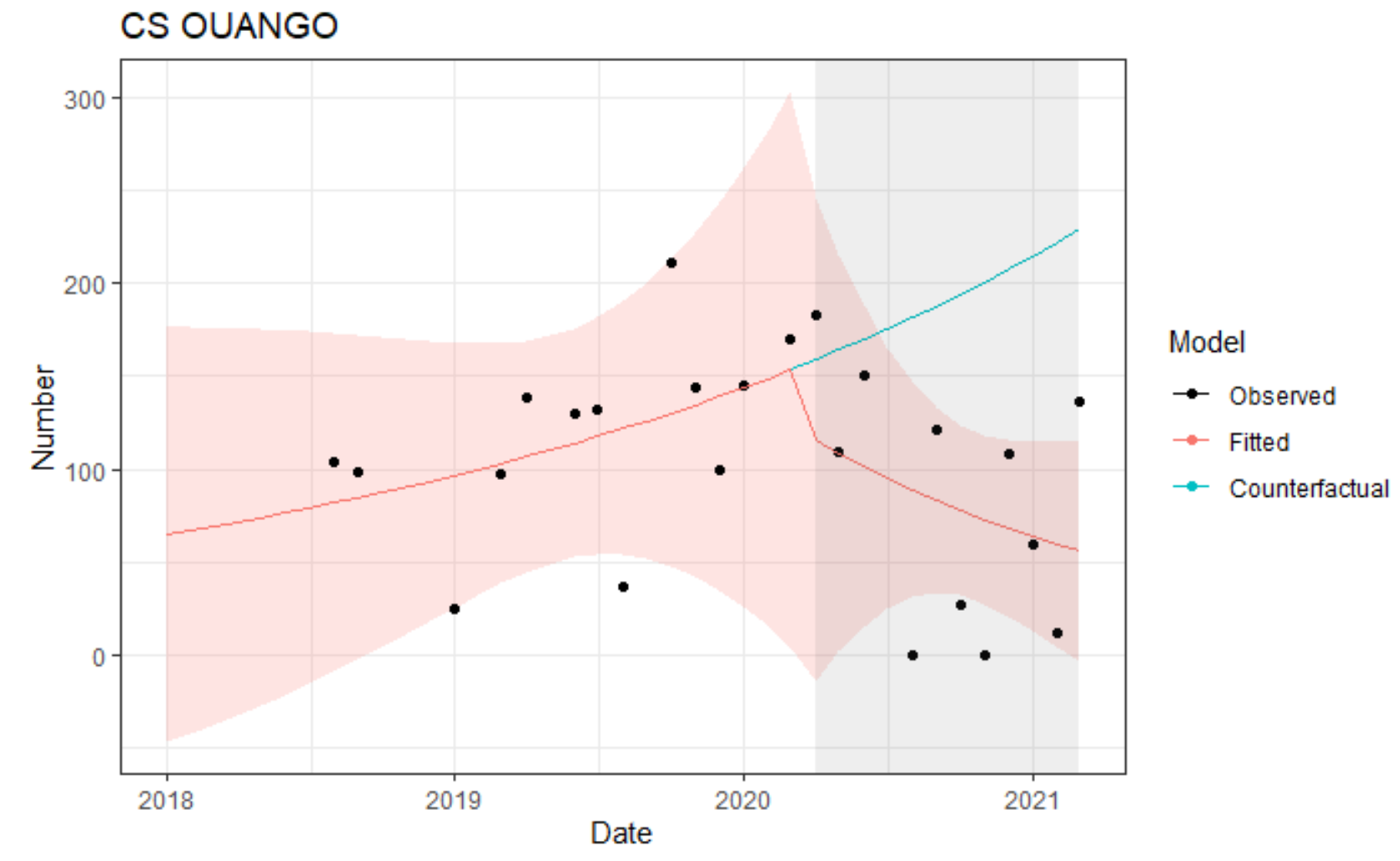 | 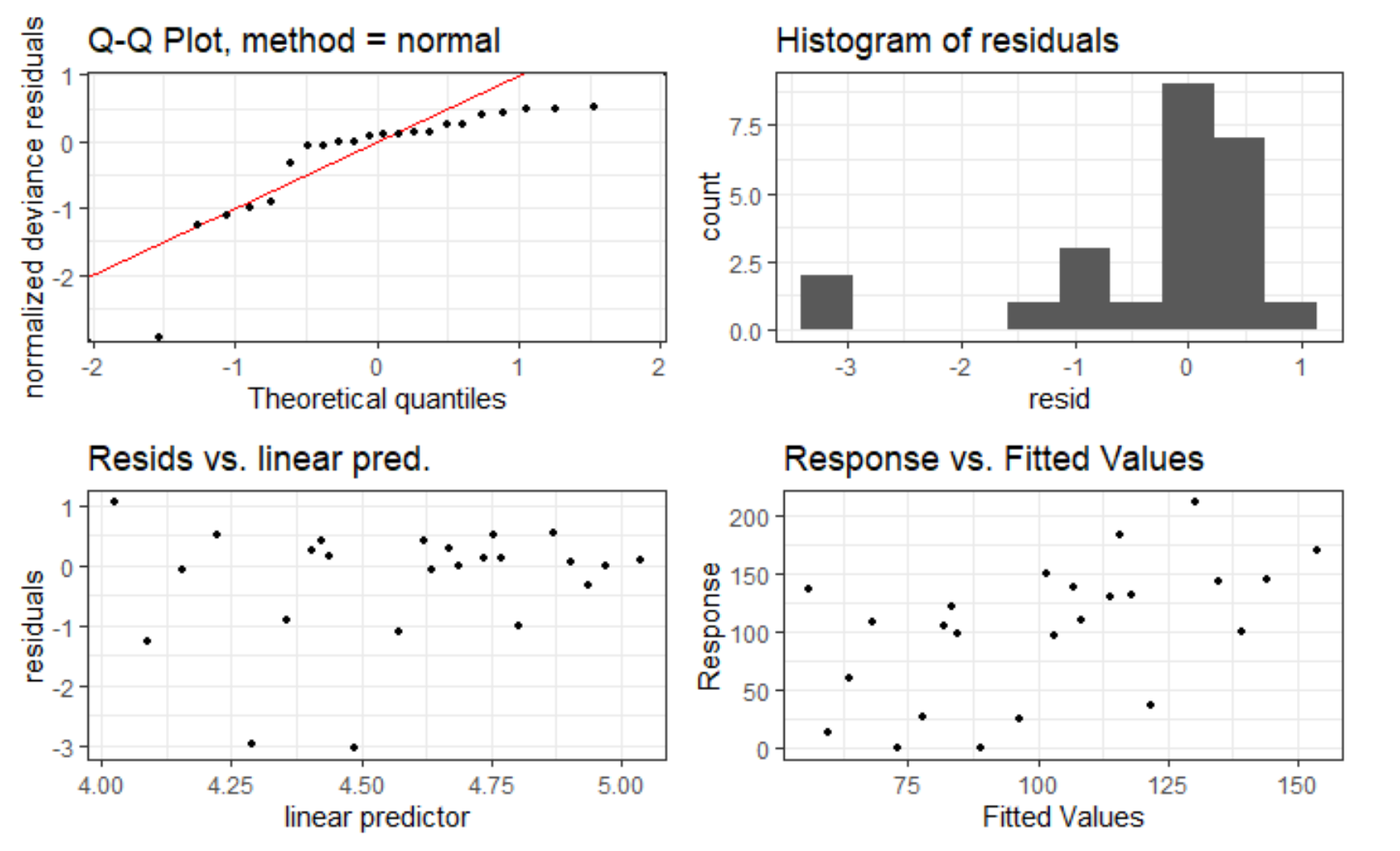 |
| CS Urbain Lakouanga | 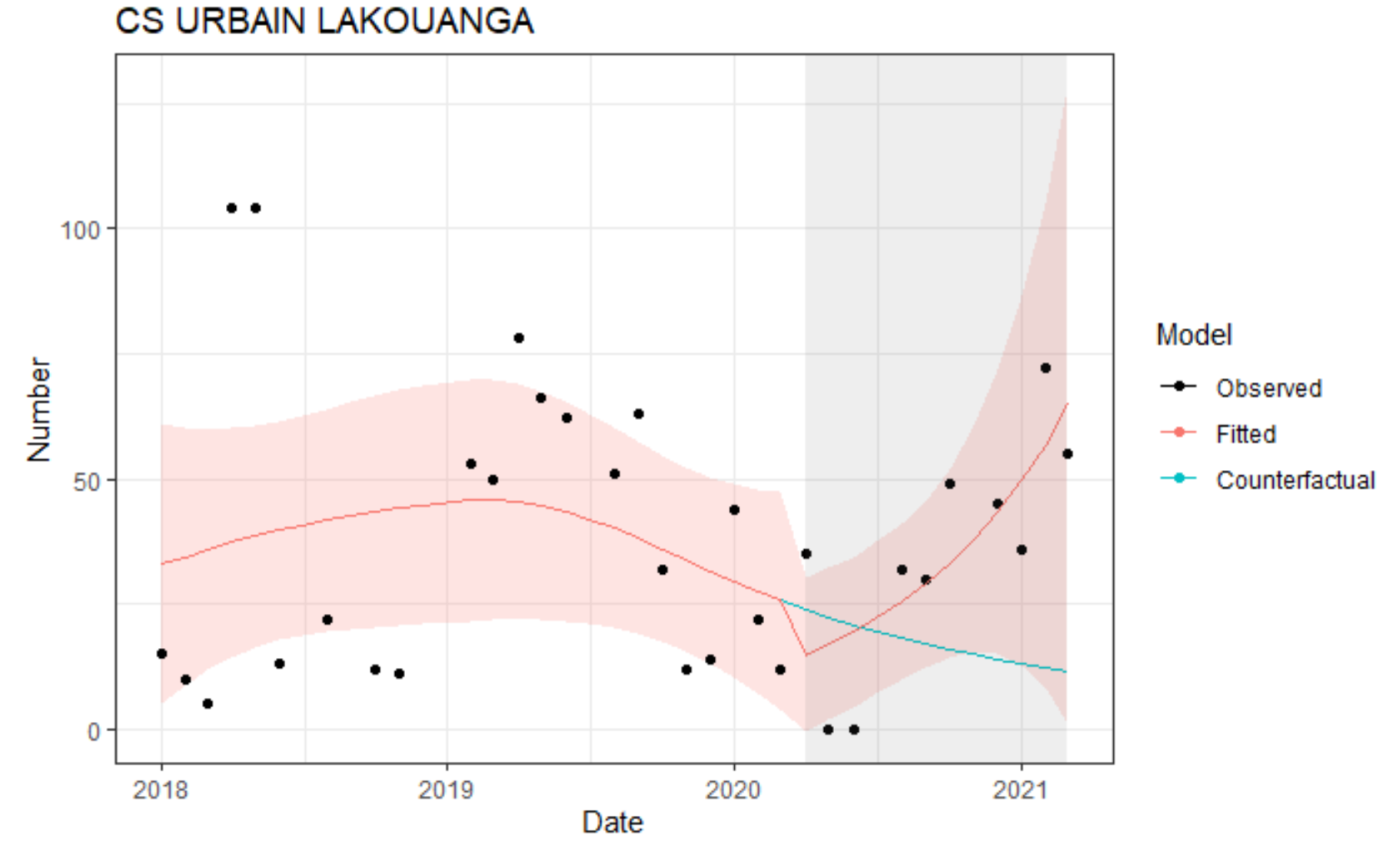 | 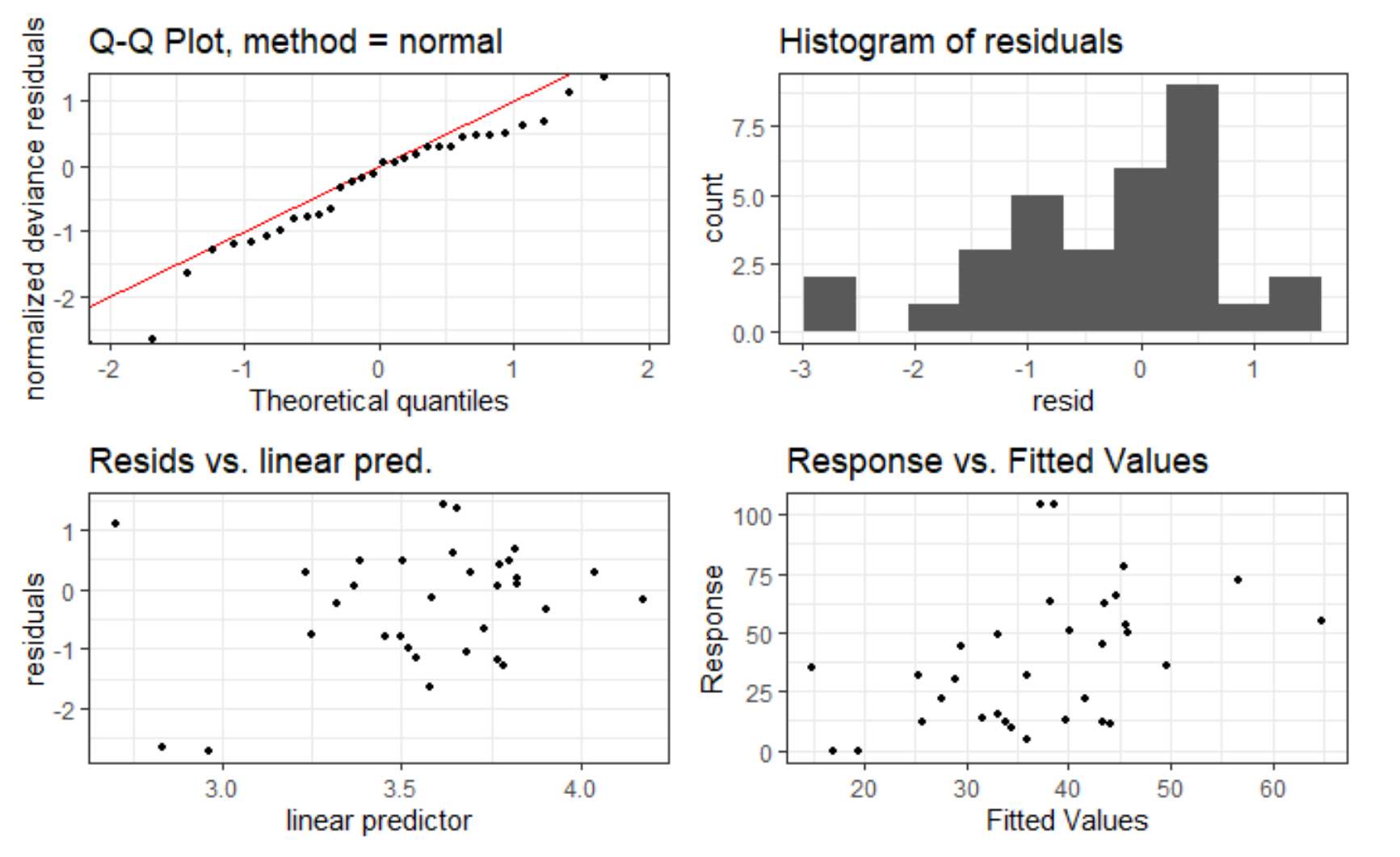 |
| CS Urbain Yapele | 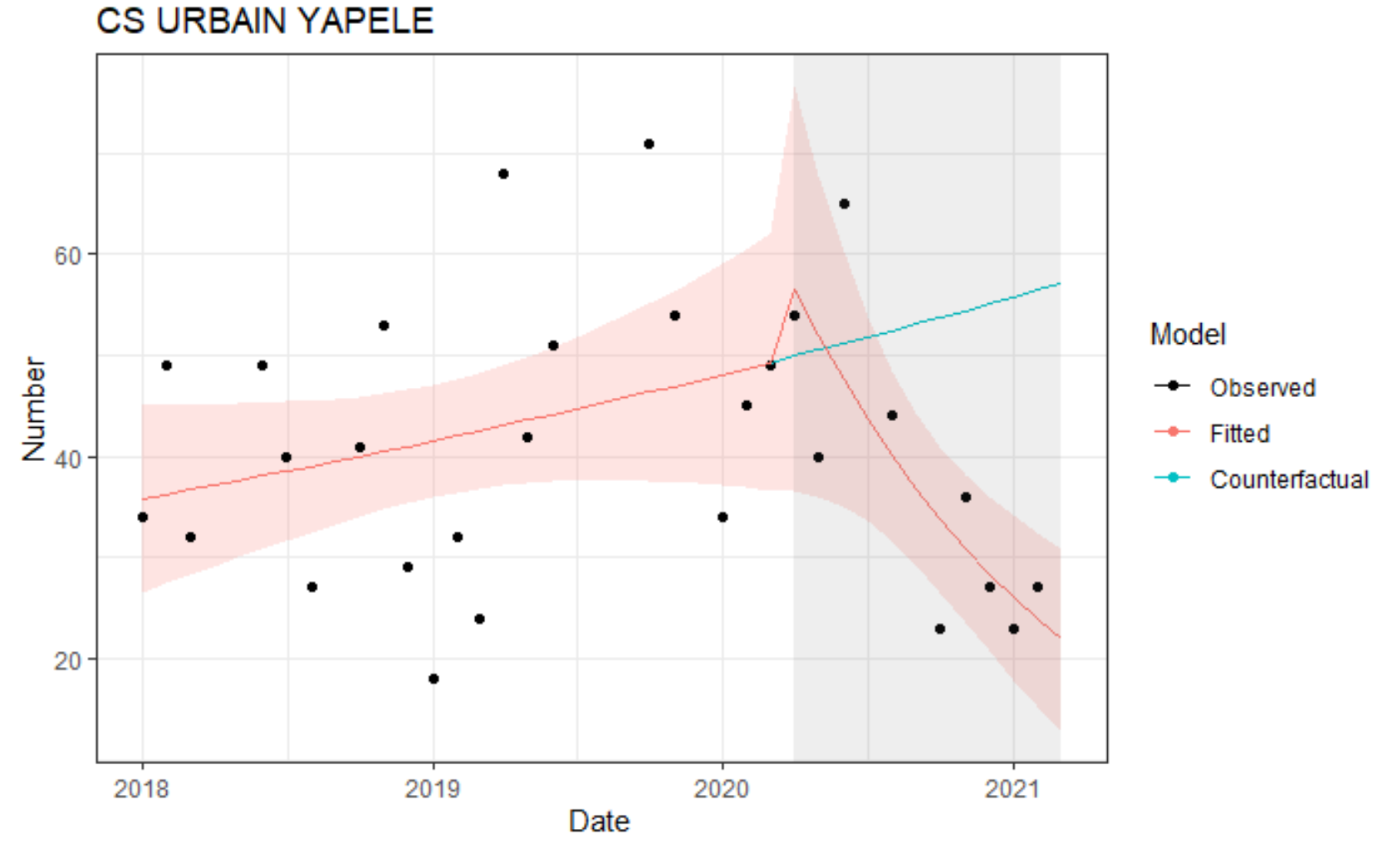 | 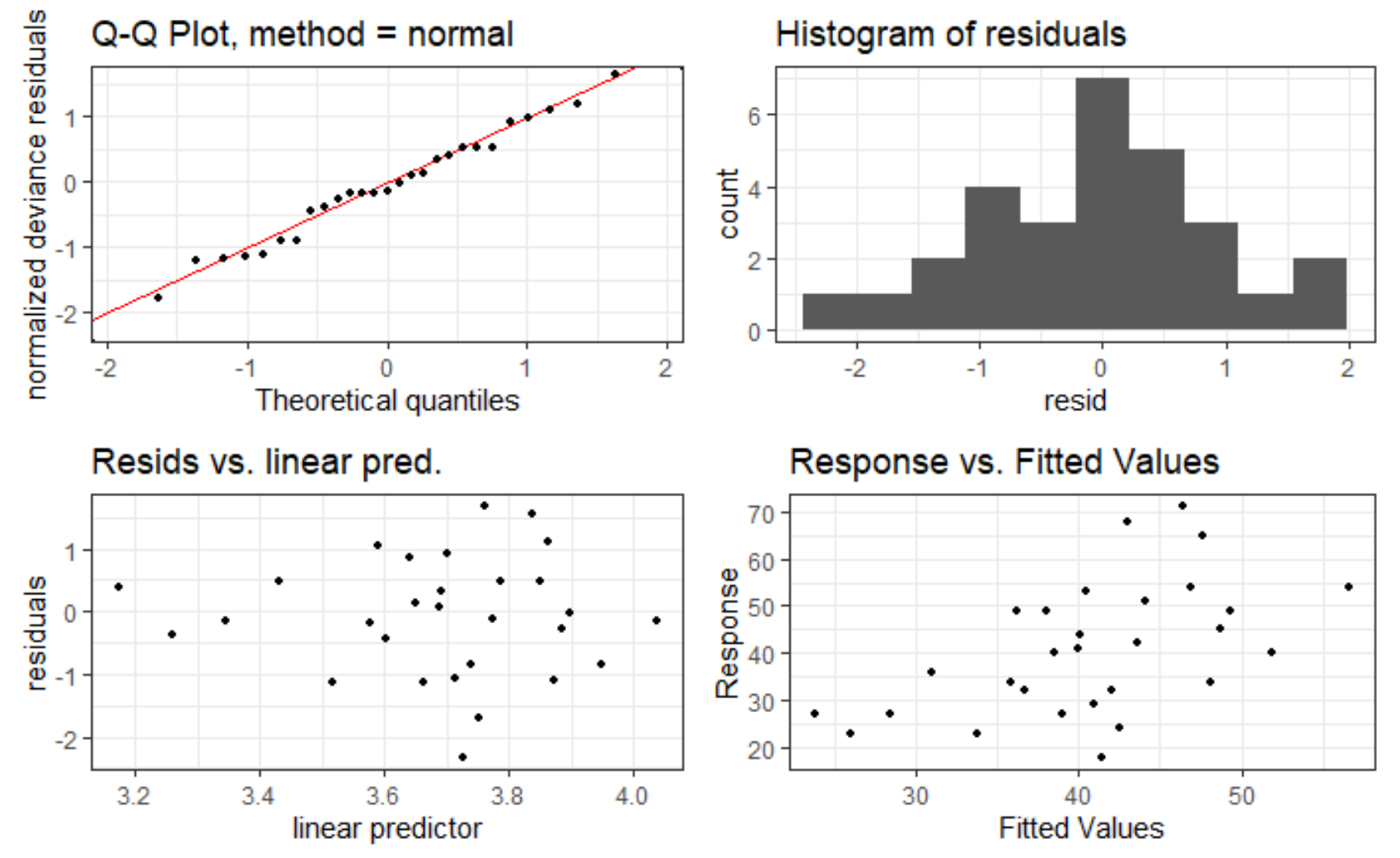 |
| CSU Ngaragba | 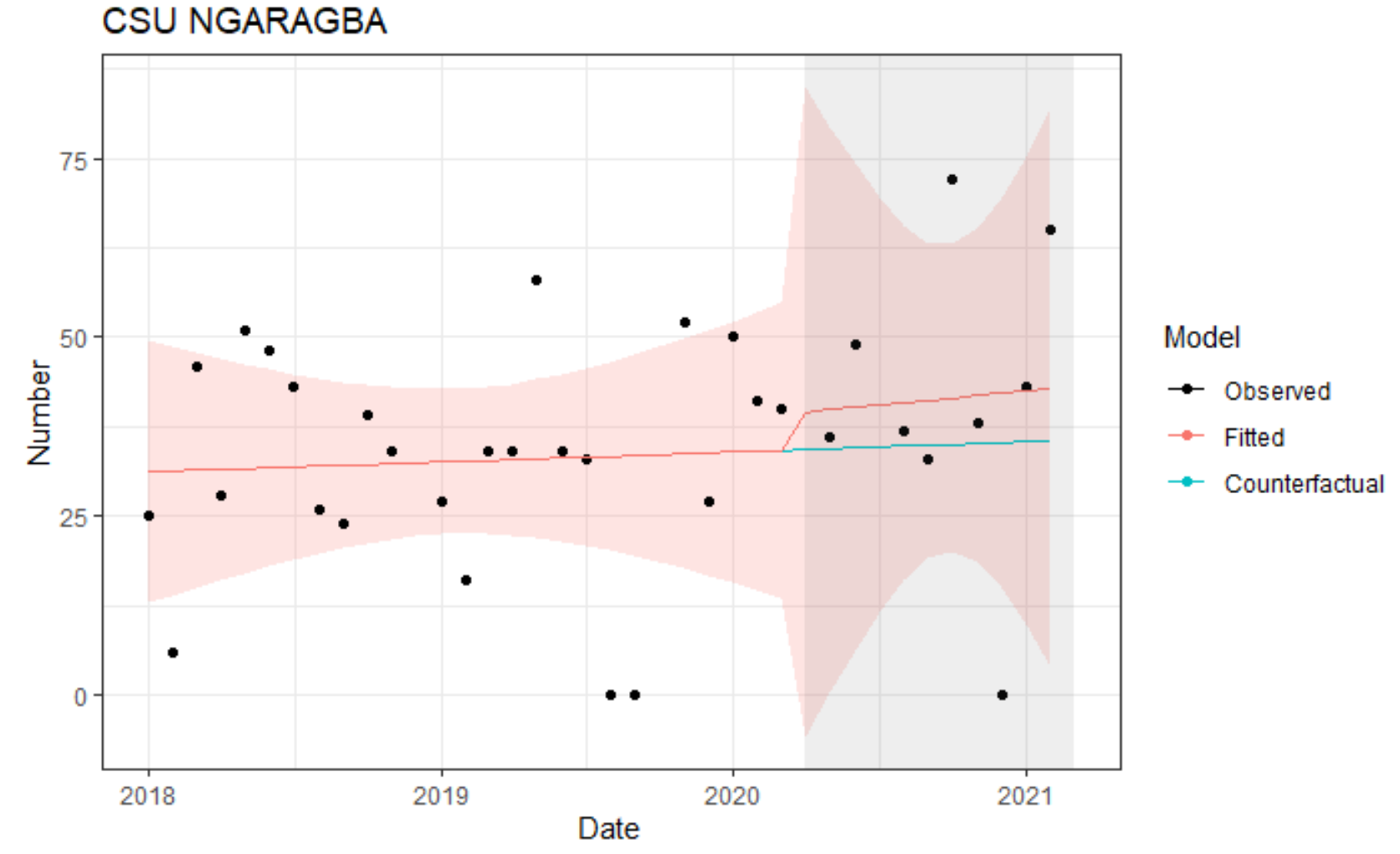 | 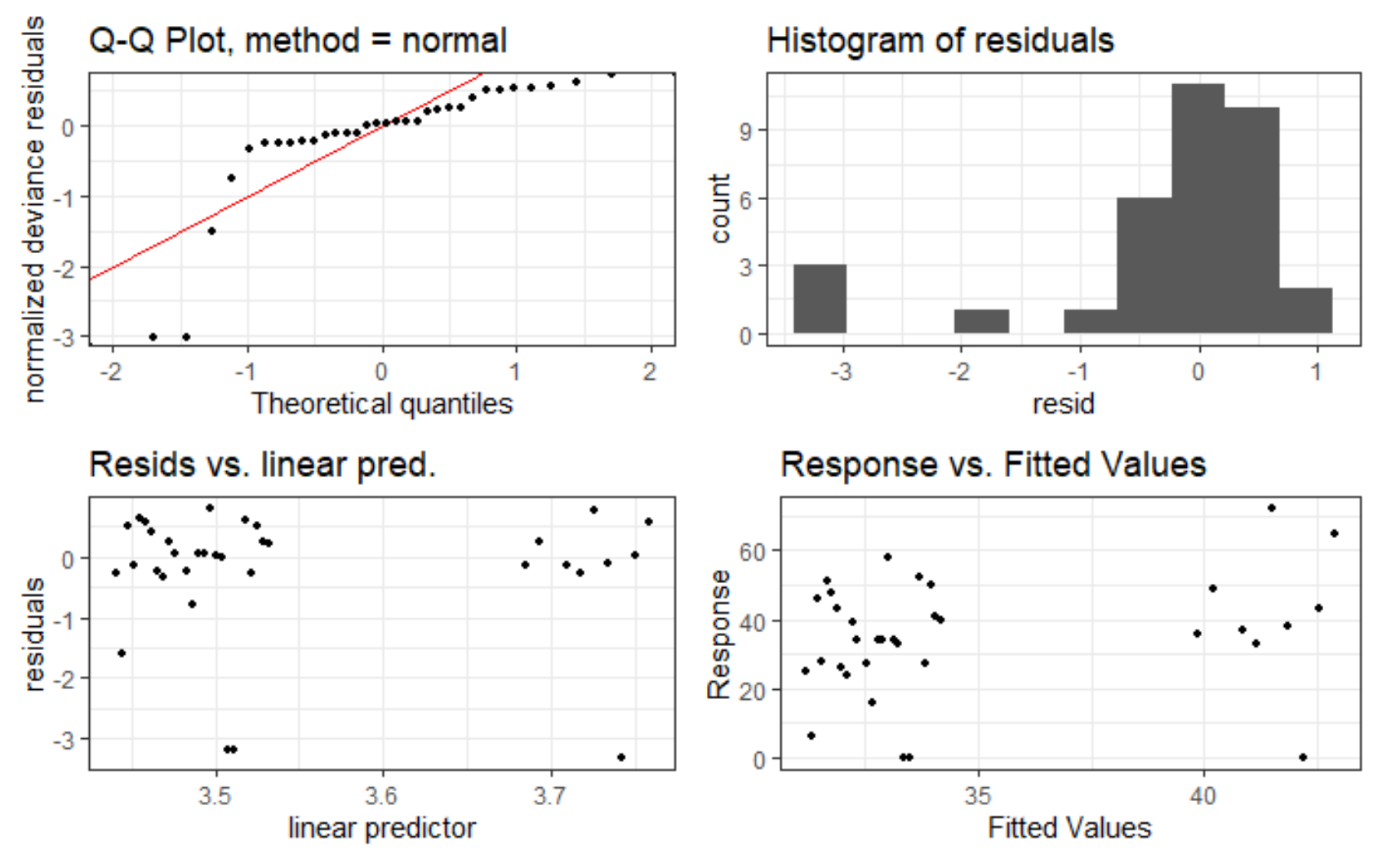 |

#### Bangui 2

Note: excluded CS URBAIN MAMDOU MBAIKI because of unrealistic counterfactual


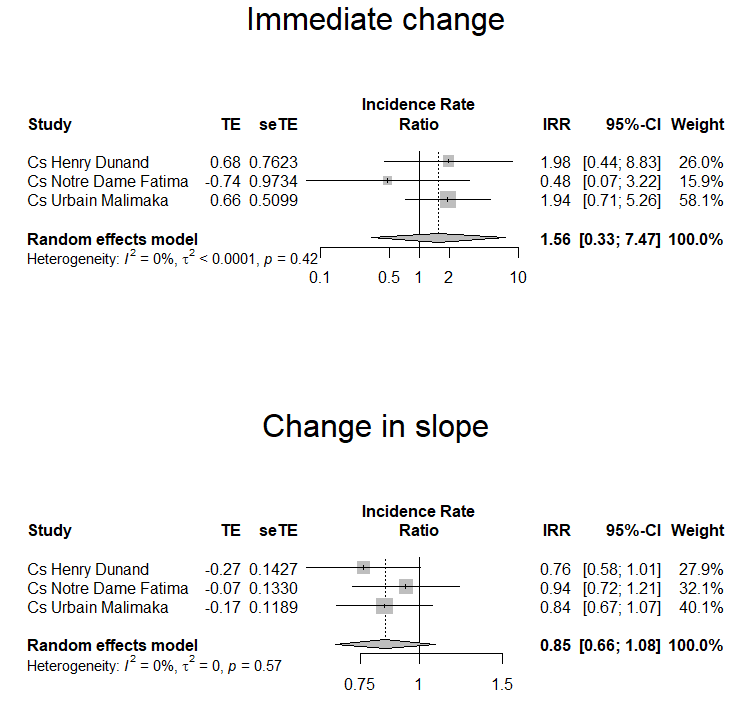


Model fit and residuals

| Facility | Model fit | Residuals |
| --- | --- | --- |
| CS Henry Dunand | 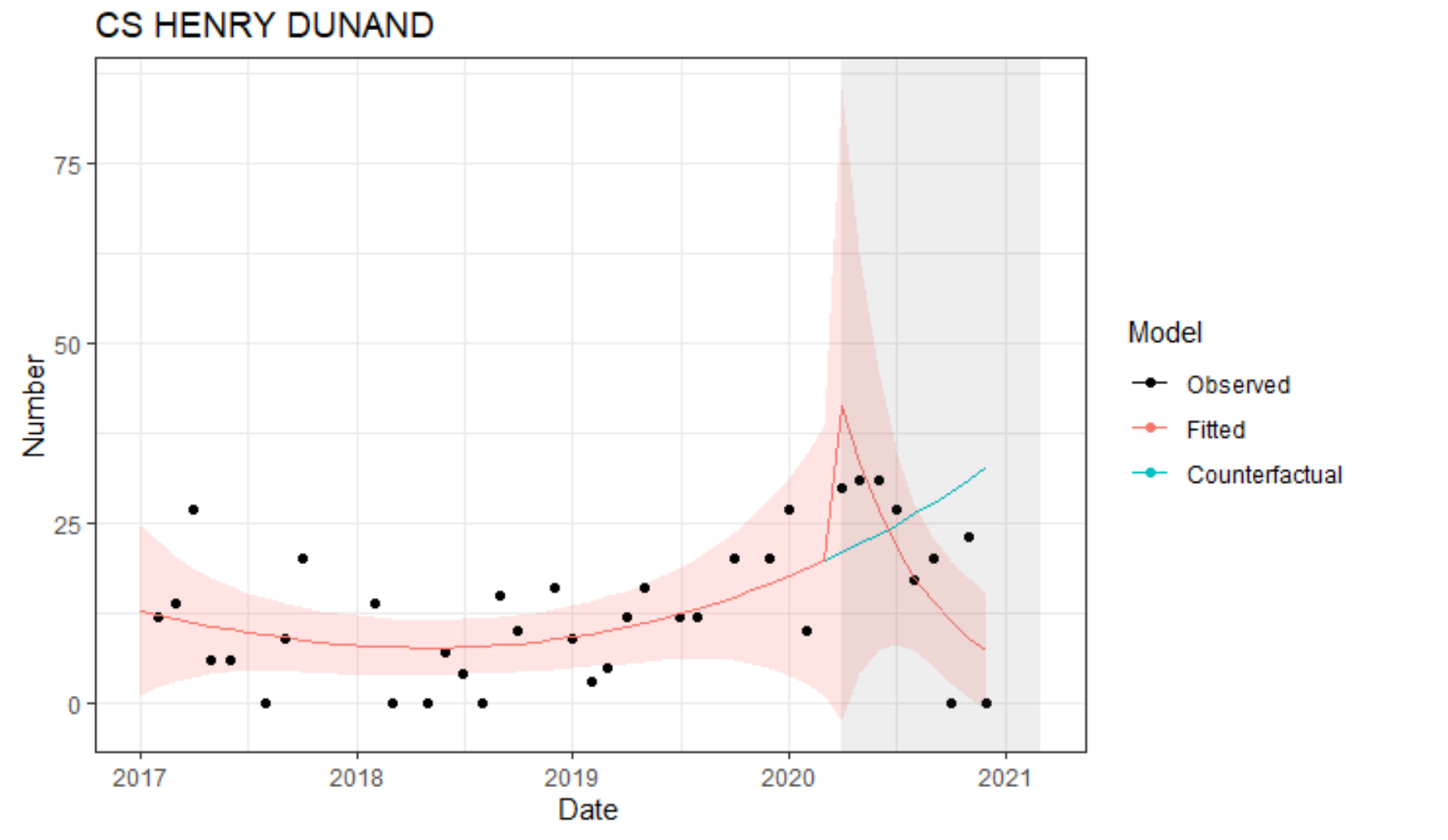 | 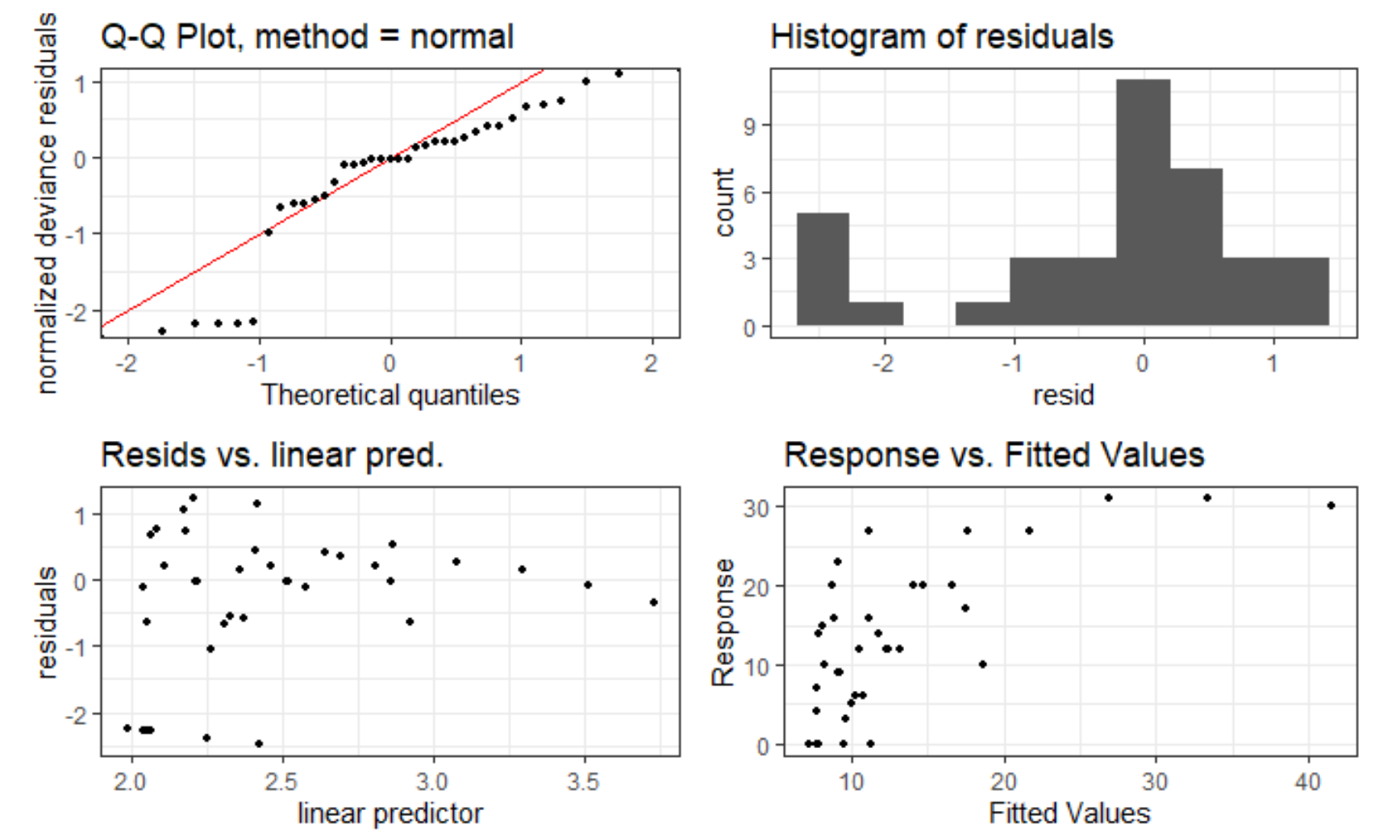 |
| CS Notre Dame Fatima | 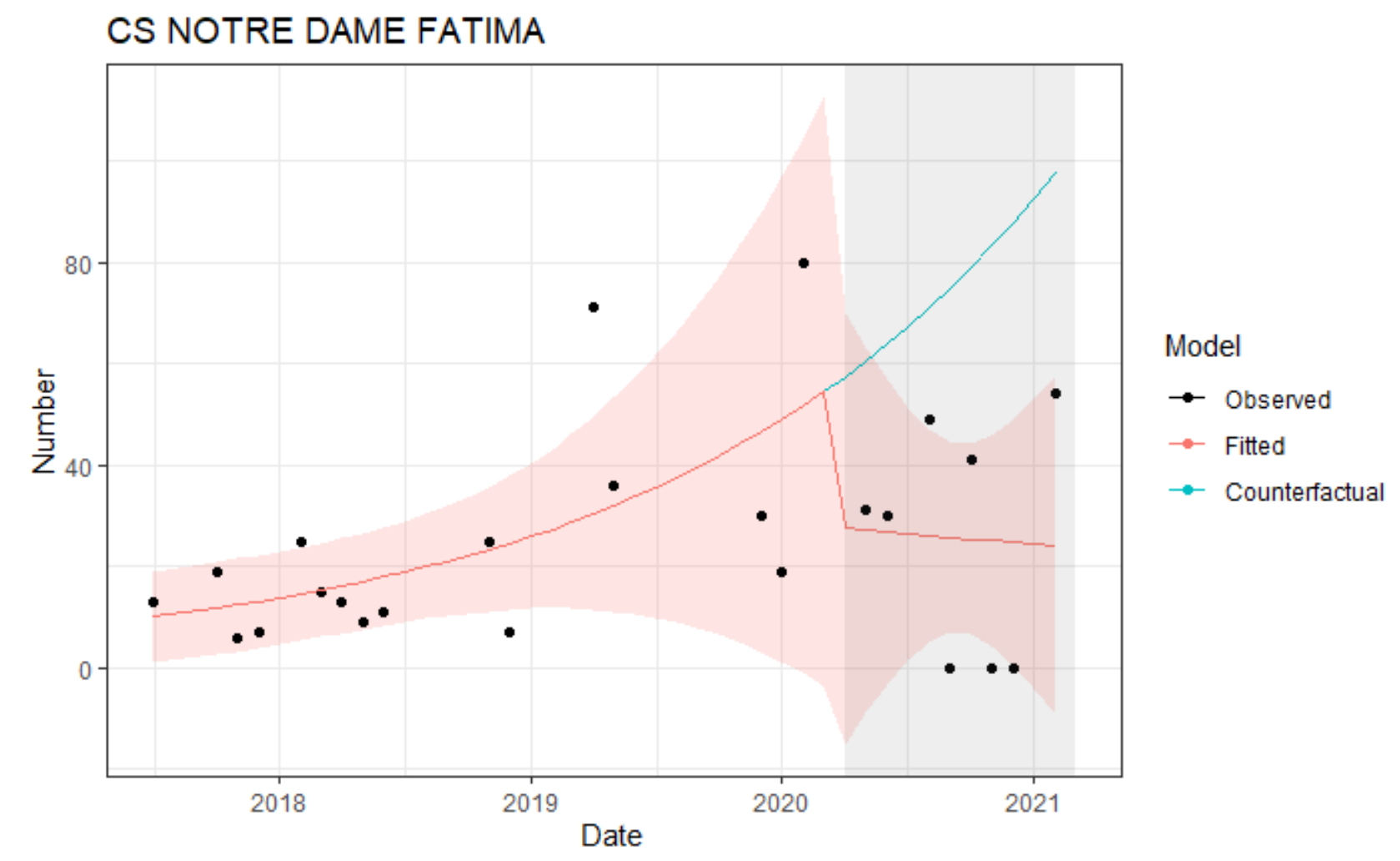 | 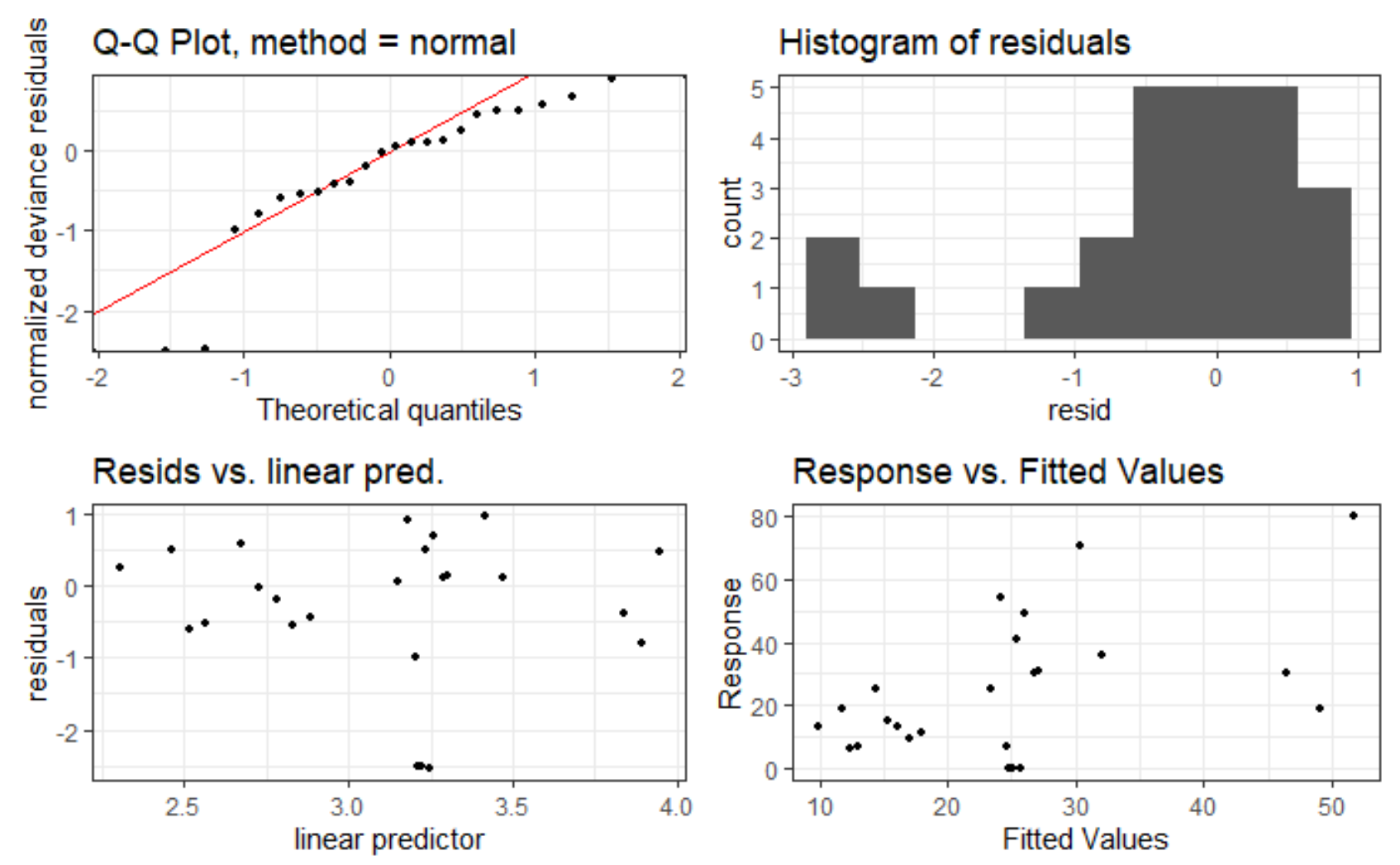 |
| CS Urbain Malimaka | 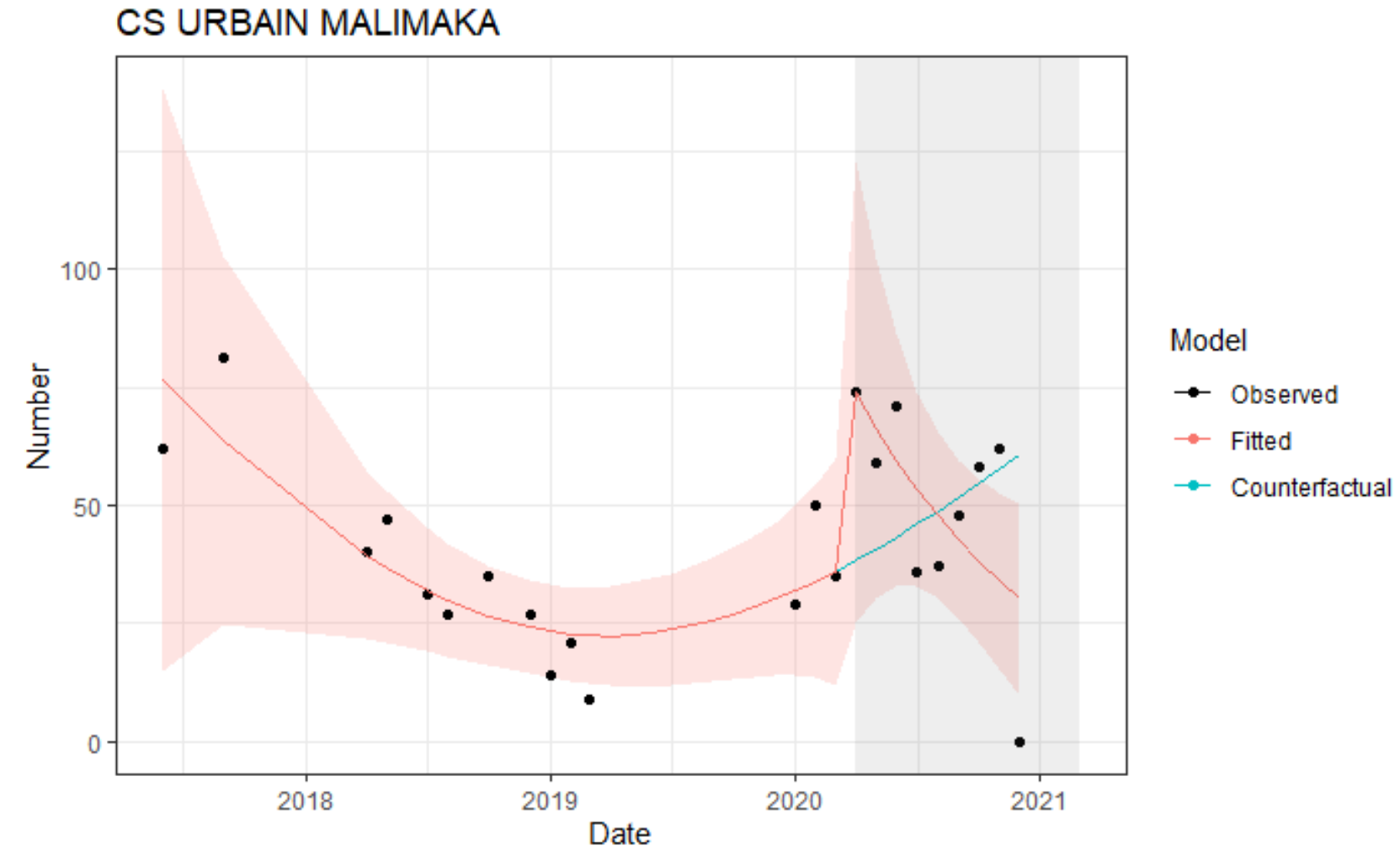 | 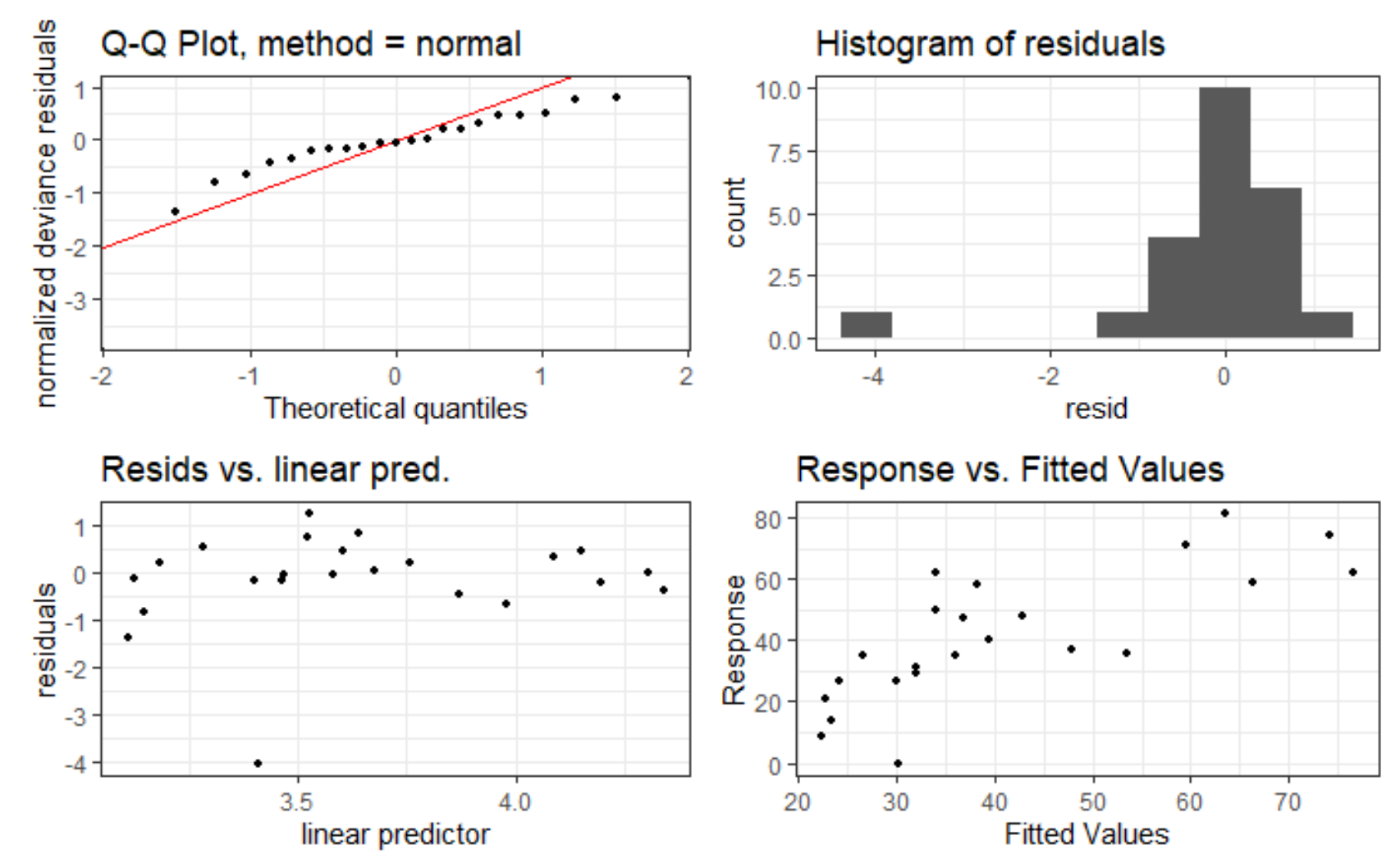 |

#### Bangui 3

Note: removed Tumba Yere because of unrealistic counterfactual


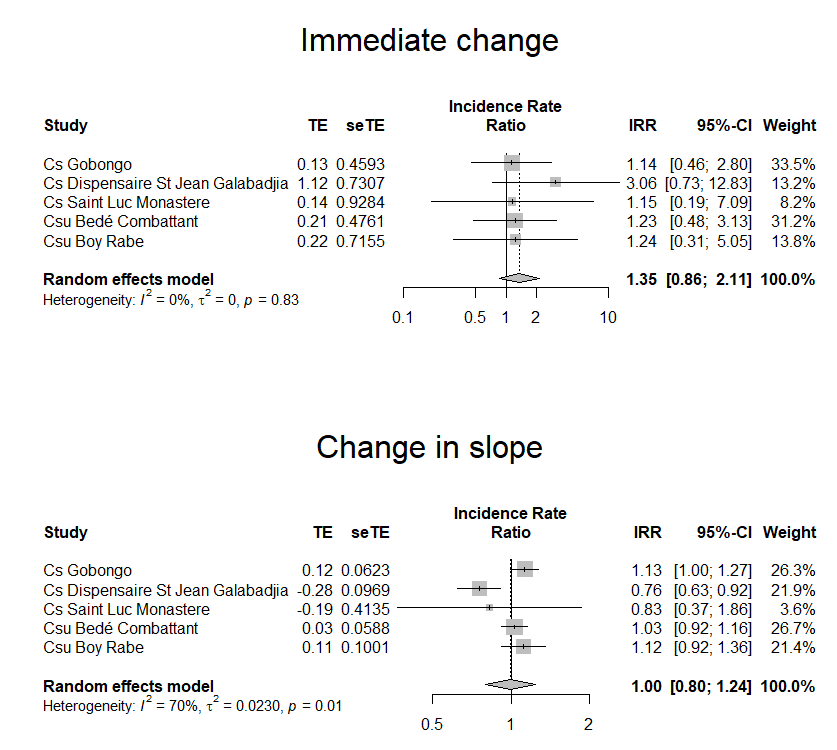


Model fit and residuals

| Facility | Model fit | Residuals |
| --- | --- | --- |
| CS Gobongo | 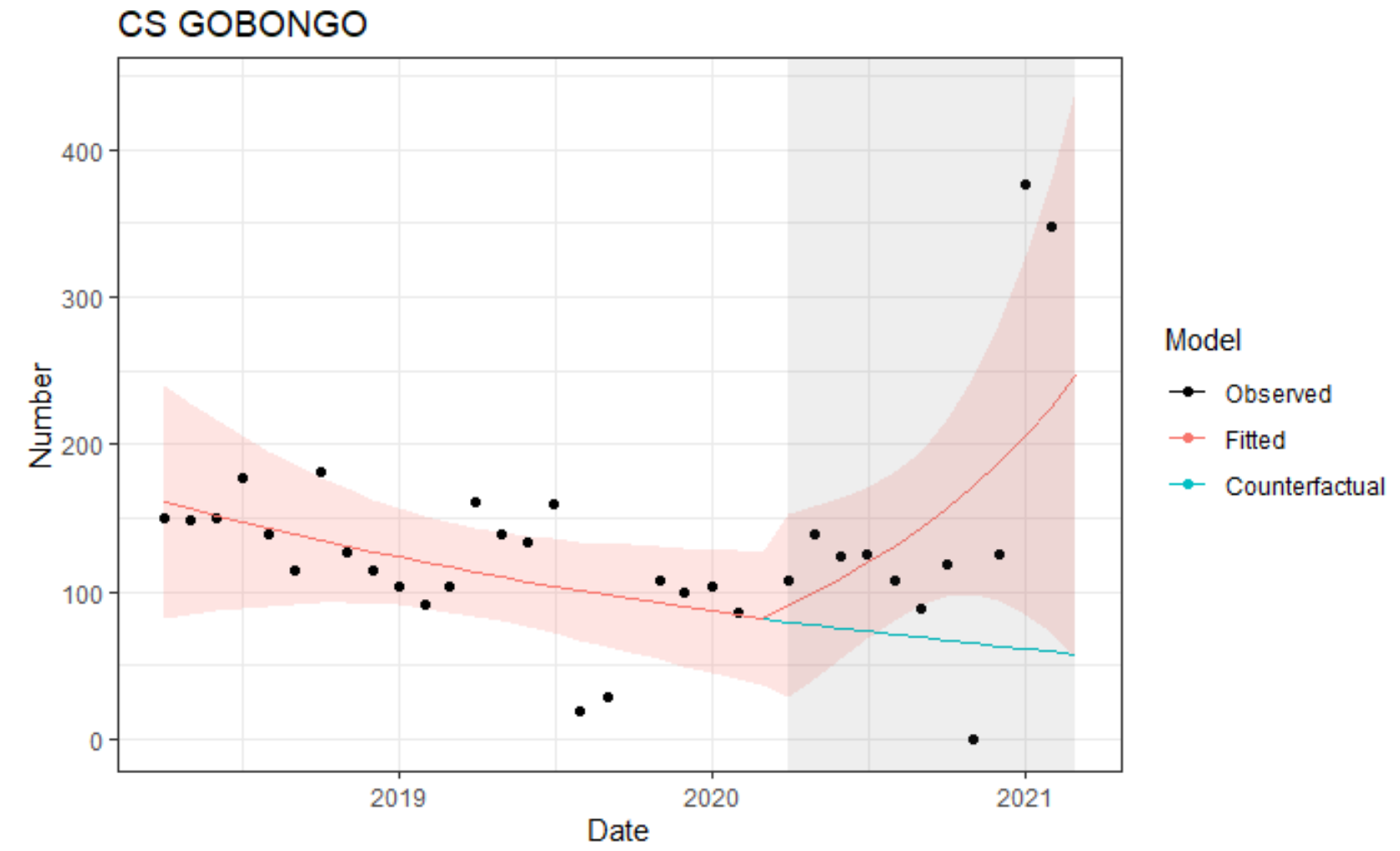 | 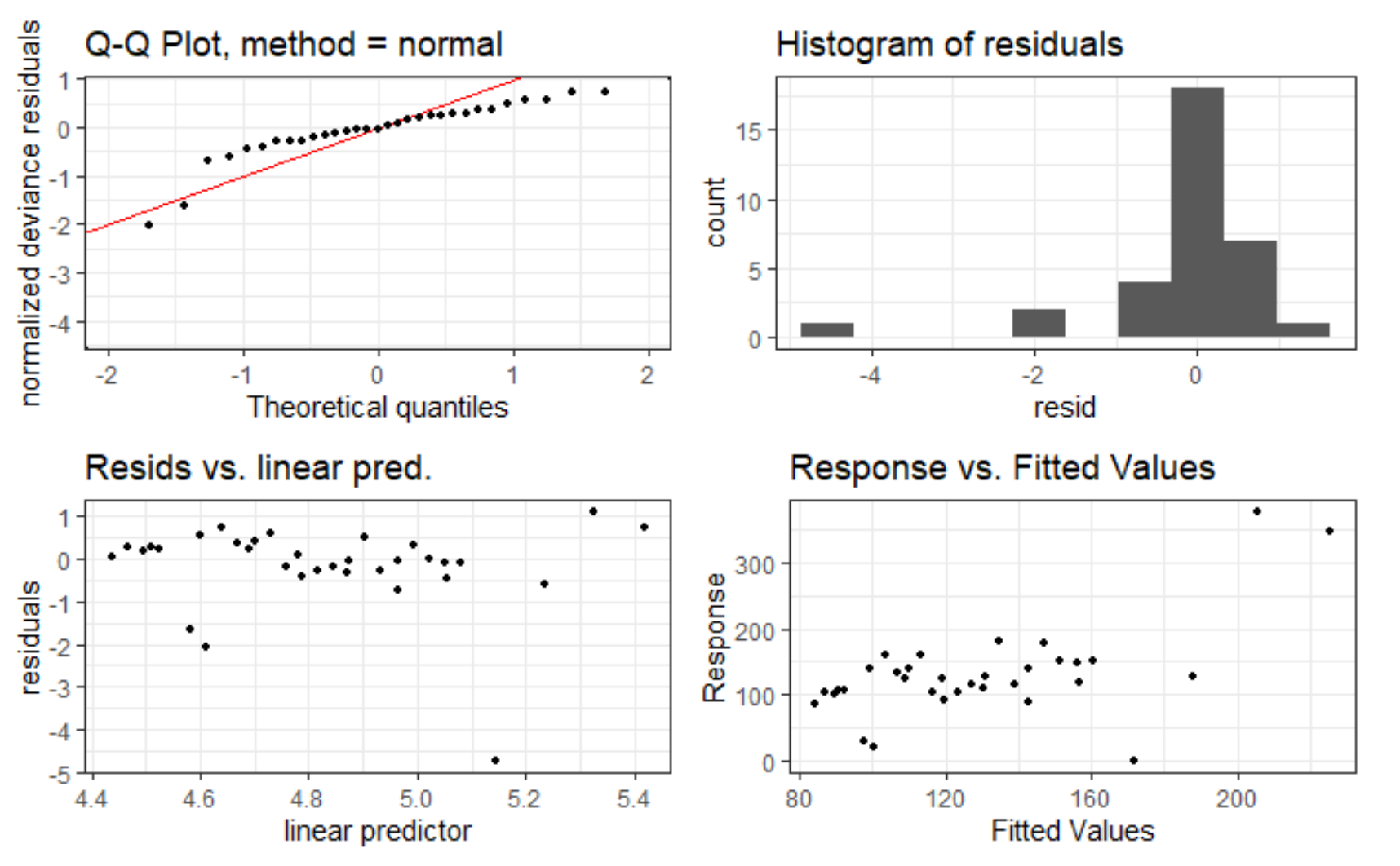 |
| CS Dispensaire St Jean Galabadjia | 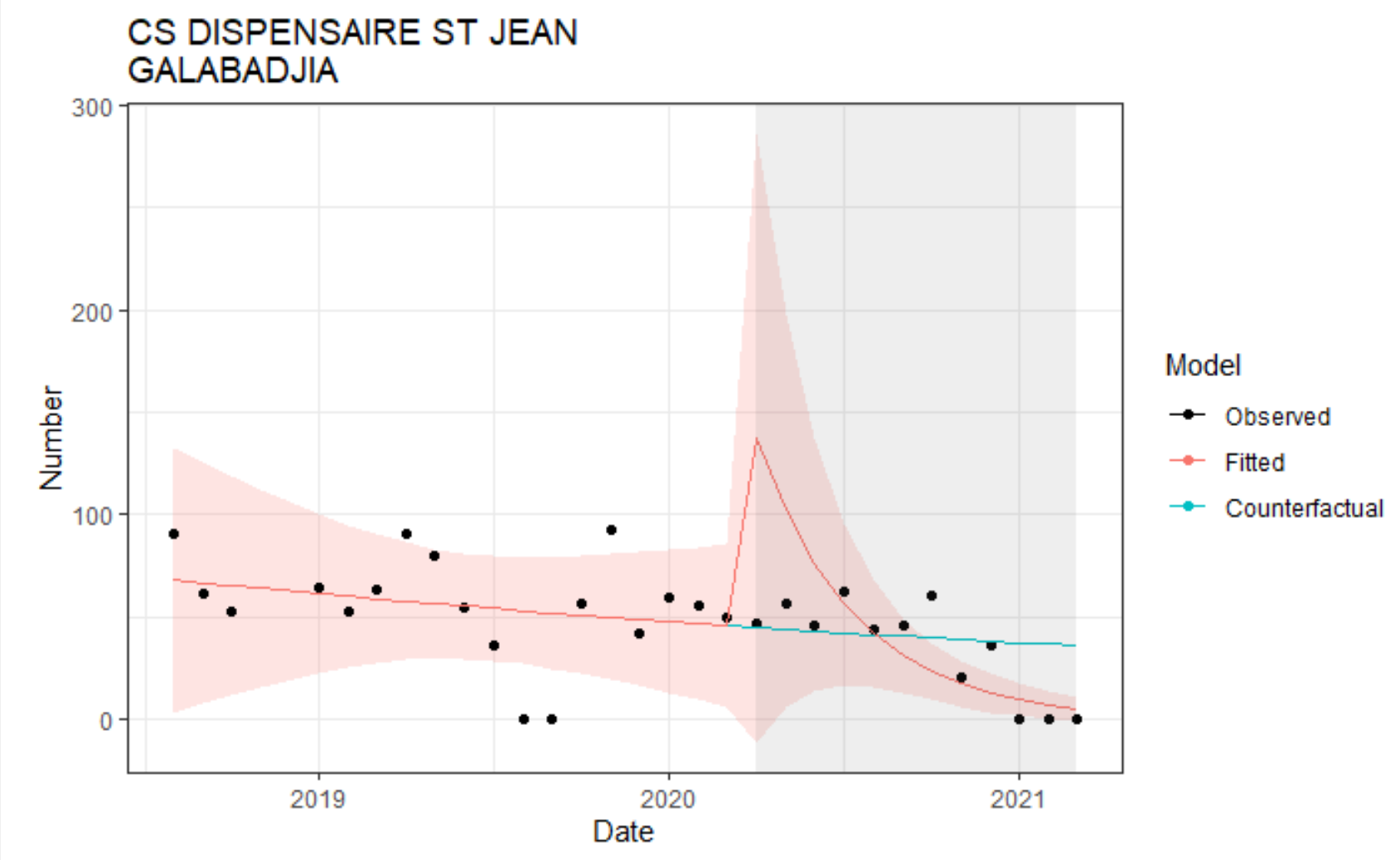 | 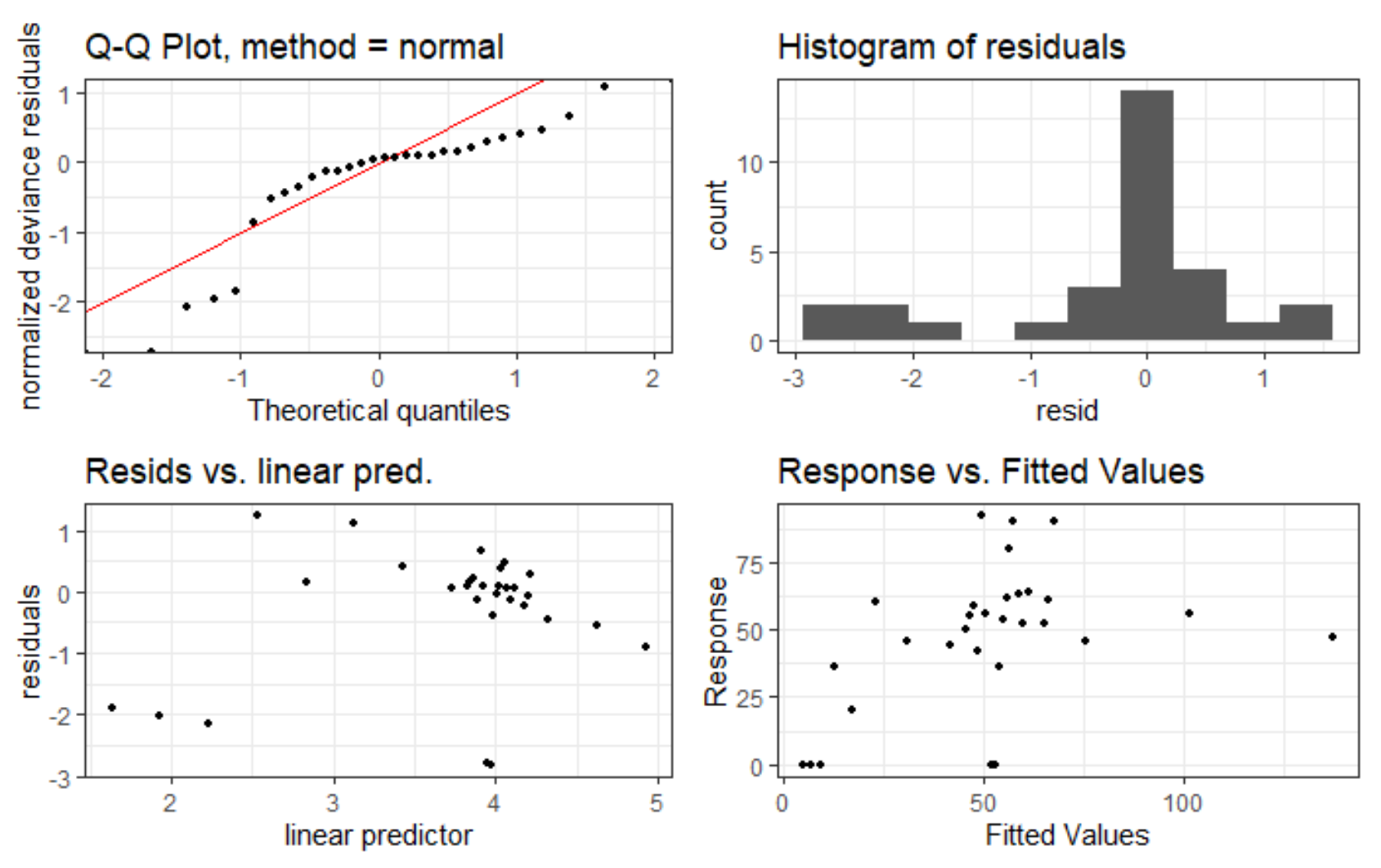 |
| CS Saint Luc Monastere | 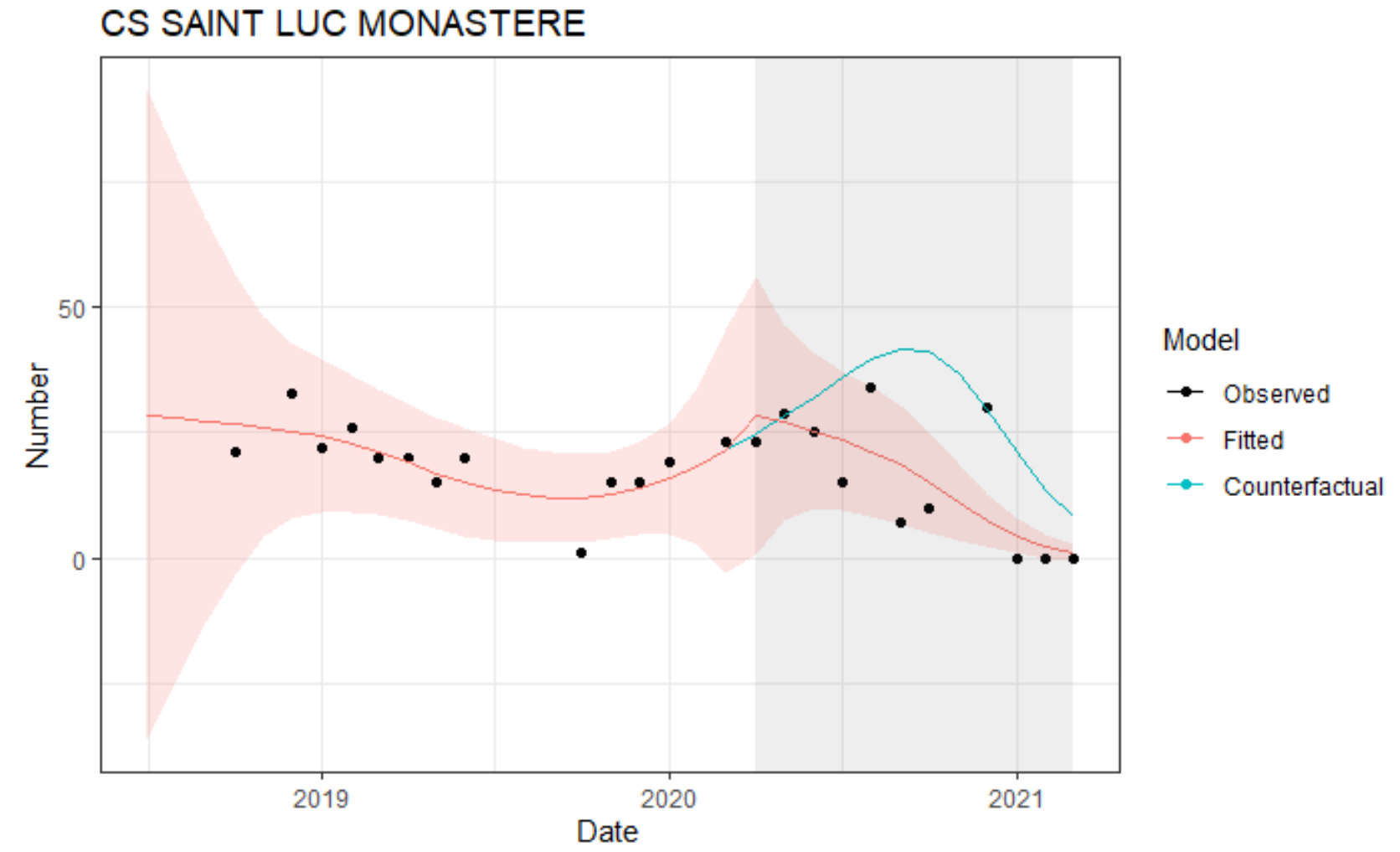 | 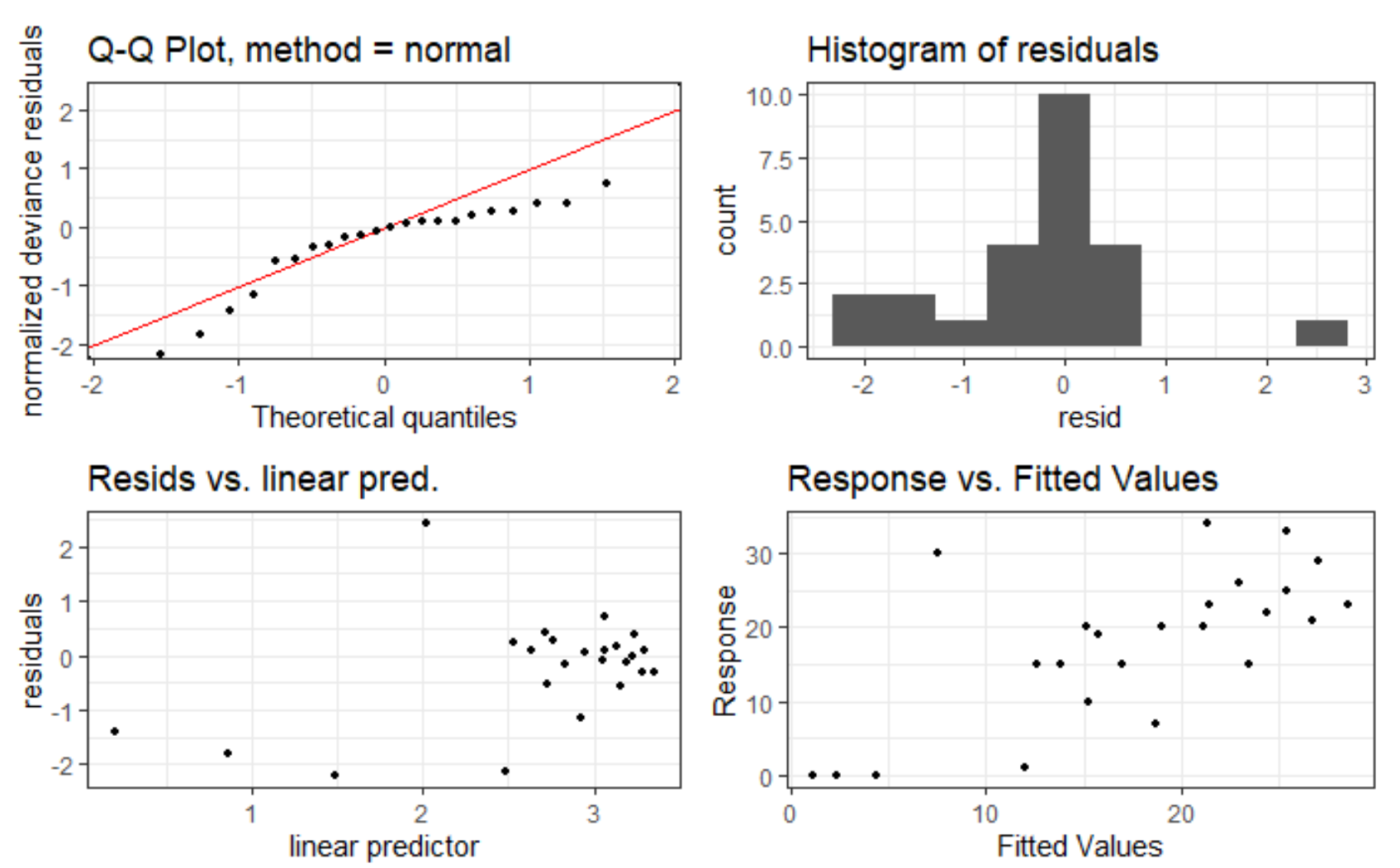 |
| CSU Bede Combattant | 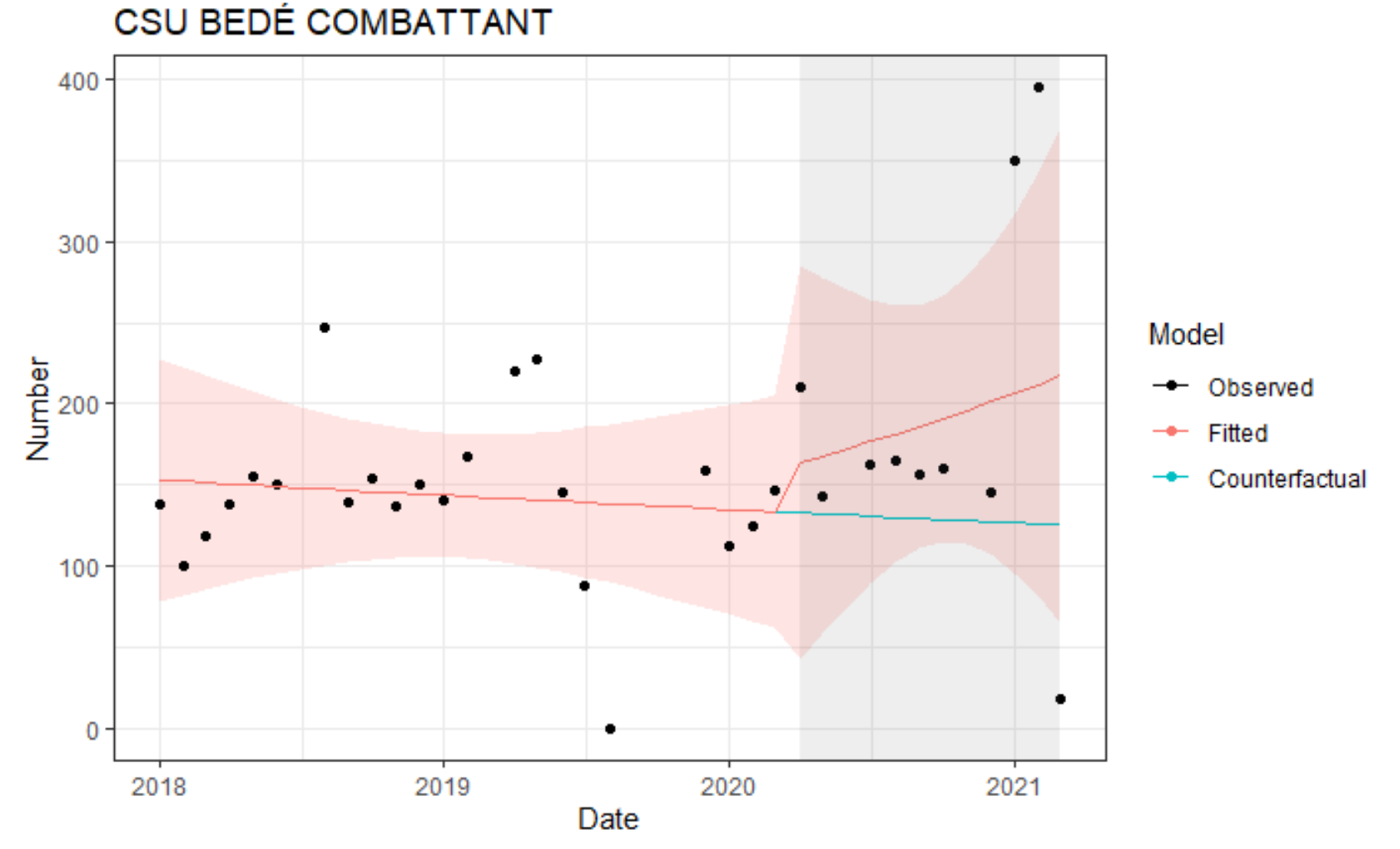 | 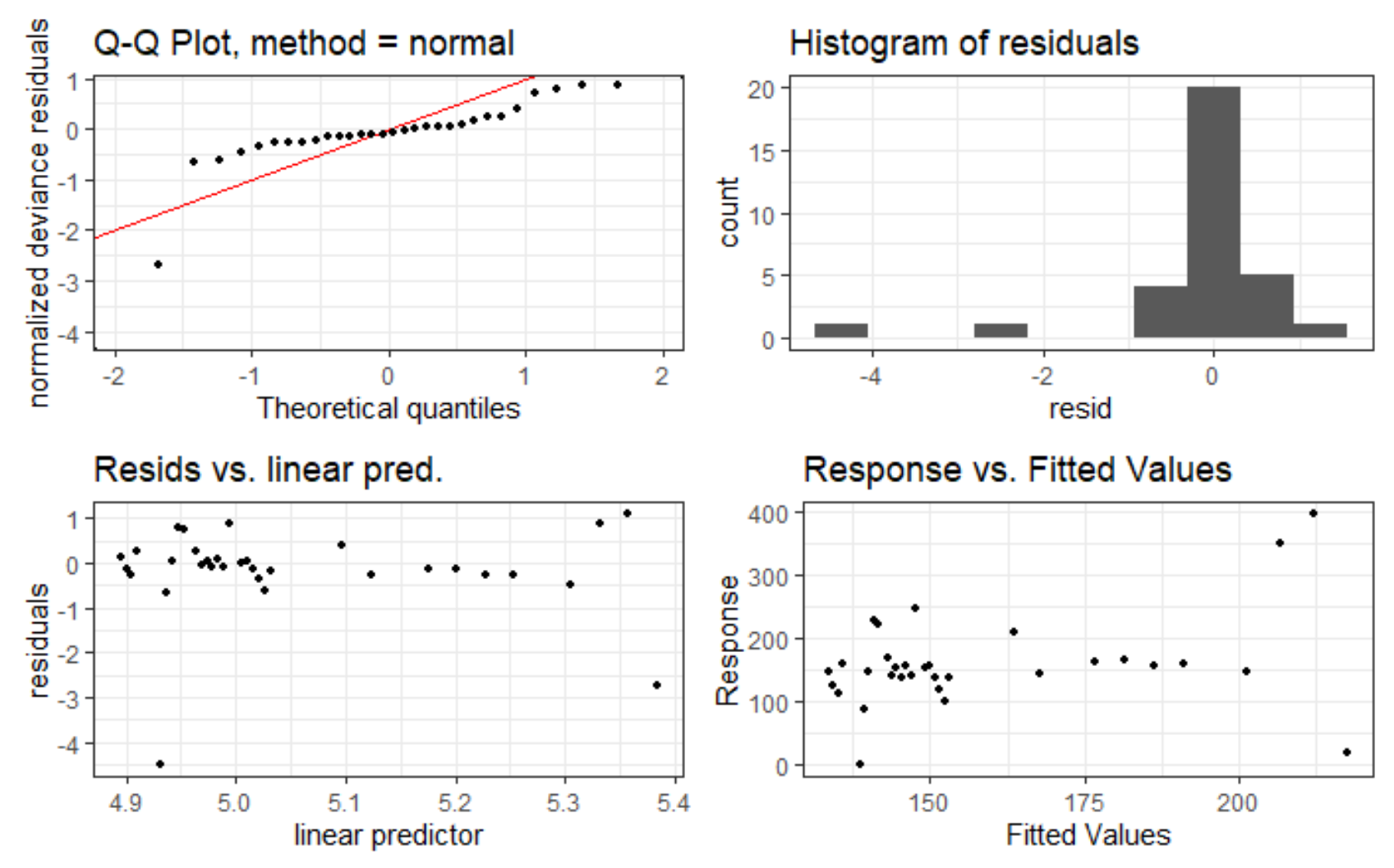 |
| CSU Boy Rabe | 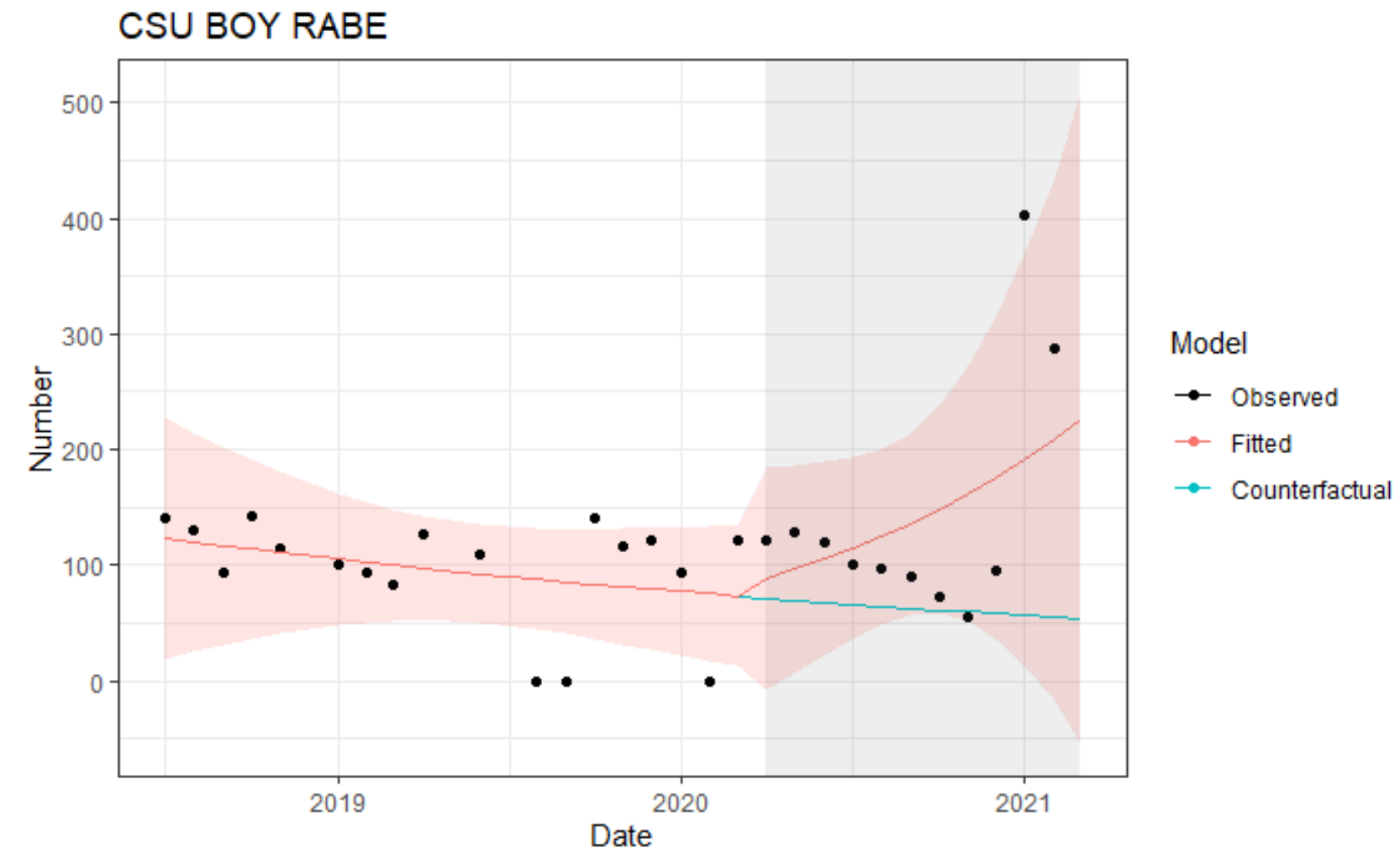 | 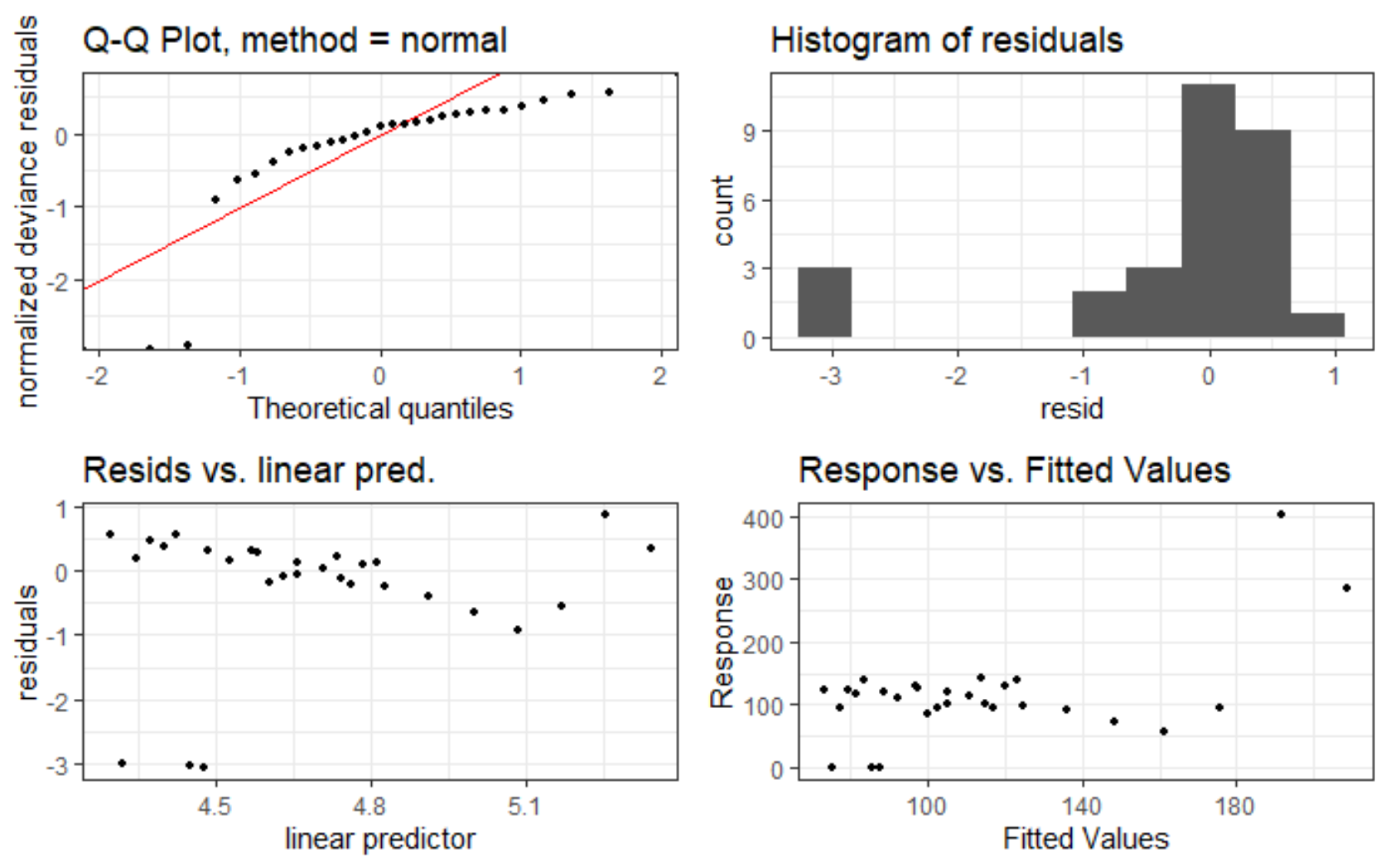 |

#### Begoua


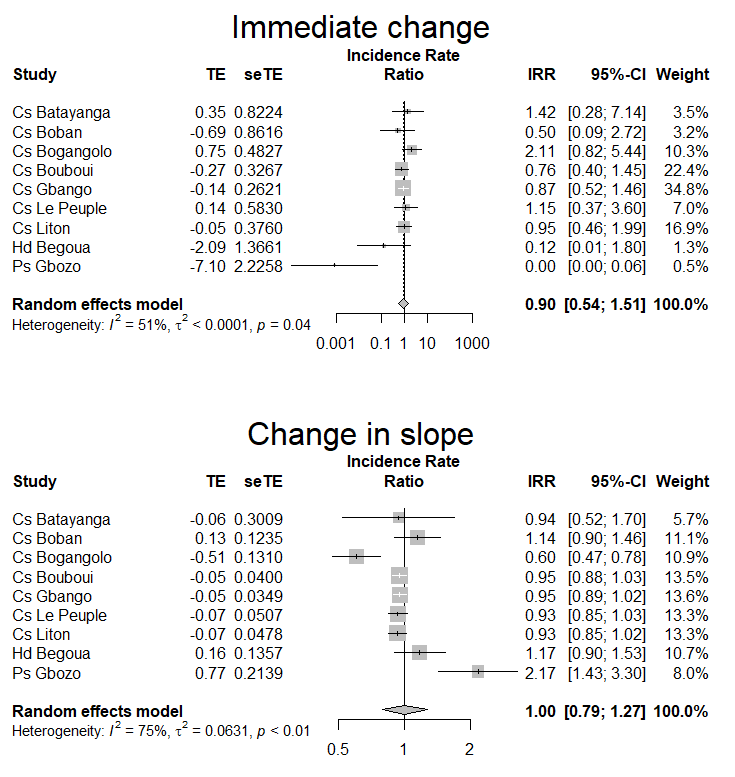


Model fit and residuals

| Facility | Model fit | Residuals |
| --- | --- | --- |
| CS Batayanga | 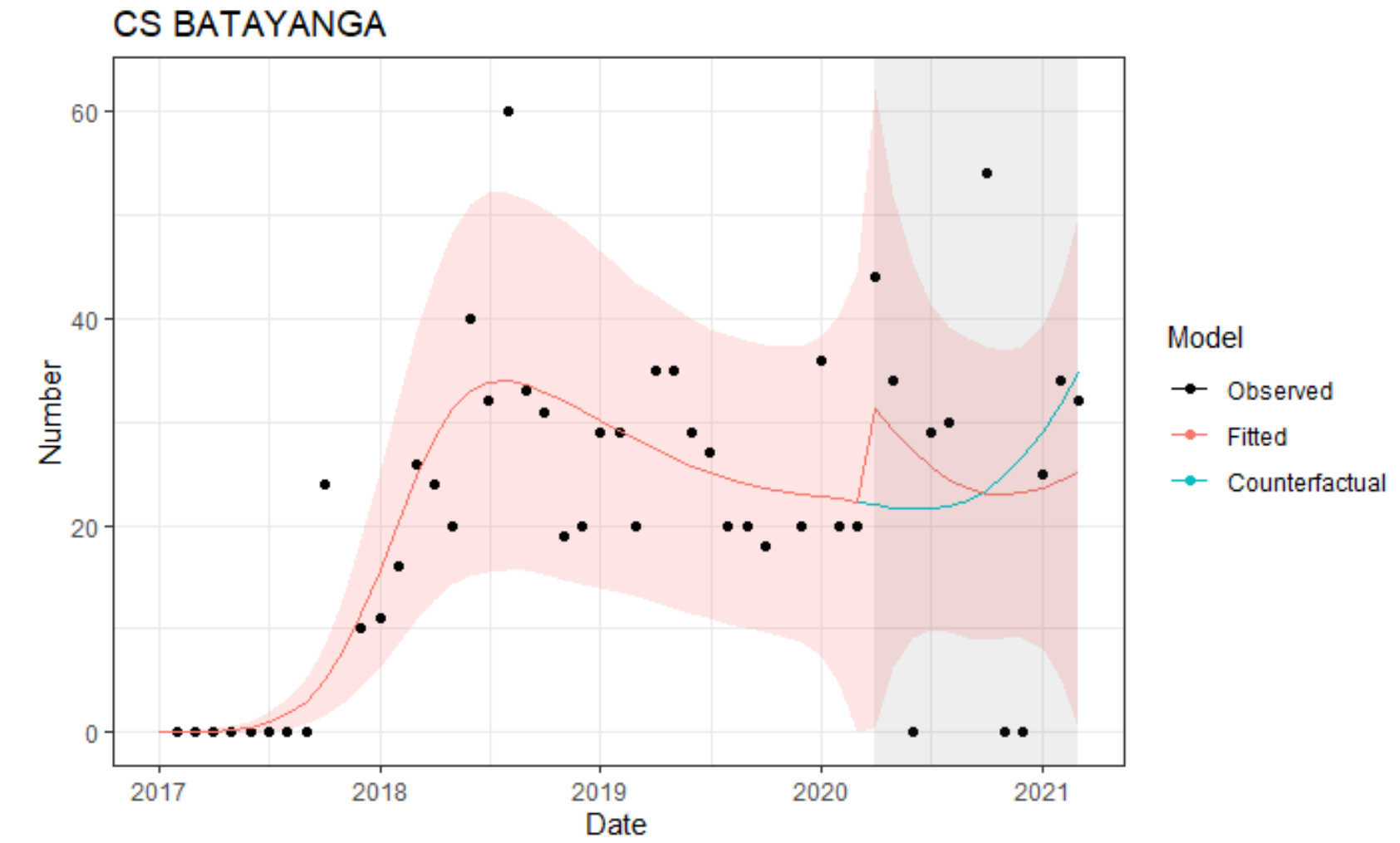 | 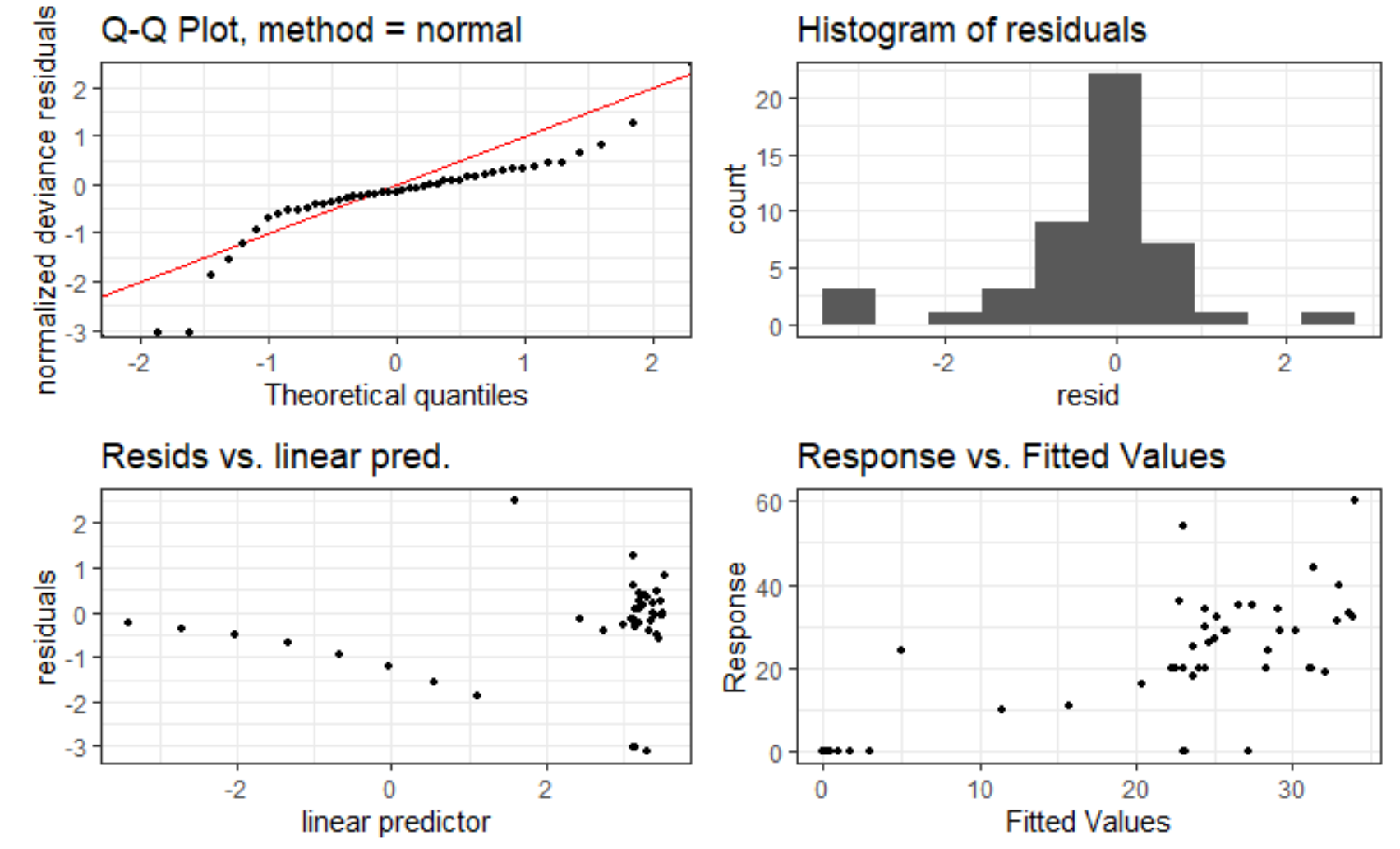 |
| CS Boban | 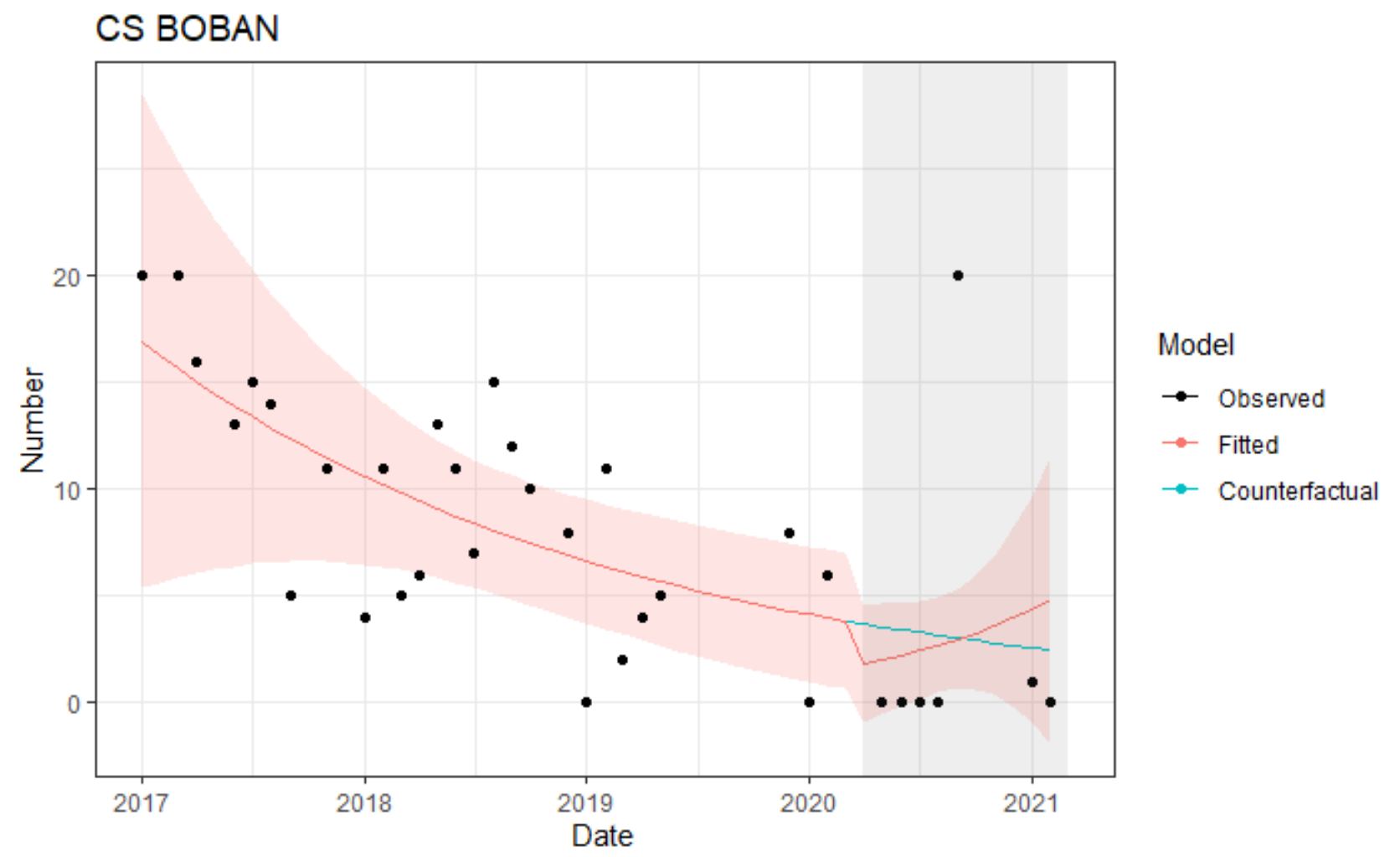 | 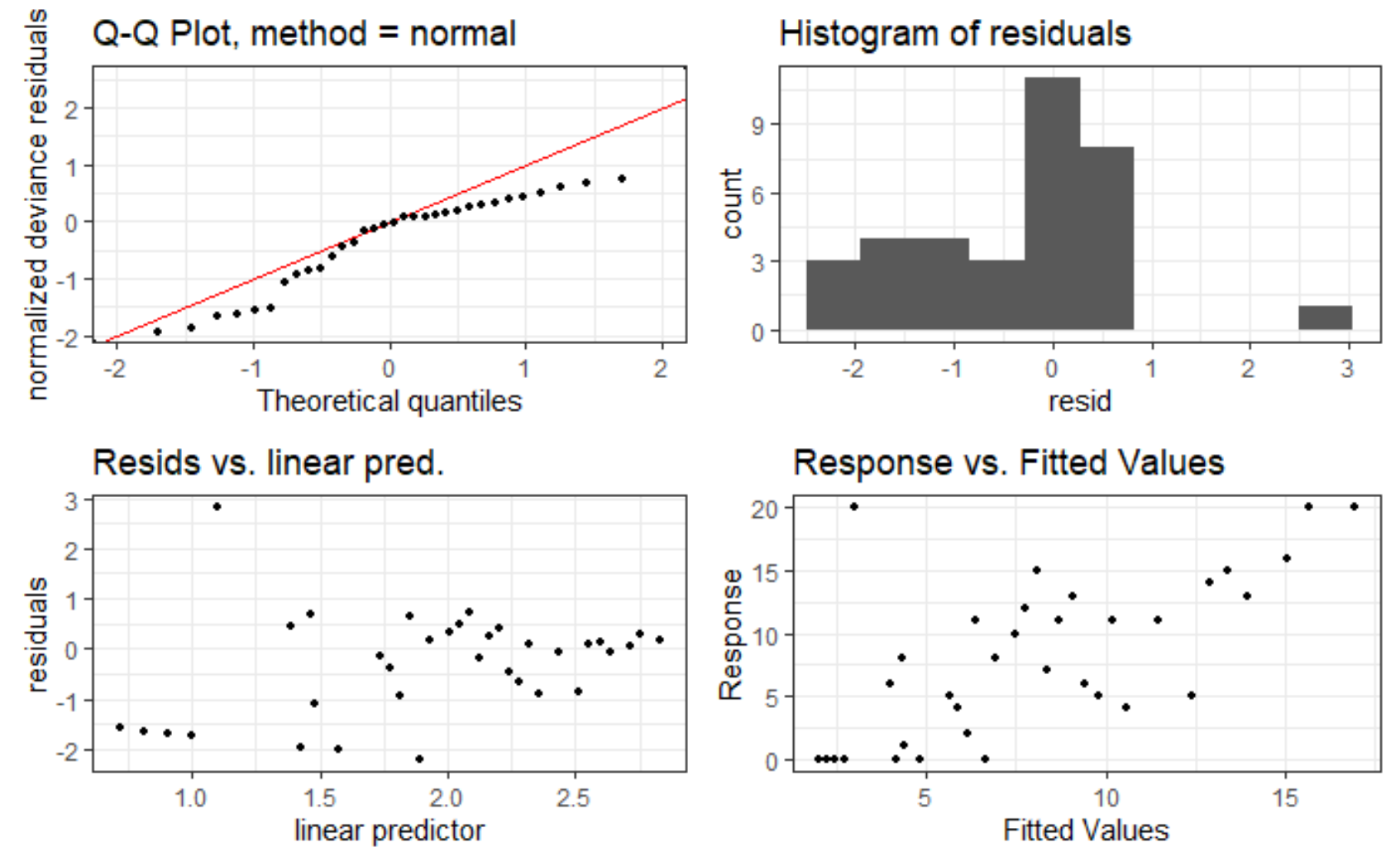 |
| CS Bogangolo | 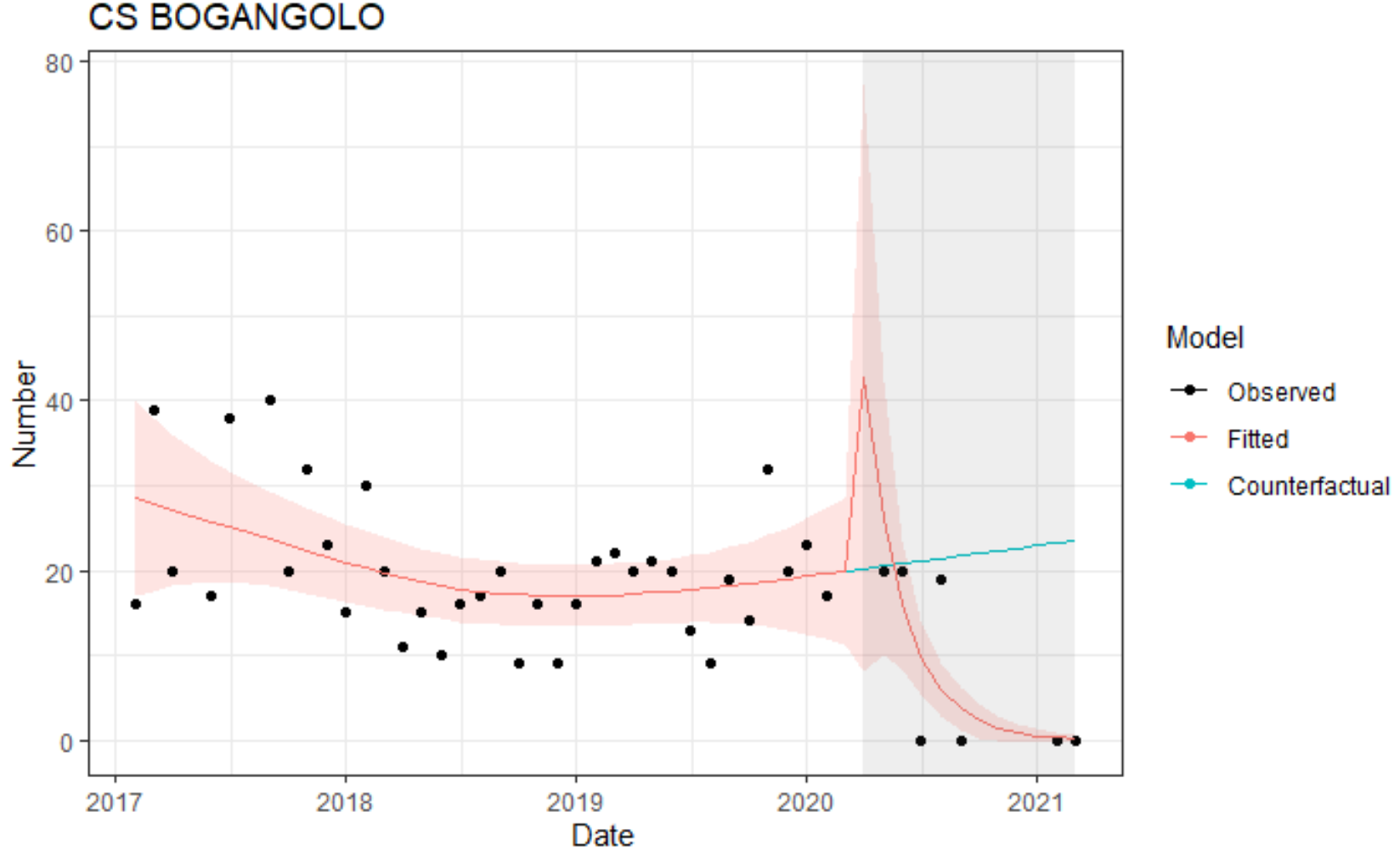 | 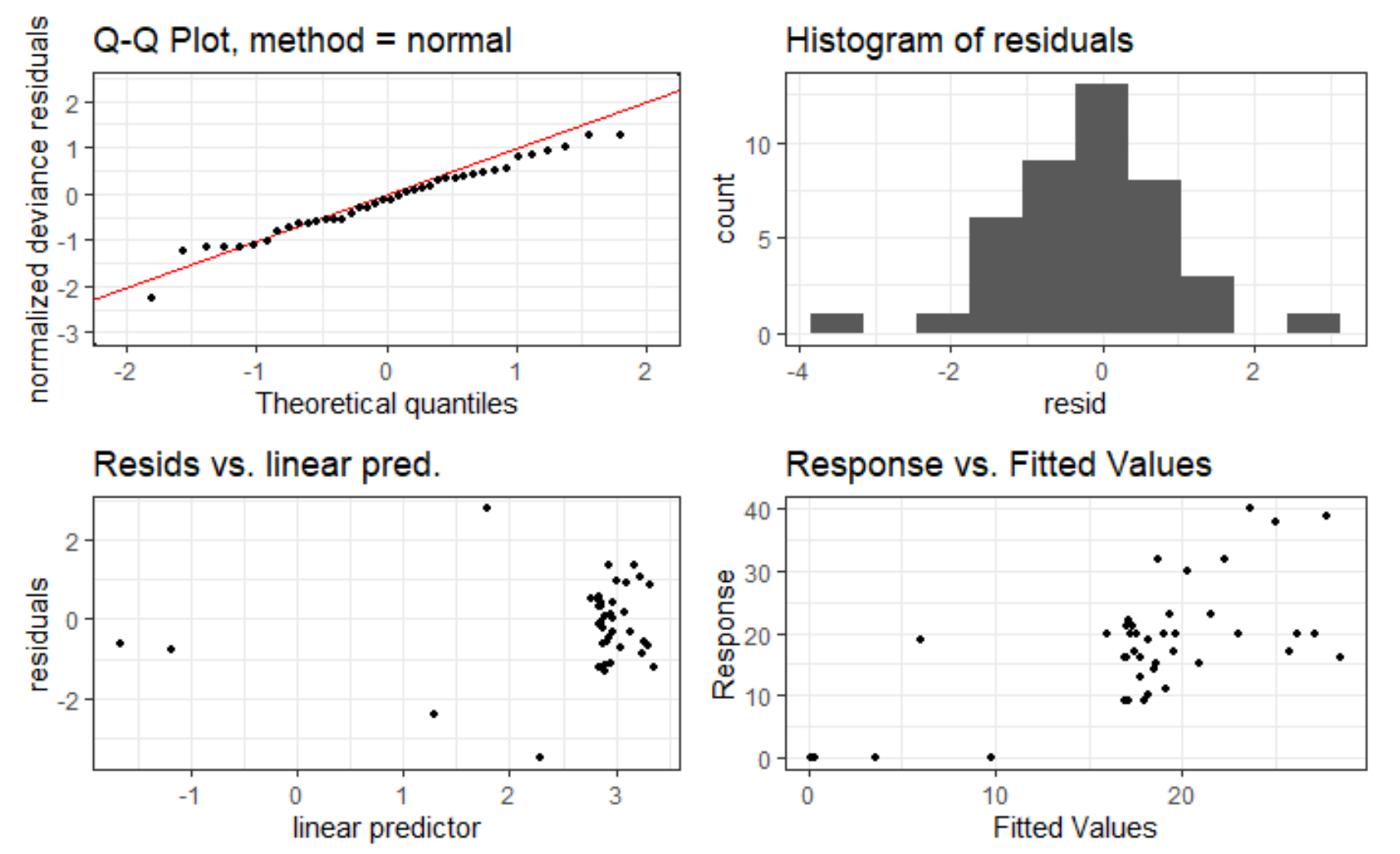 |
| CS Bouboui | 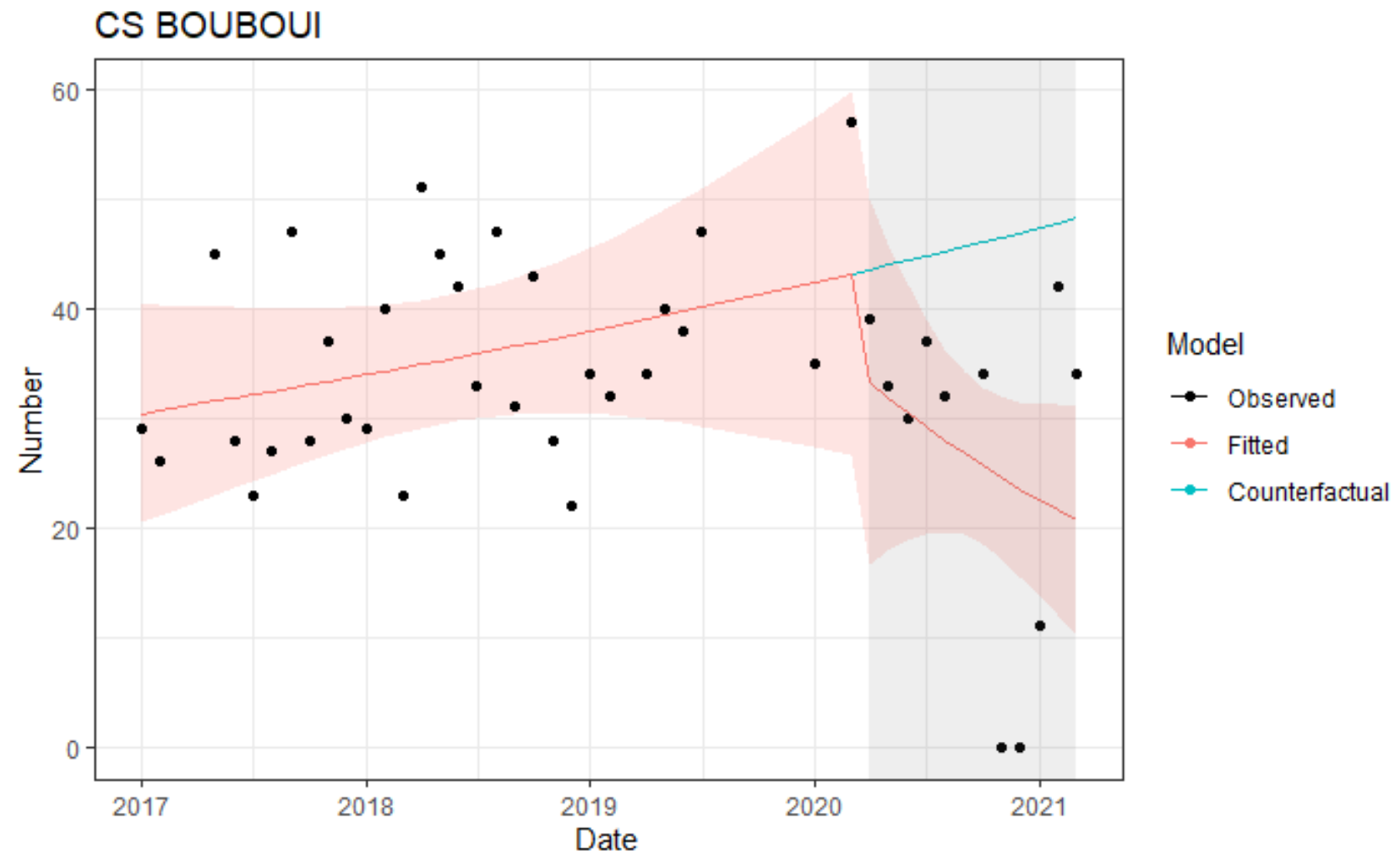 | 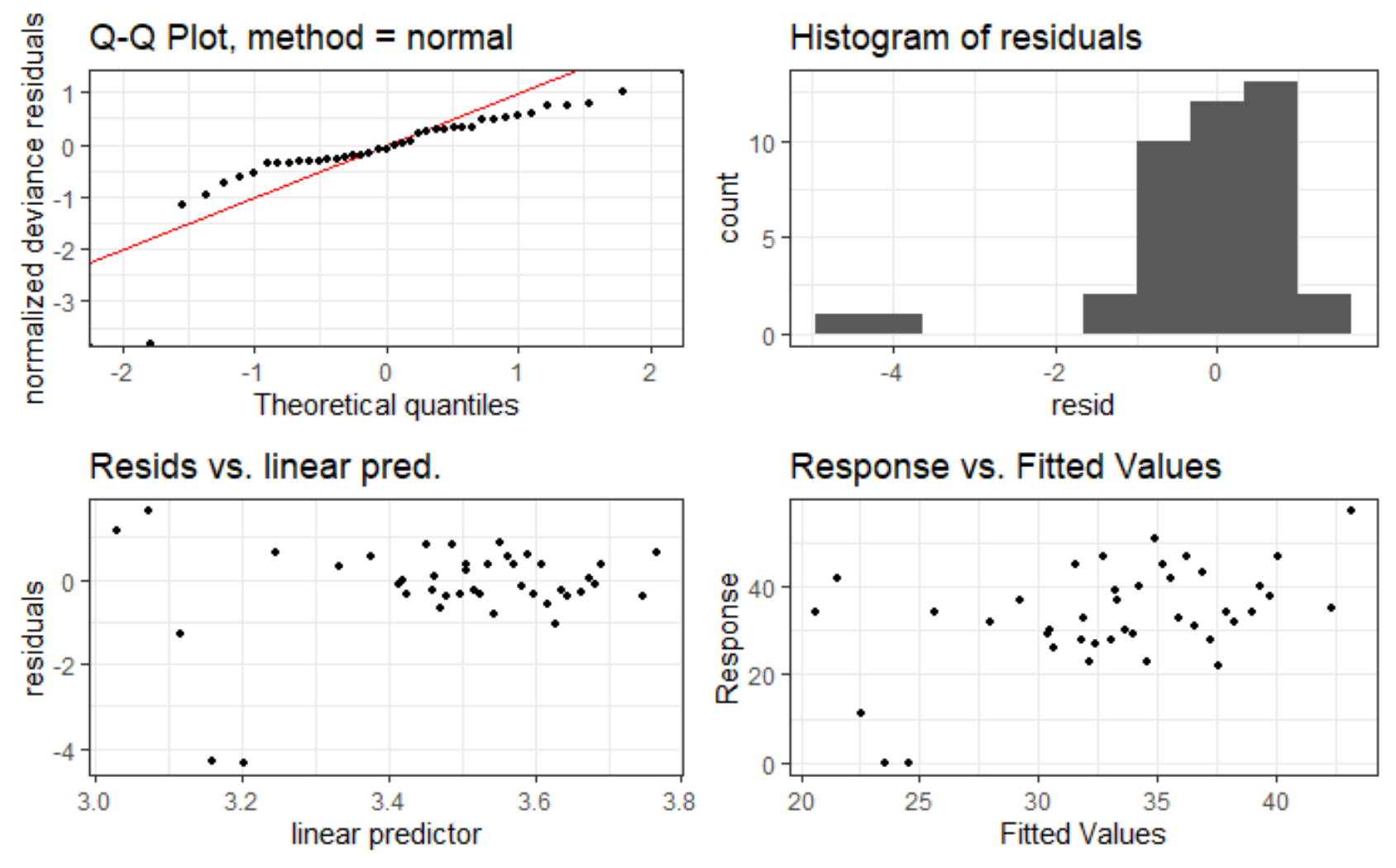 |
| CS Gbango | 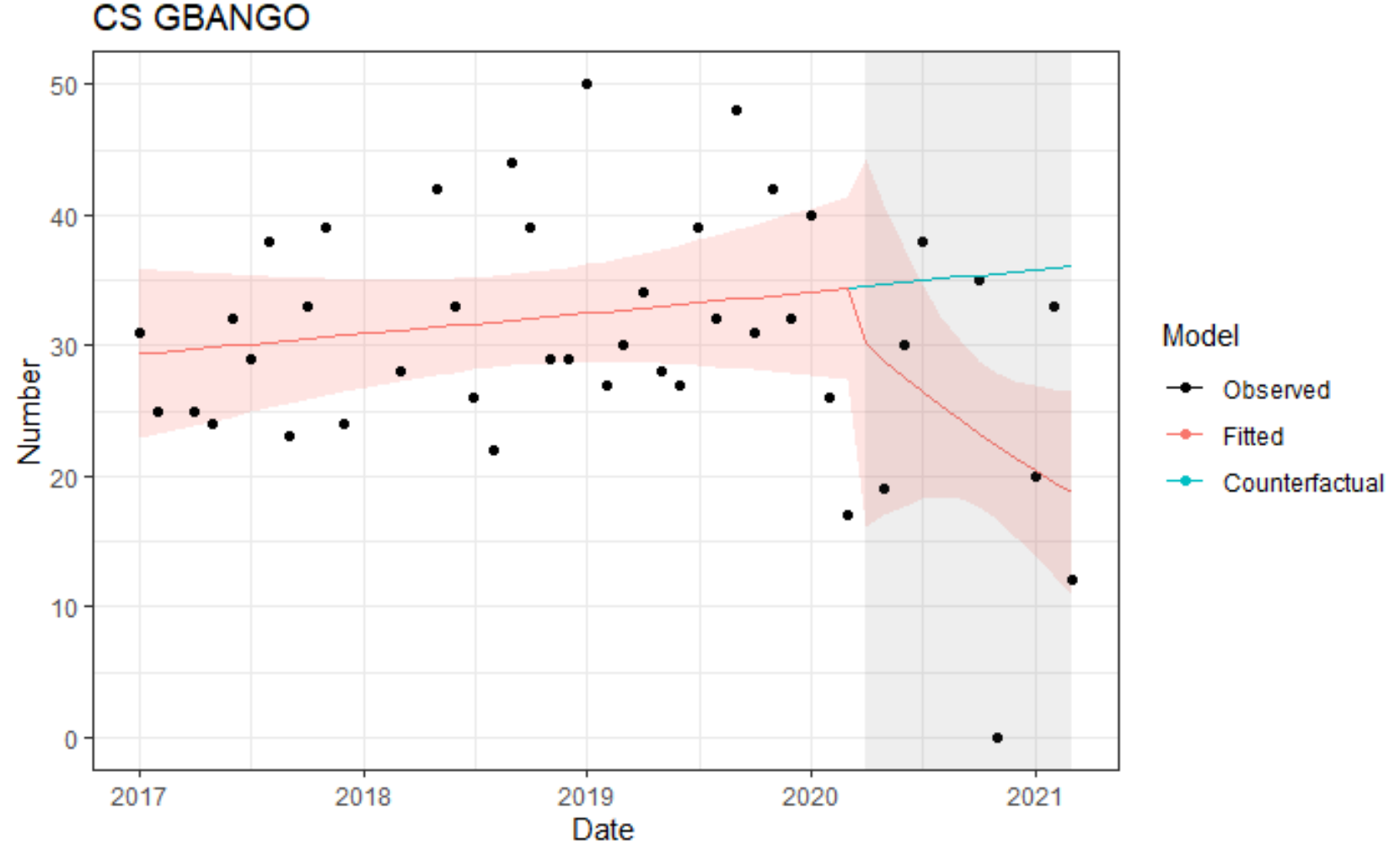 | 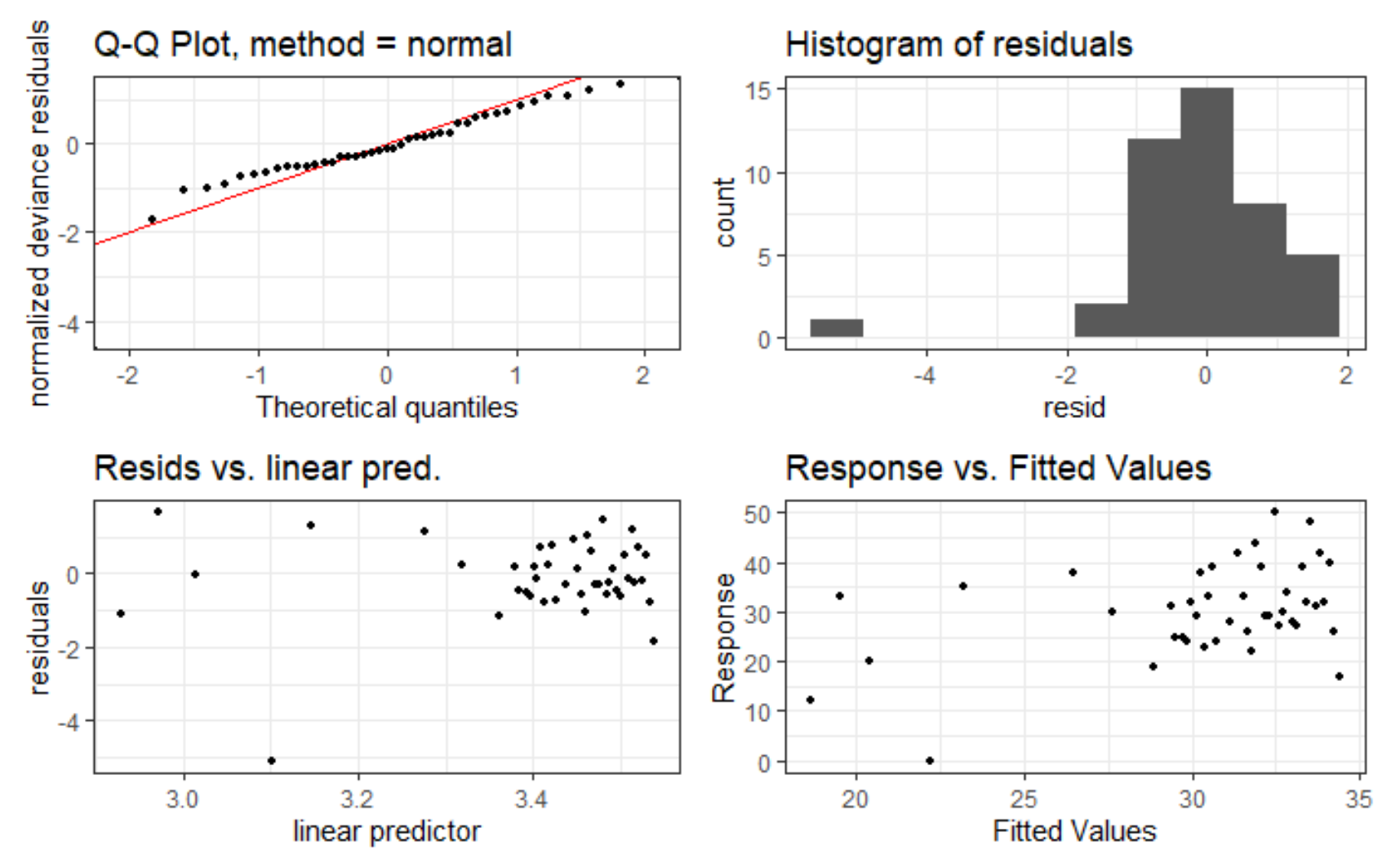 |
| CS Le Peuple | 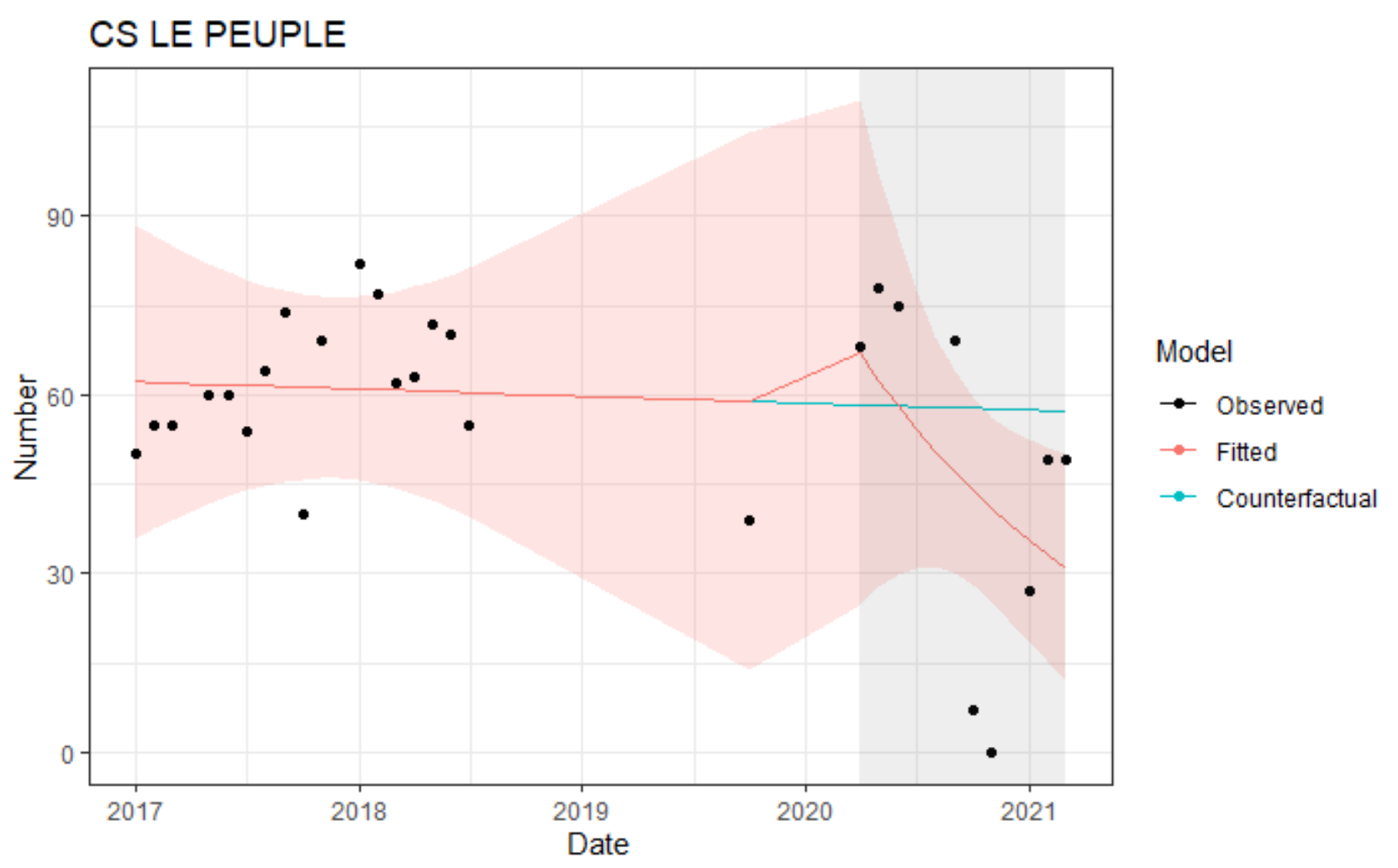 | 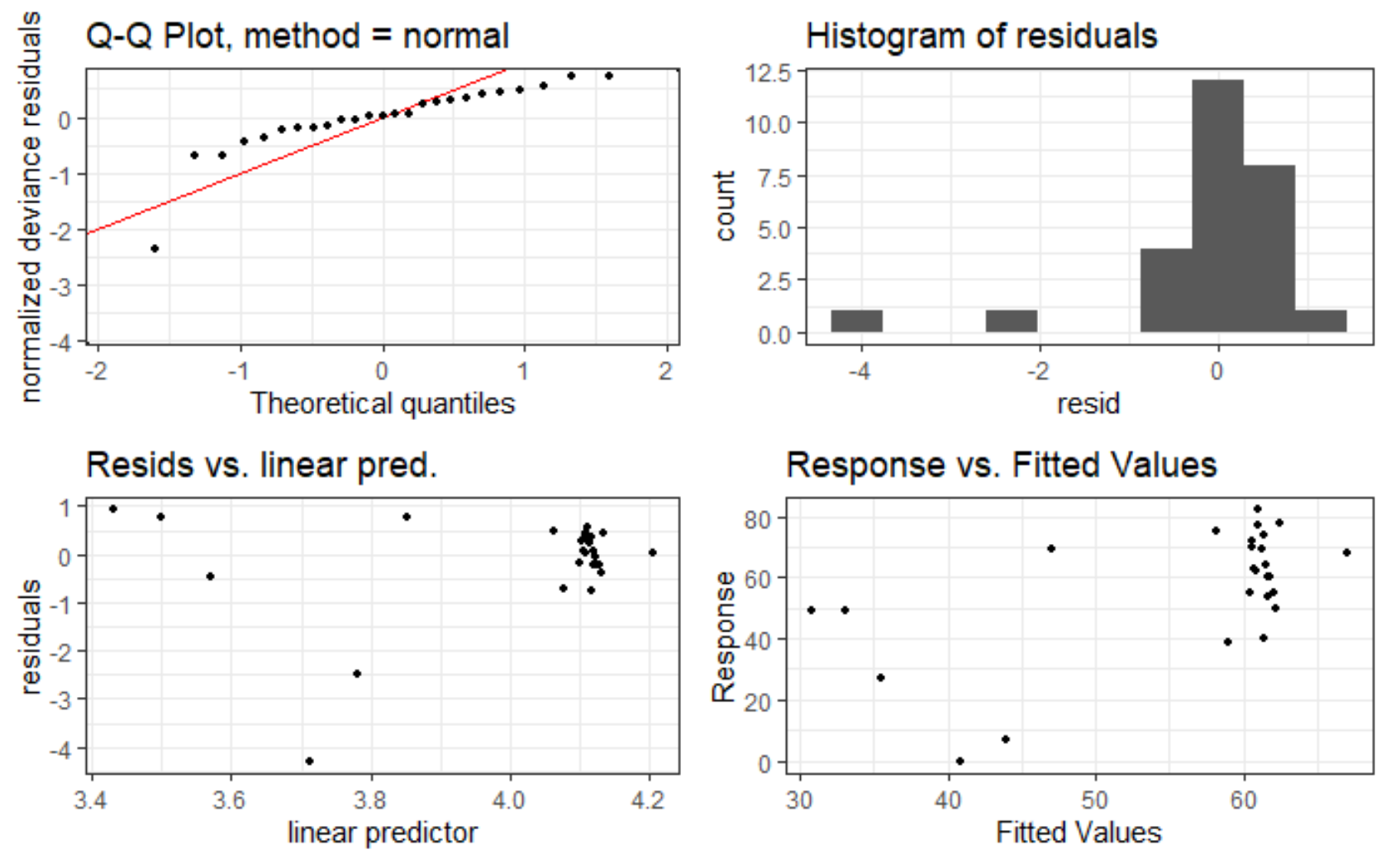 |
| CS Liton | 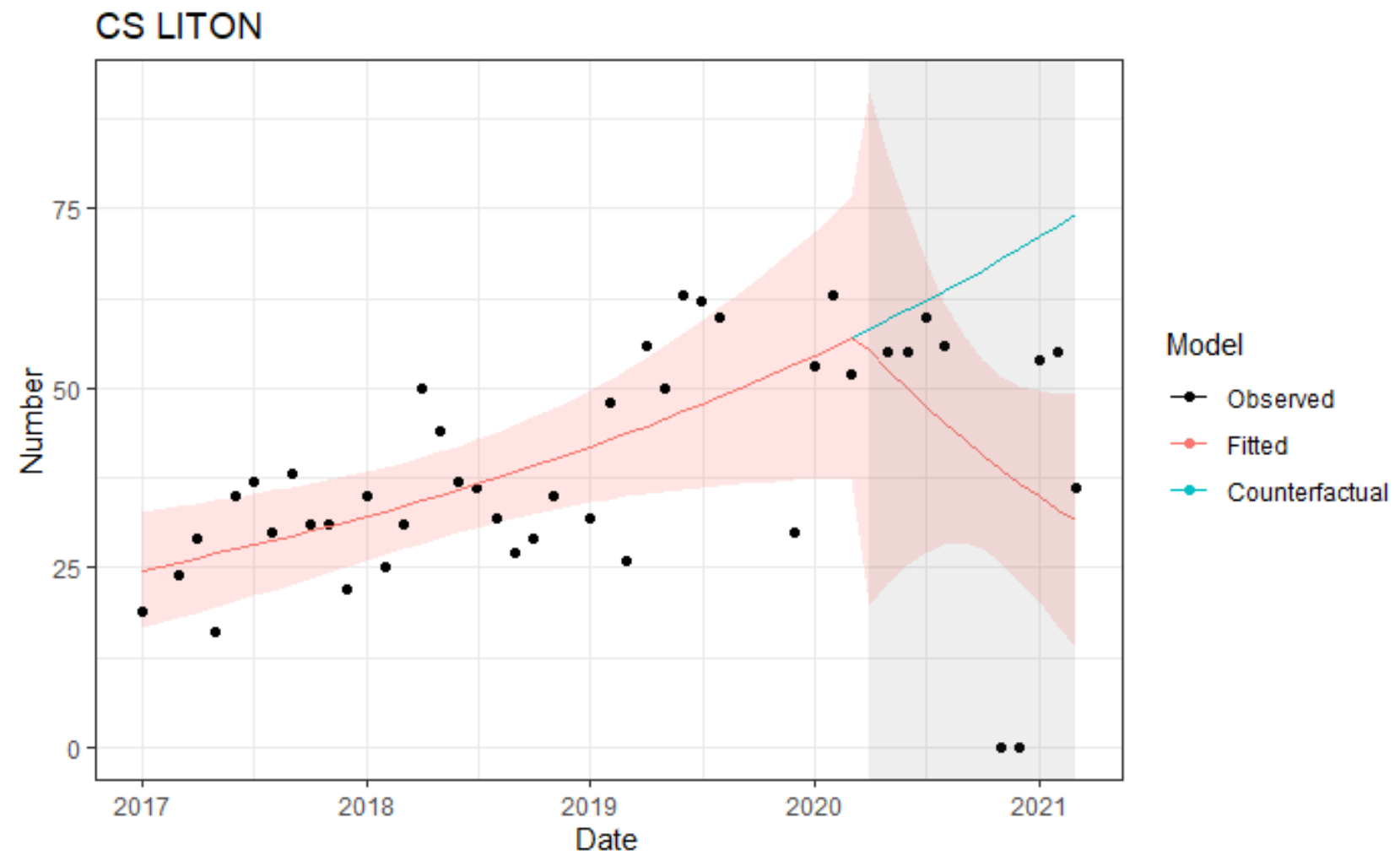 | 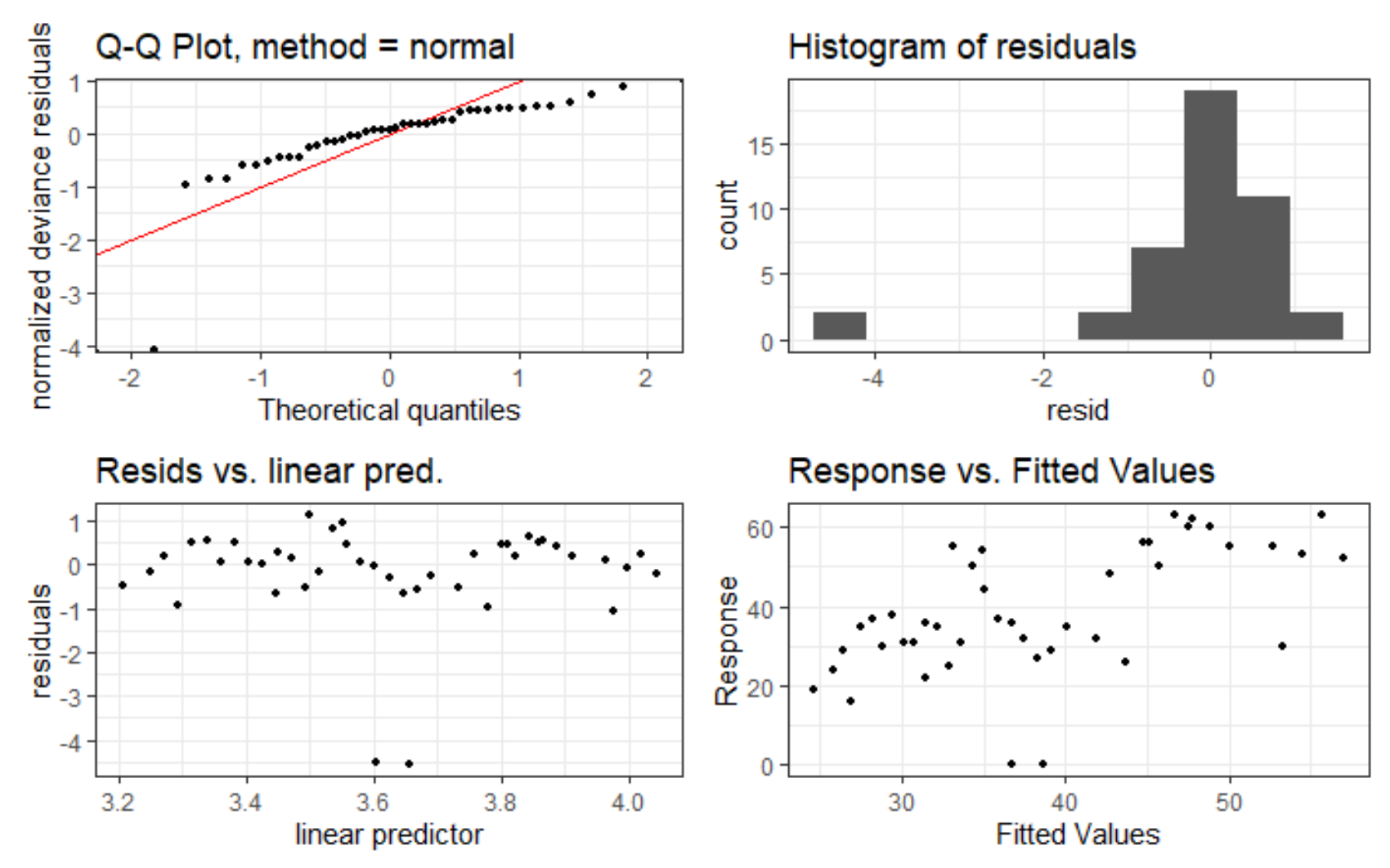 |
| HD Begoua | 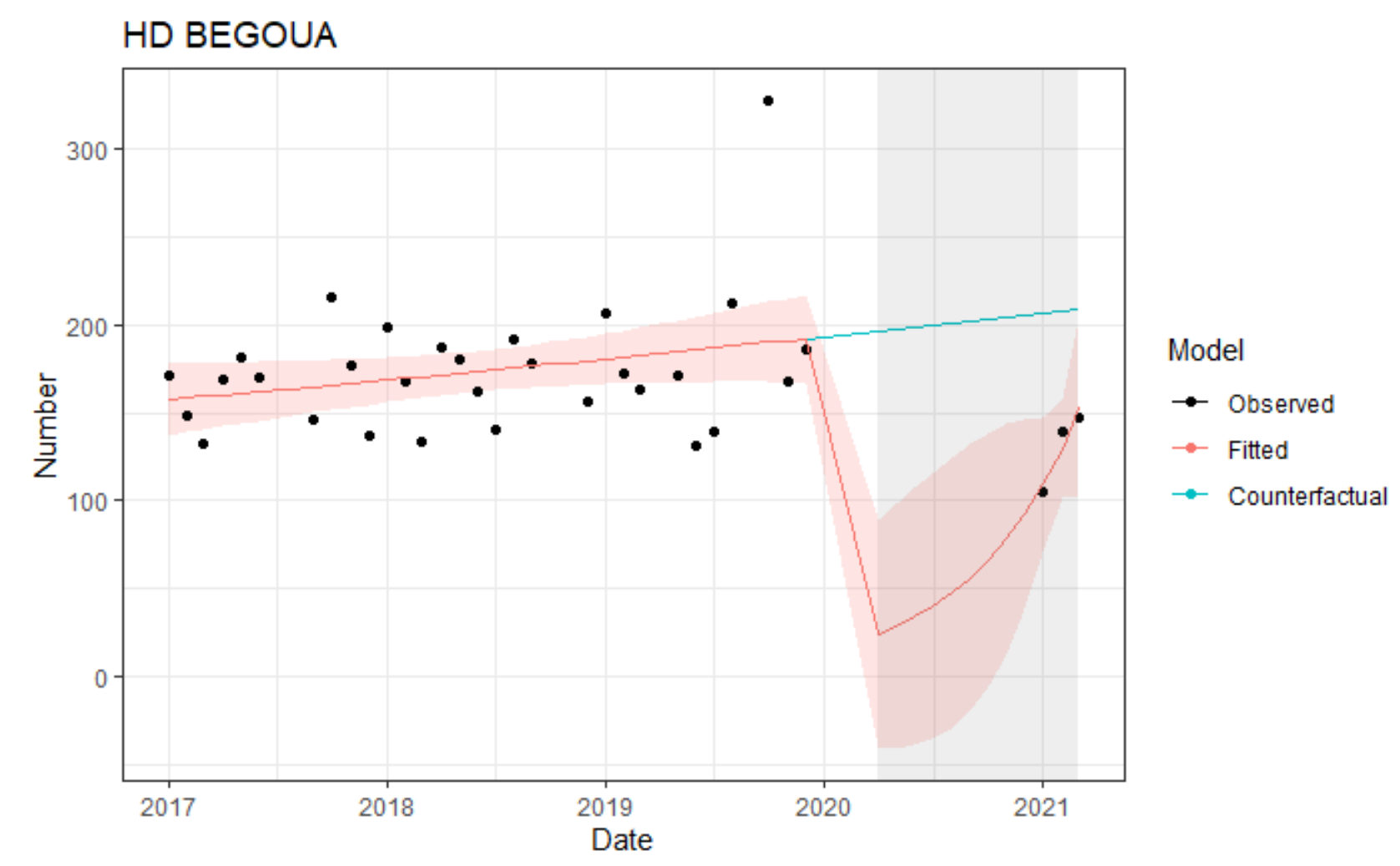 | 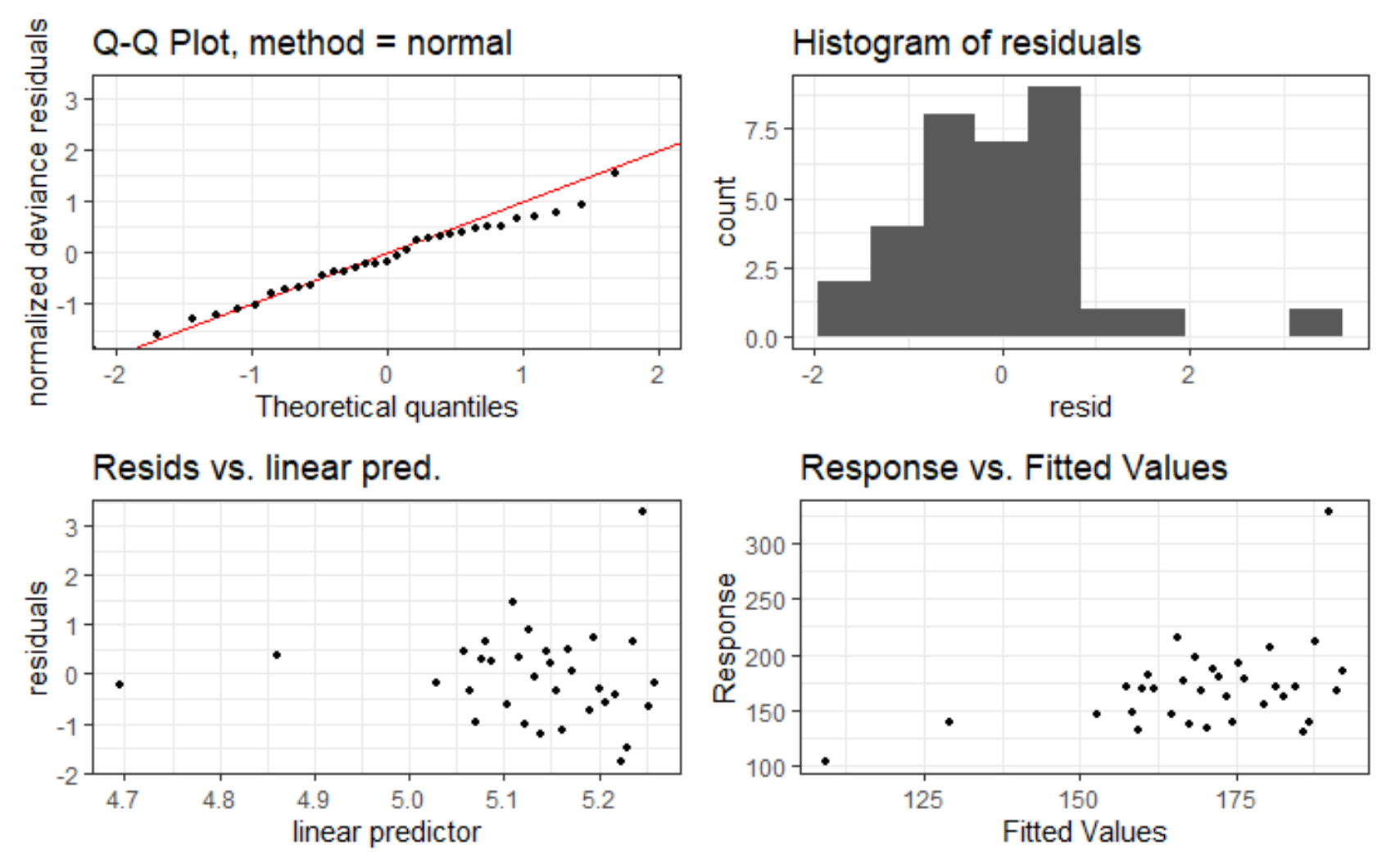 |
| PS Gbozo | 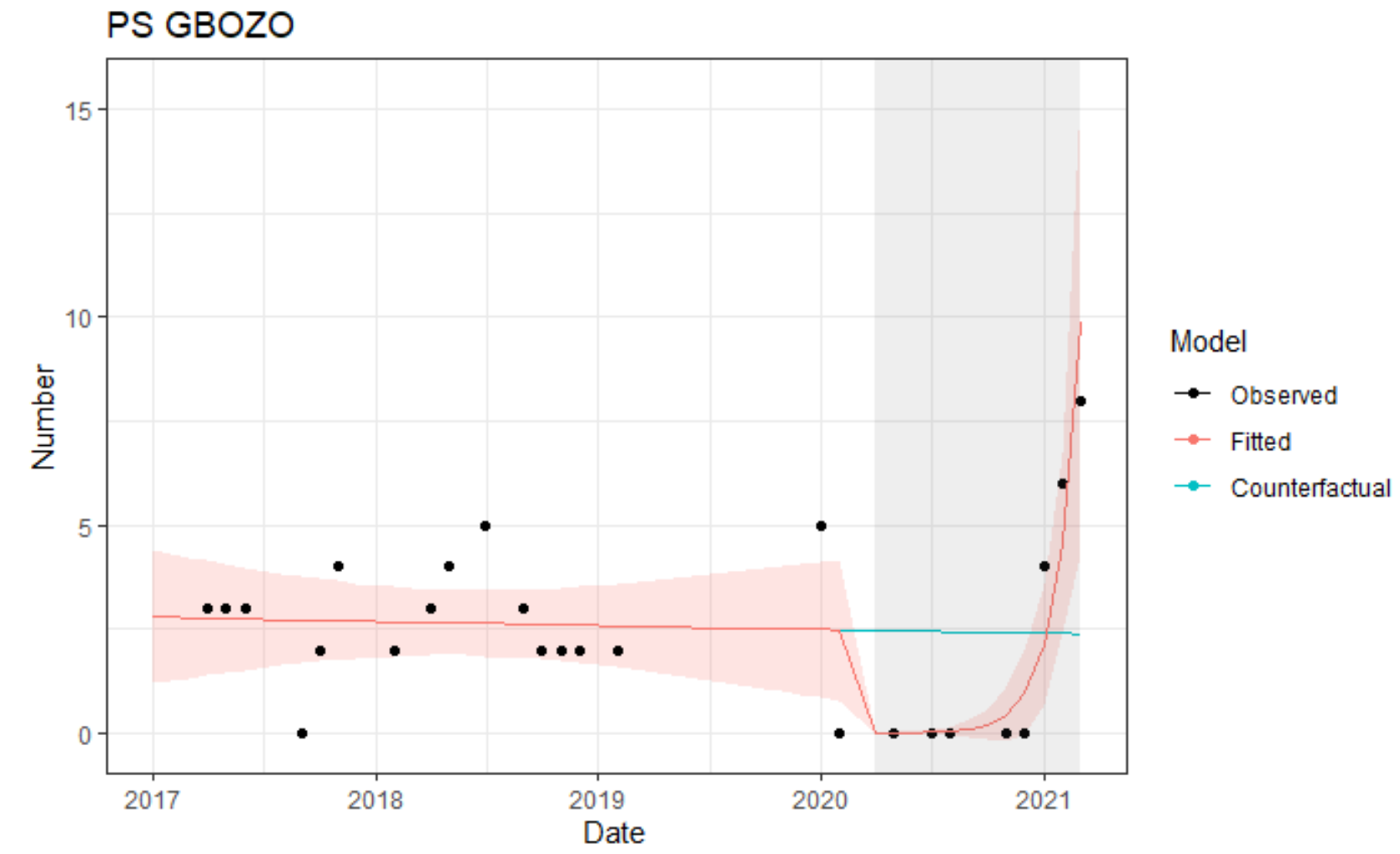 | 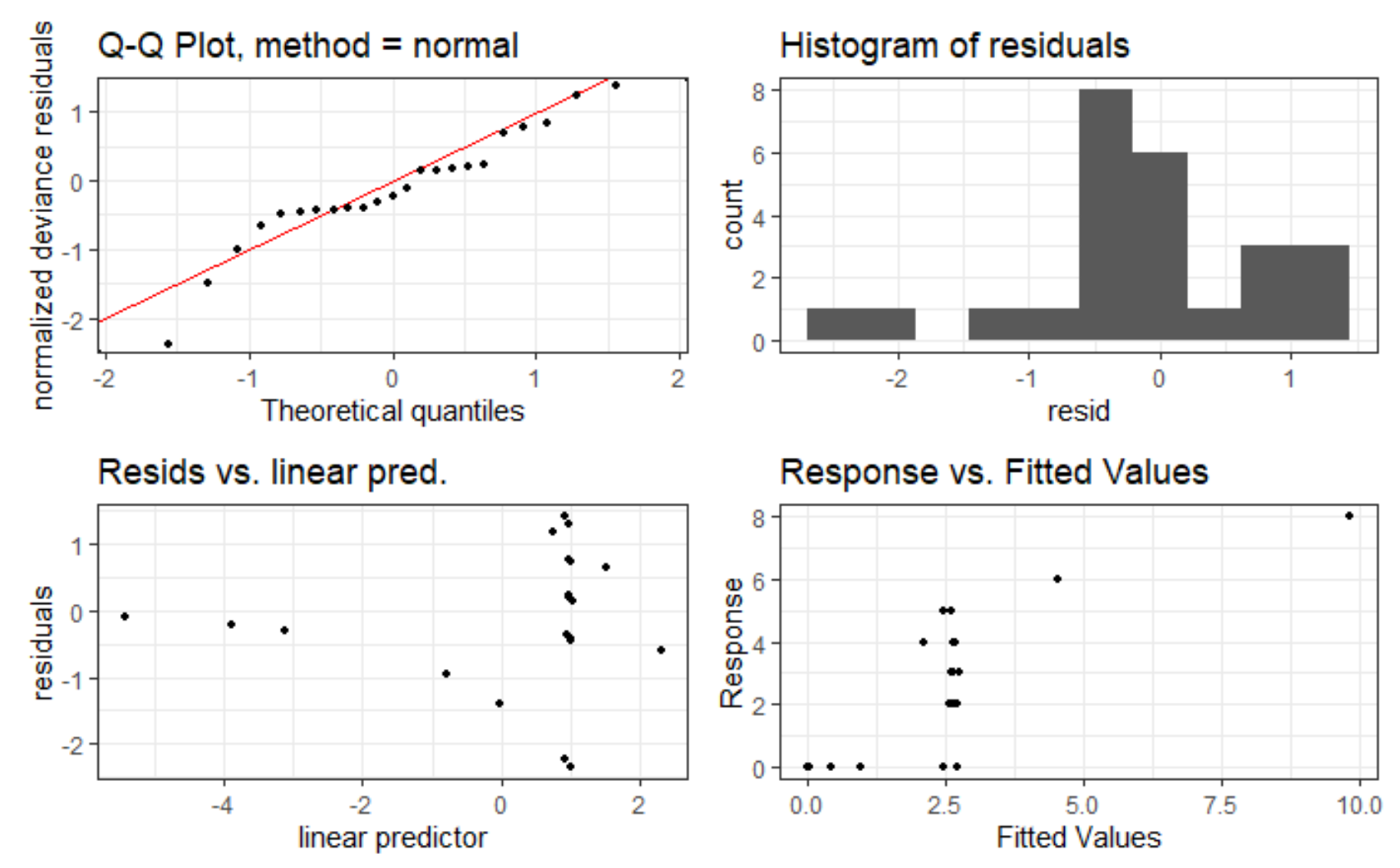 |

#### Bimbo

Note: excluded CS SALANGA, PS DAMECA because of unrealistic counterfactual
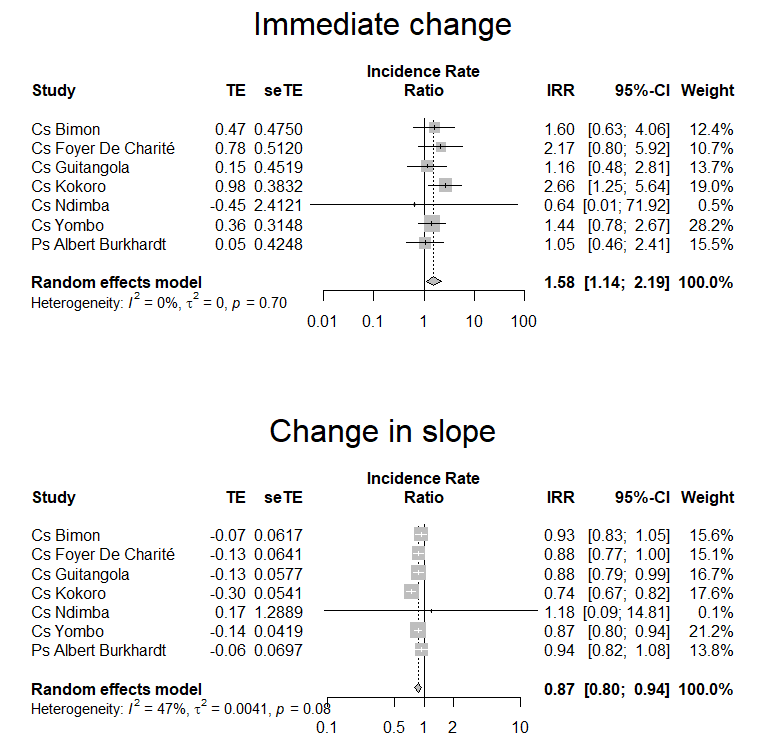


Model fit and residuals

| Facility | Model fit | Residuals |
| --- | --- | --- |
| CS Bimon | 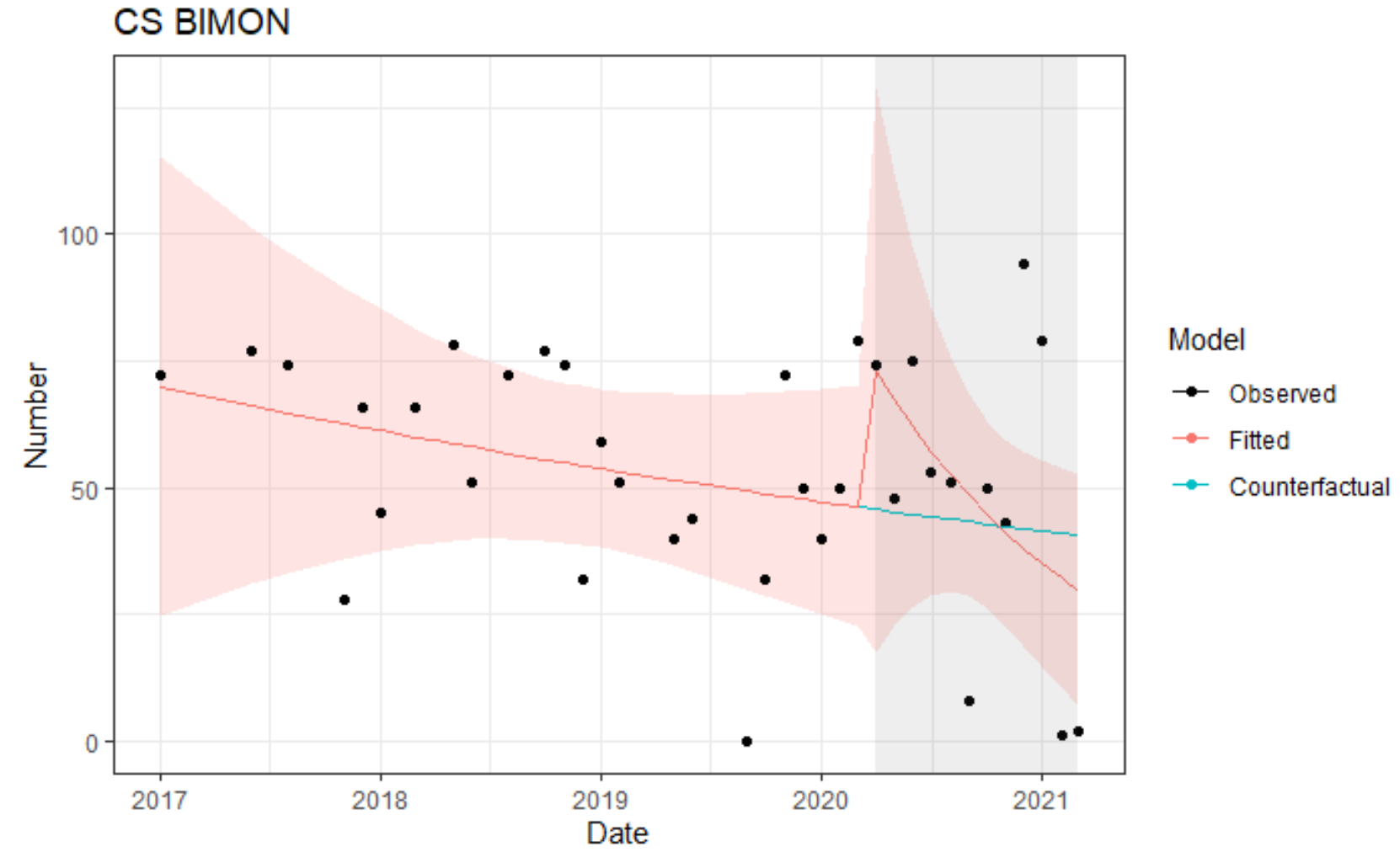 | 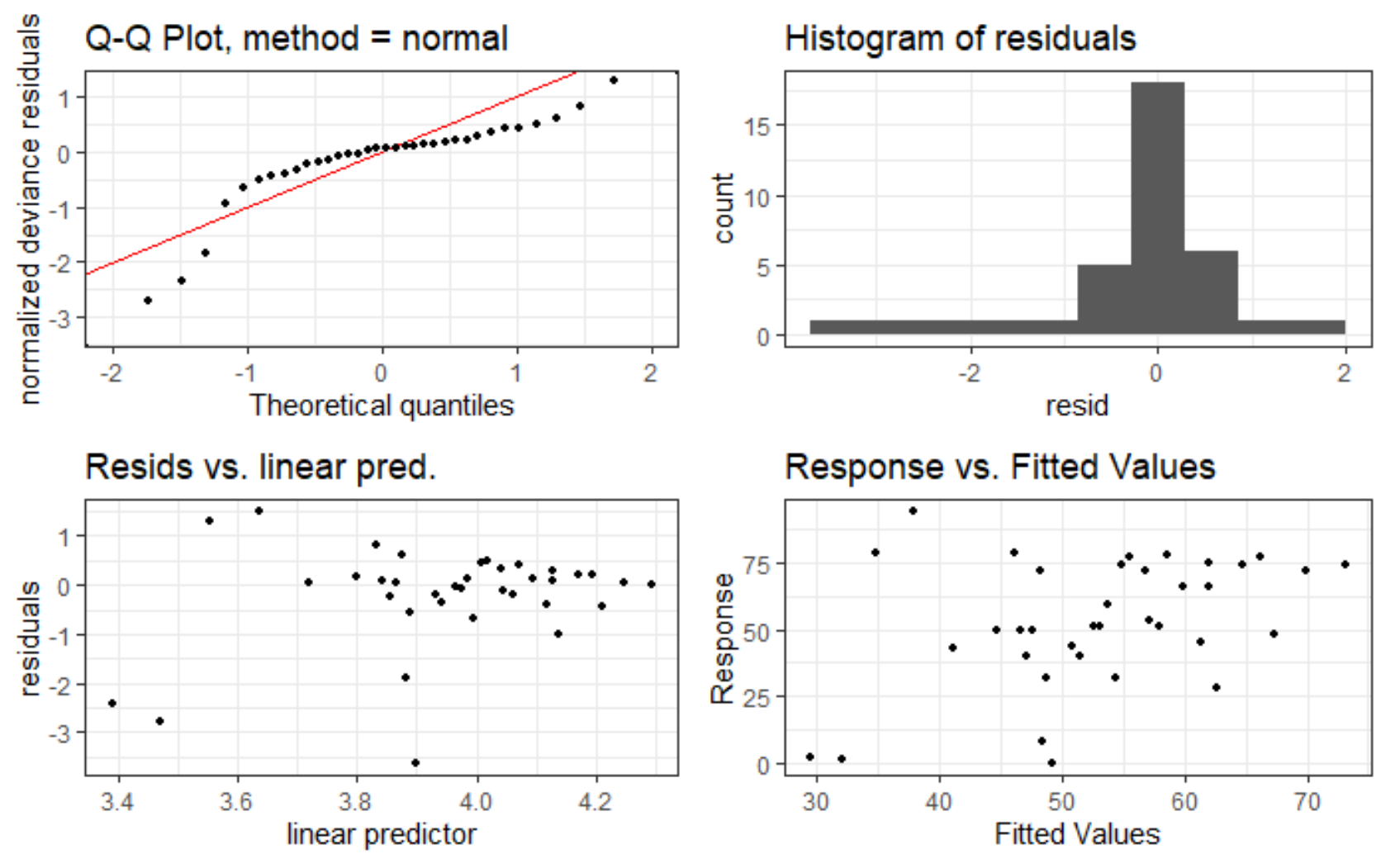 |
| CS Foyer de Charite | 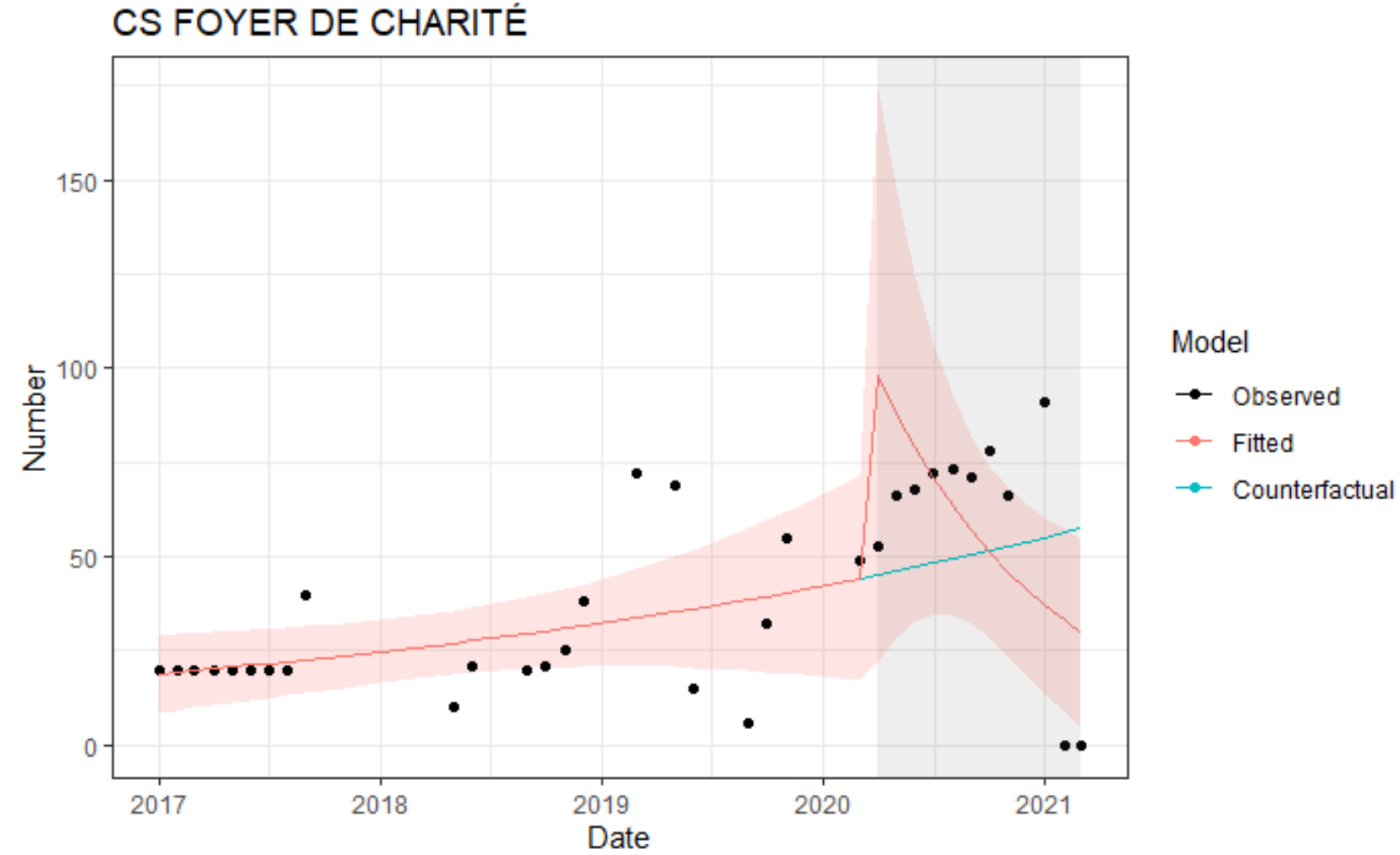 | 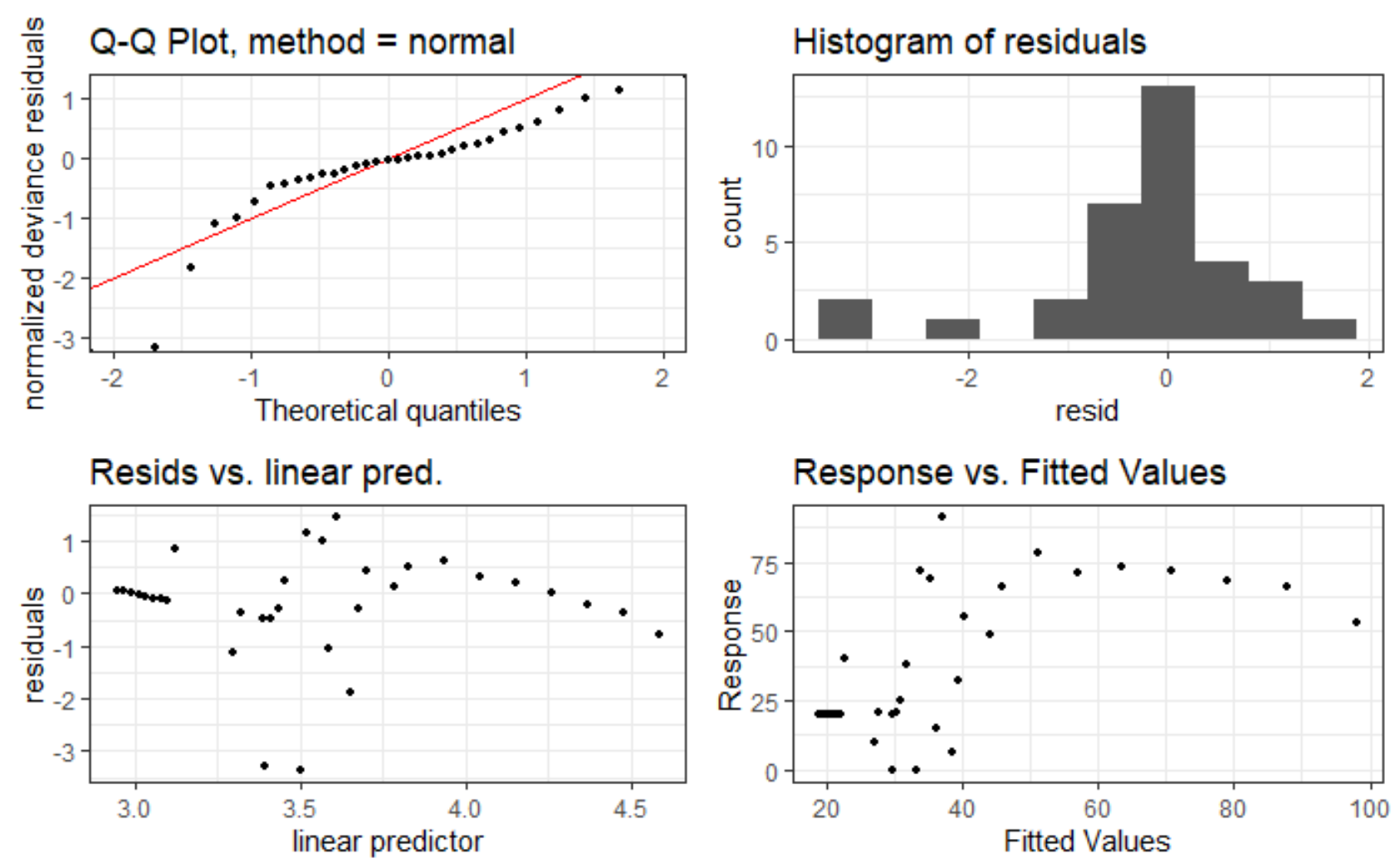 |
| CS Guitangola | 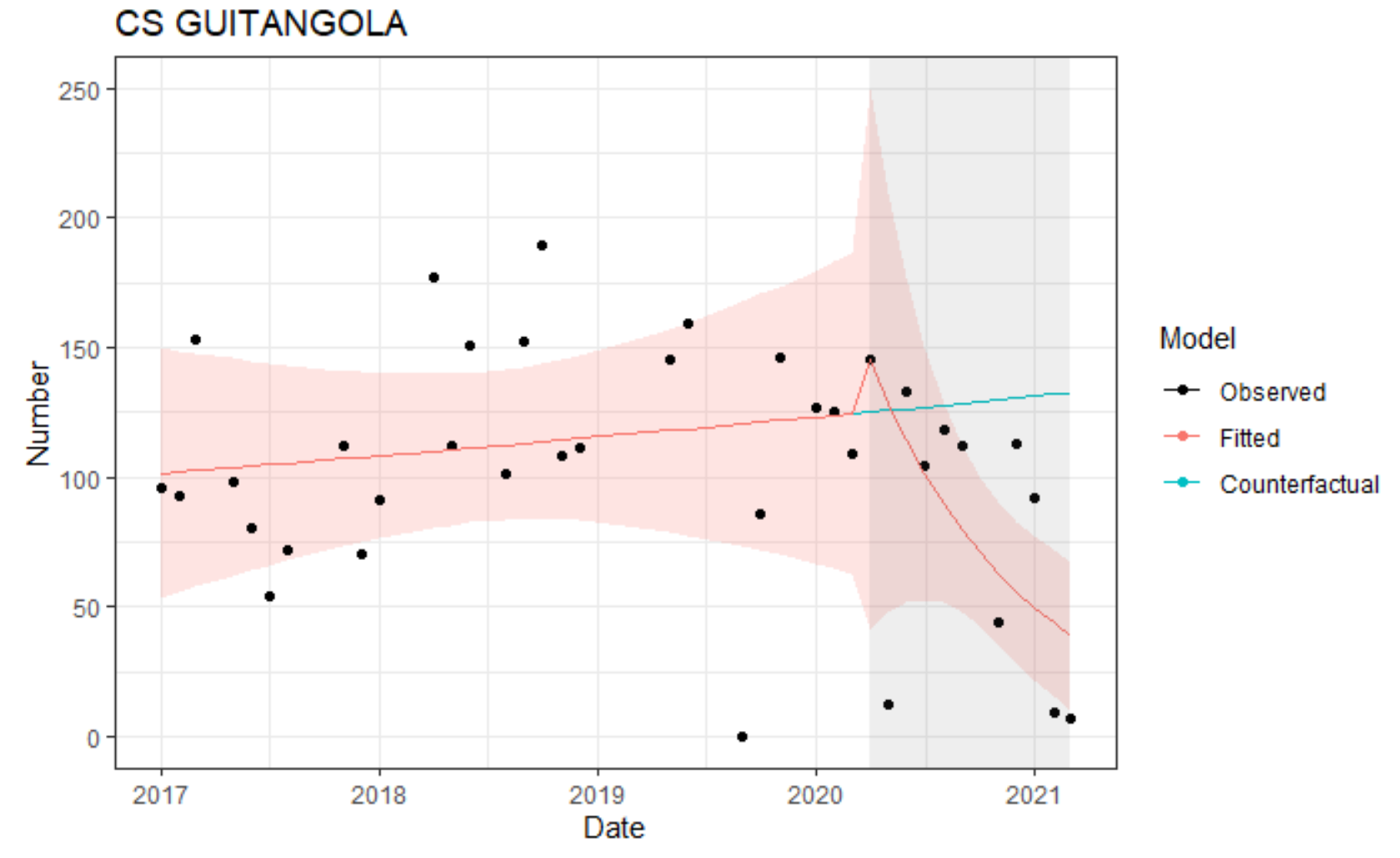 | 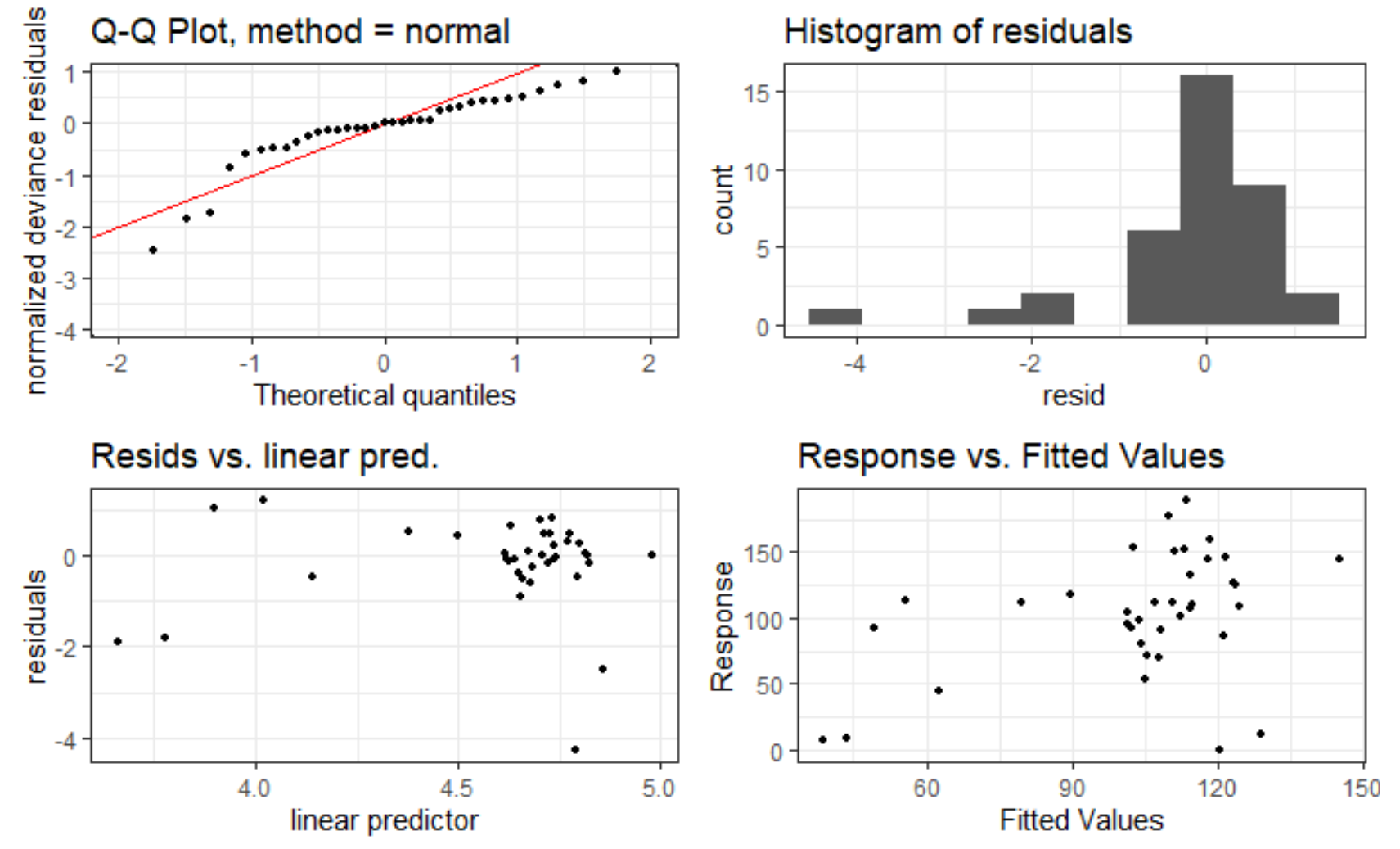 |
| CS Kokoro | 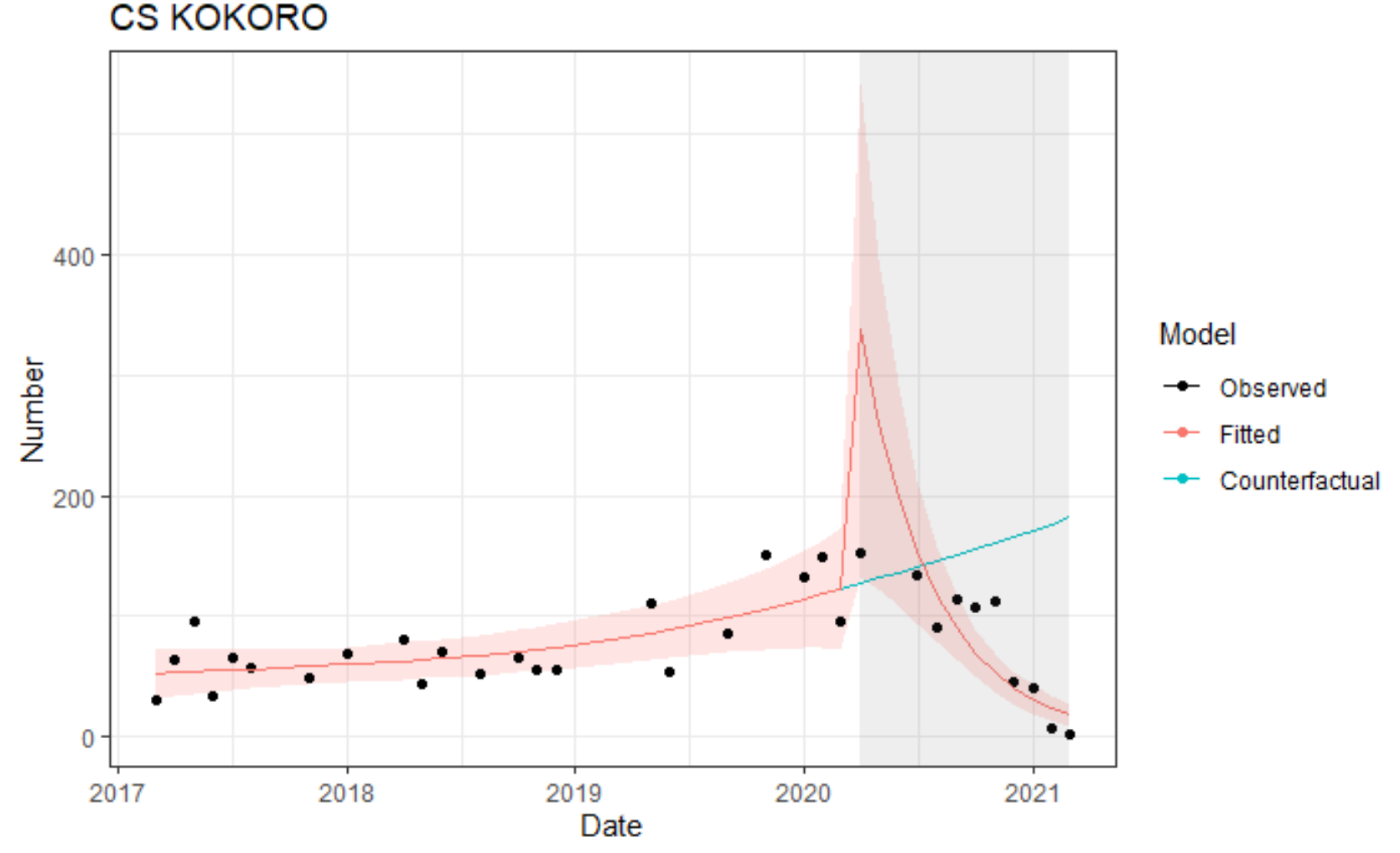 | 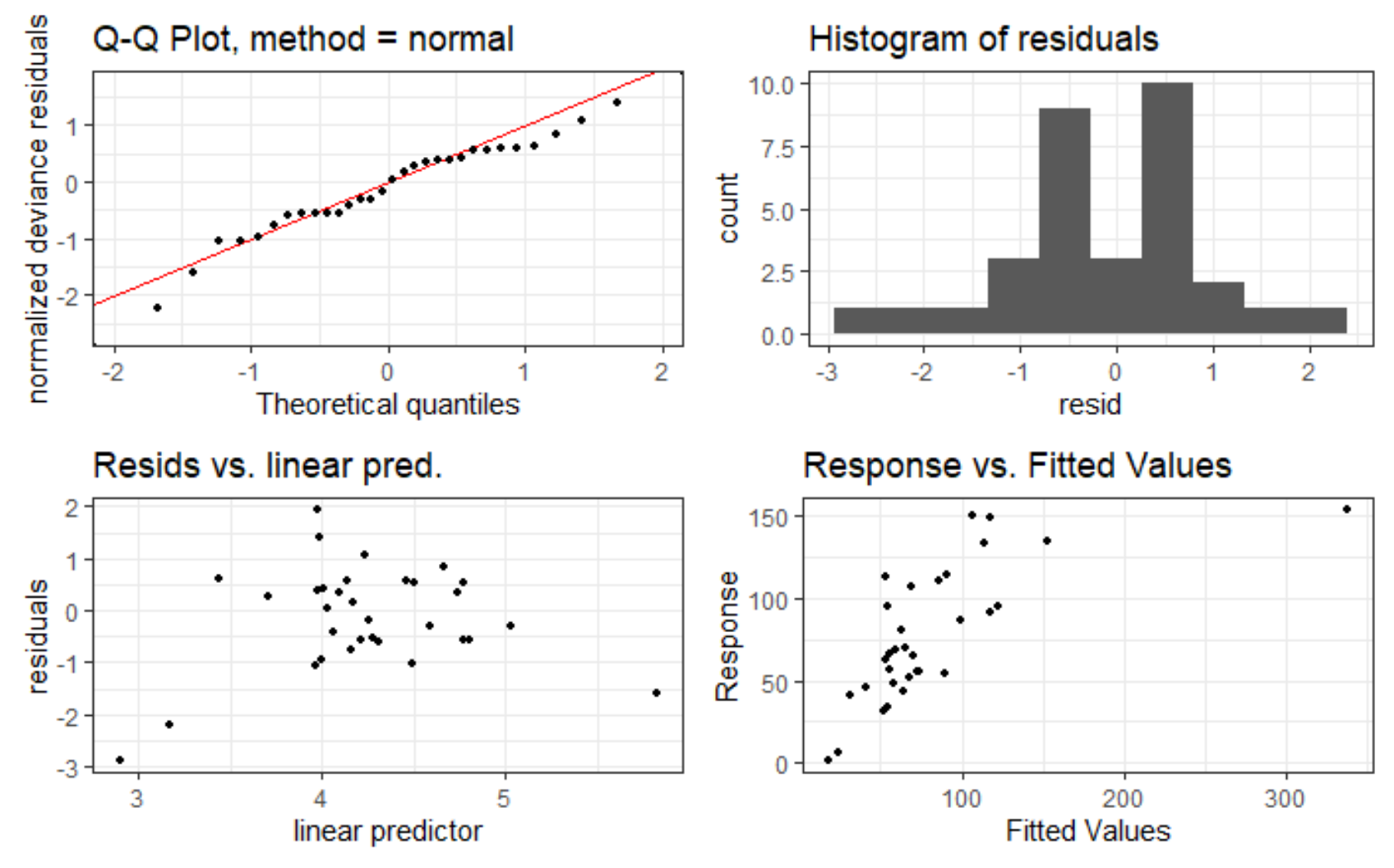 |
| CS Ndimba | 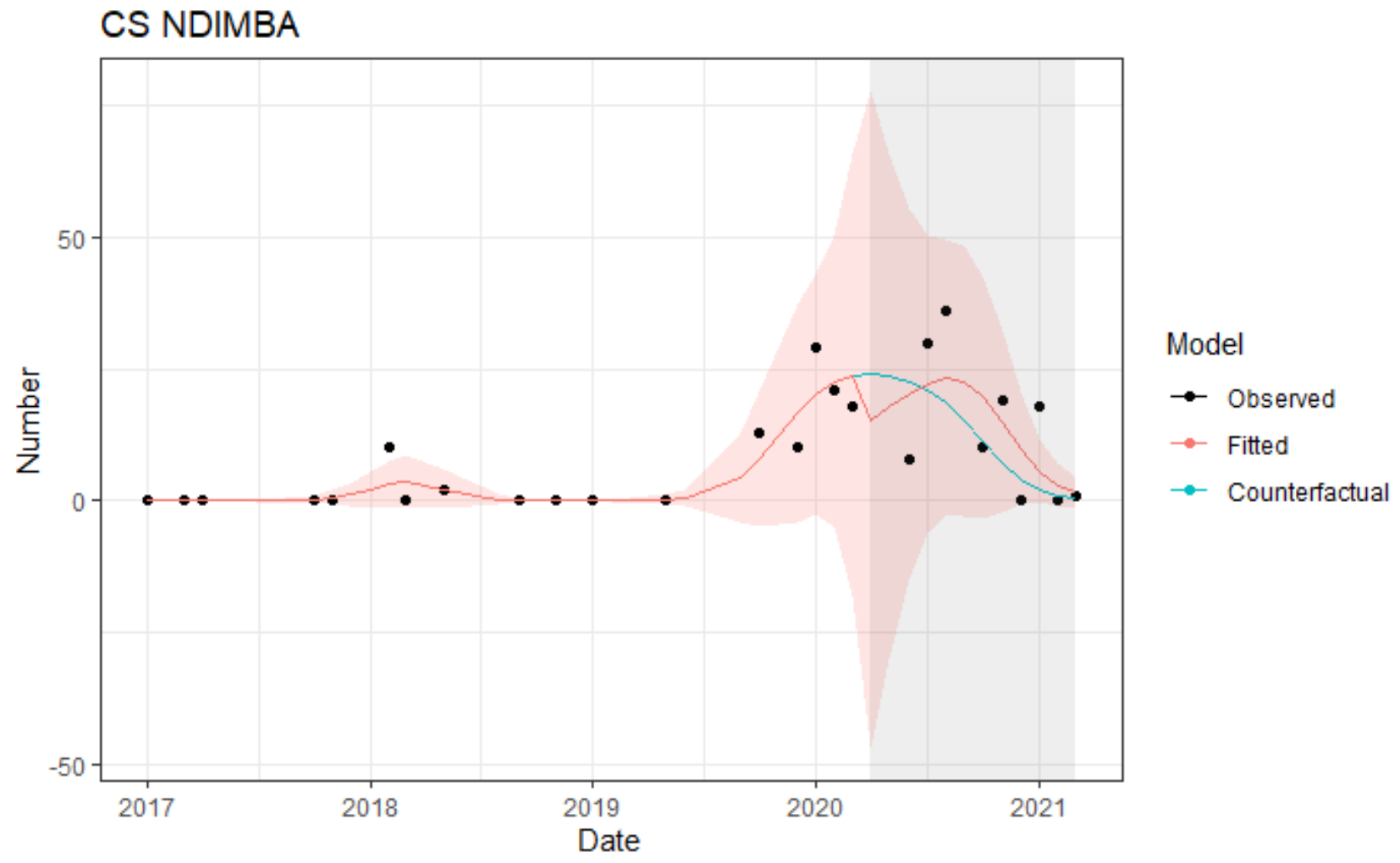 | 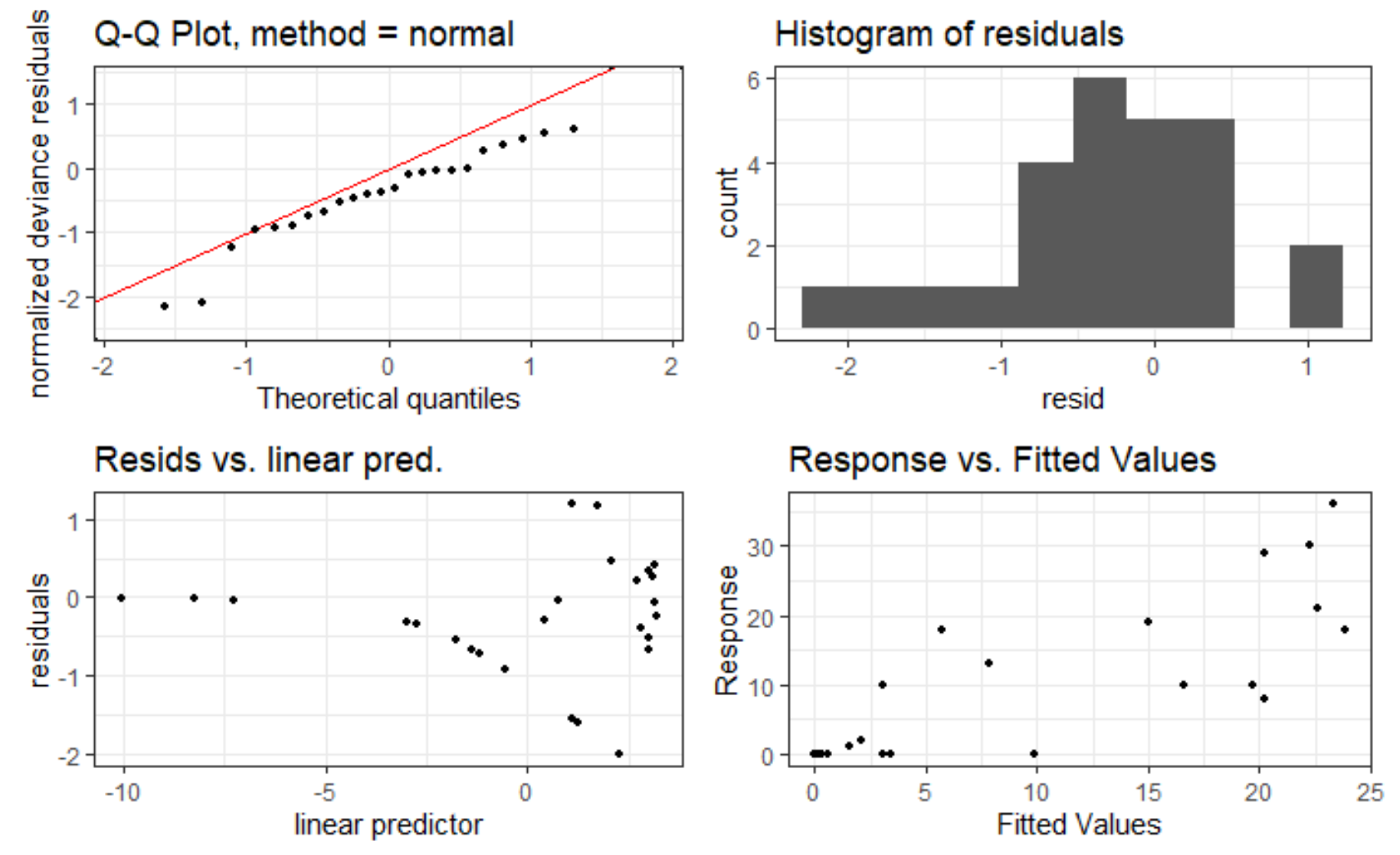 |
| CS Yombo | 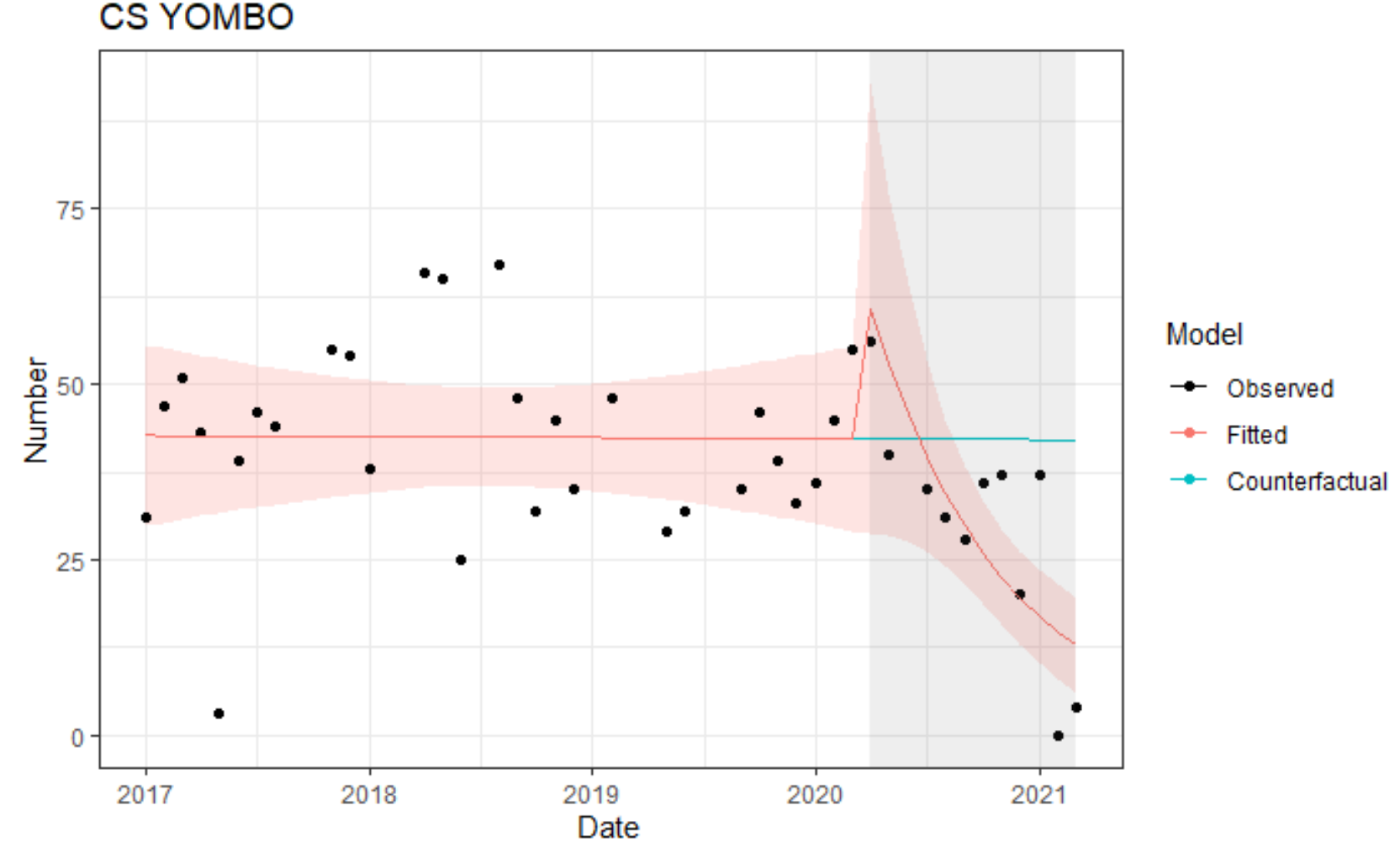 | 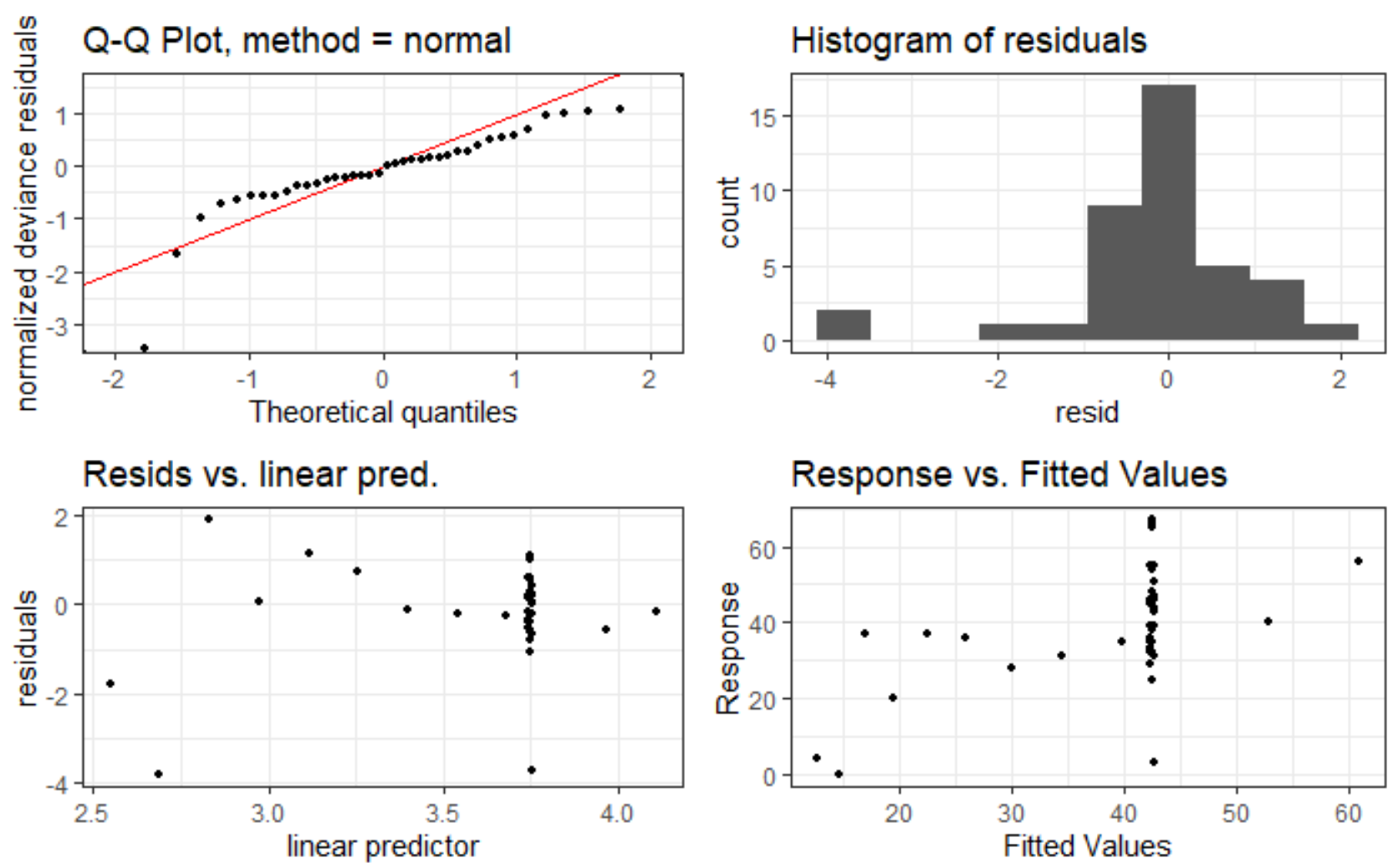 |
| PS Albert Burkhardt | 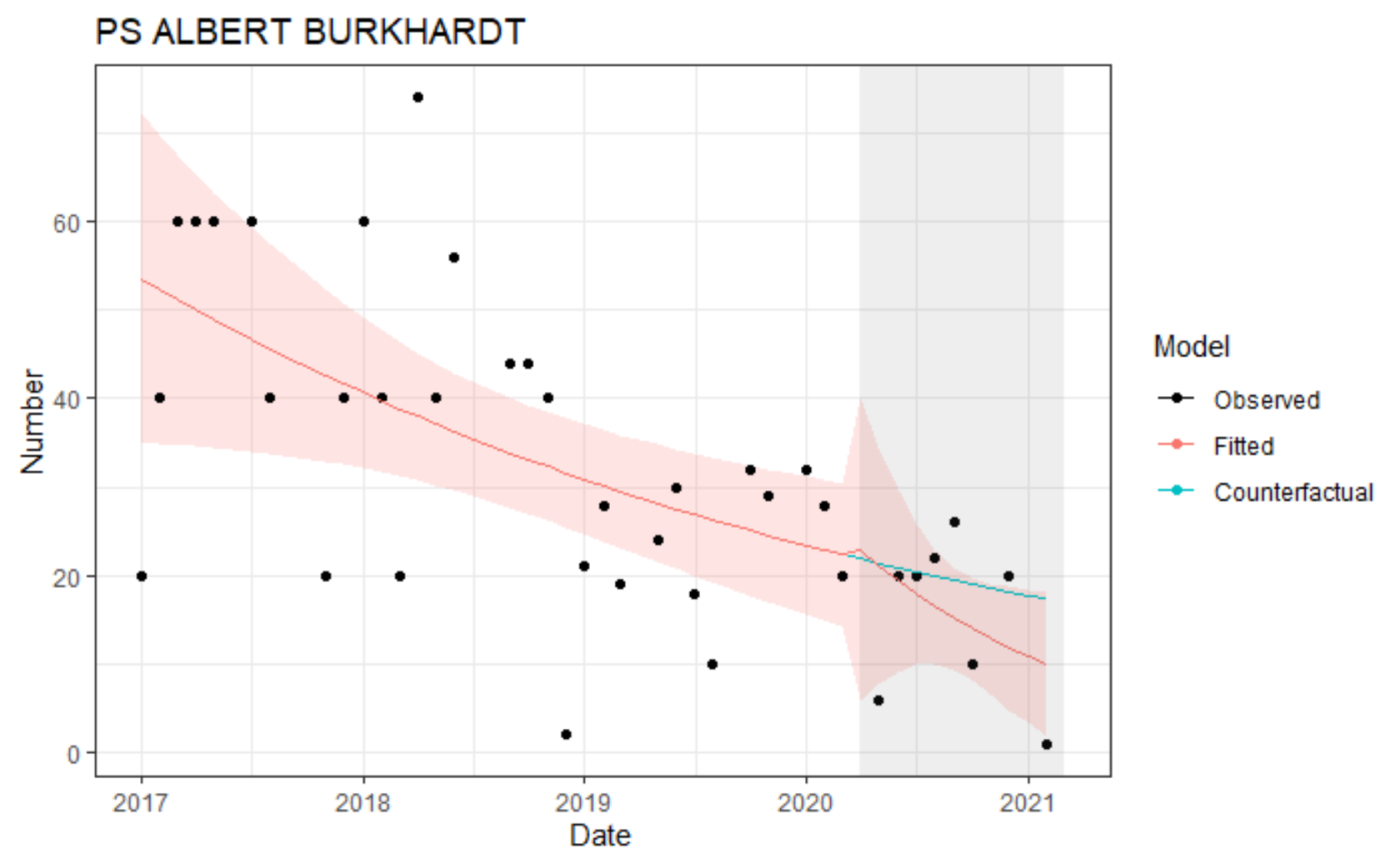 | 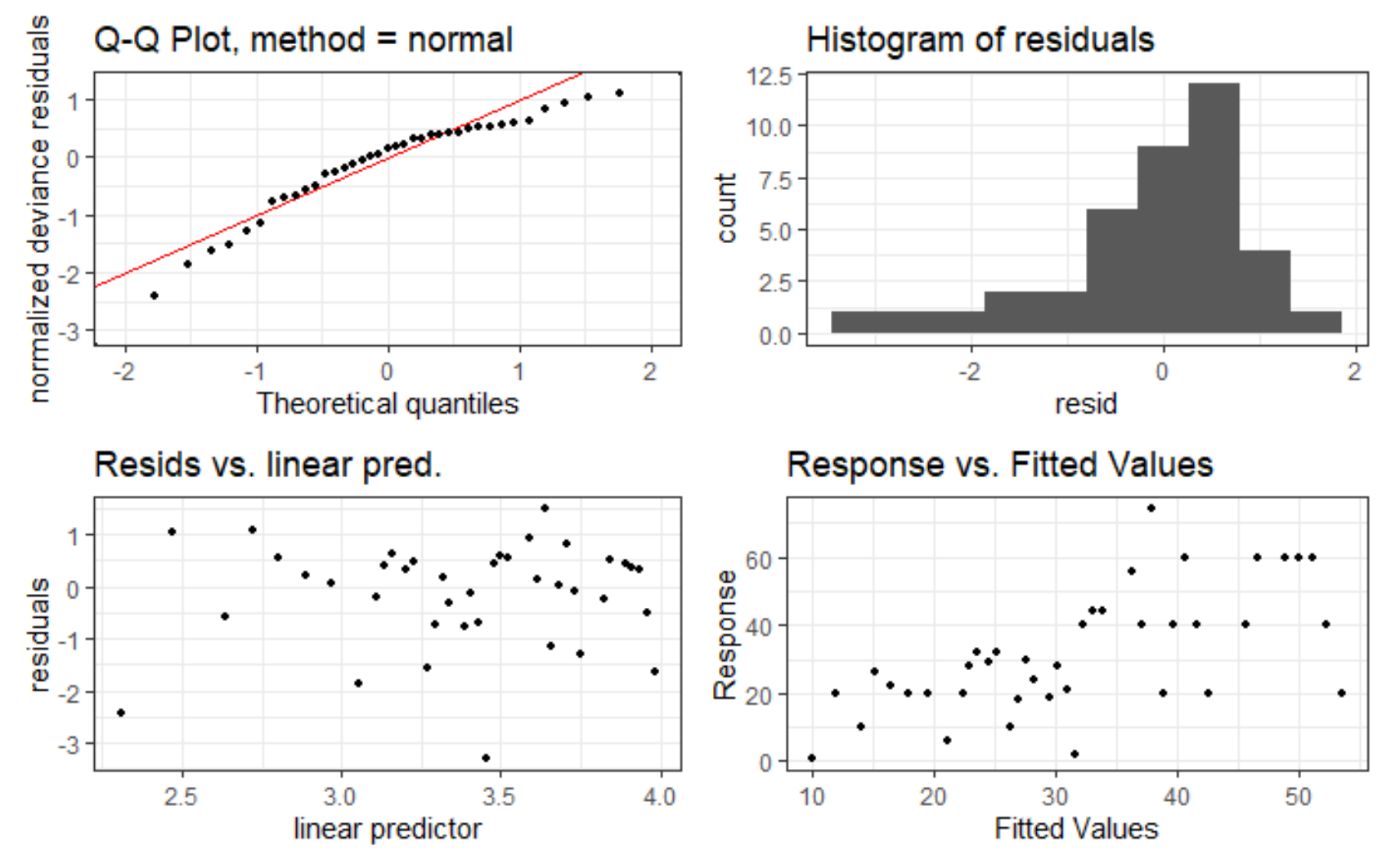 |

## Community perspective

Table S8: Household survey results about health care seeking behavior, comparing reported behavior at the beginning of the COVID-19 pandemic and the month before the survey, CAR

|  | **Proportion of HH who reported an illness event** | | **Proportion of HH who sought care when ill** | | **Where did you seek care** | | | | | | | | **Top four symptoms** | | | | | | | |
| --- | --- | --- | --- | --- | --- | --- | --- | --- | --- | --- | --- | --- | --- | --- | --- | --- | --- | --- | --- | --- |
|  |  |  |  |  | **Hospital** | | **Health Clinic** | | **Pharmacy** | | **Traditional Healer** | | **Fever** | | **Cough** | | **Severe Diarrhea** | | **Chronic Headaches** | |
|  | Early C19 | Sept 2021 | Early C19 | Sept 2021 | Early C19 | Sept 2021 | Early C19 | Sept 2021 | Early C19 | Sept 2021 | Early C19 | Sept 2021 | Early C19 | Sept 2021 | Early C19 | Sept 2021 | Early C19 | Sept 2021 | Early C19 | Sept 2021 |
| **Overall** | 24% | 42% | 61% | 72% | 48% | 66% | 25% | 43% | 16% | 5% | 14% | 7% | 59% | 56% | 23% | 20% | 19% | 15% | 19% | 19% |
| **HoH’s age** |  |  |  |  |  |  |  |  |  |  |  |  |  |  |  |  |  |  |  |  |
| 18-29 | 23% | 36% | 57% | 70% | 45% | 65% | 27% | 48% | 14% | 7% | 14% | 7% | 53% | 67% | 31% | 22% | 24% | 22% | 20% | 21% |
| 30-59 | 24% | 42% | 61% | 70% | 45% | 62% | 26% | 47% | 17% | 4% | 16% | 7% | 62% | 60% | 19% | 20% | 16% | 13% | 19% | 20% |
| 60 + | 26% | 55% | 72% | 85% | 66% | 82% | 13% | 25% | 13% | 4% | 0% | 4% | 61% | 26% | 26% | 15% | 21% | 12% | 13% | 16% |
| **HoH’s sex** |  |  |  |  |  |  |  |  |  |  |  |  |  |  |  |  |  |  |  |  |
| Female | 25% | 47% | 62% | 72% | 55% | 65% | 19% | 45% | 17% | 5% | 12% | 6% | 56% | 54% | 25% | 19% | 19% | 13% | 17% | 21% |
| Male | 22% | 35% | 61% | 72% | 33% | 68% | 35% | 39% | 15% | 5% | 17% | 7% | 65% | 61% | 18% | 21% | 18% | 18% | 22% | 16% |
| **Displacement Status** |  |  |  |  |  |  |  |  |  |  |  |  |  |  |  |  |  |  |  |  |
| Non-displaced | 24% | 42% | 61% | 72% | 48% | 66% | 25% | 43% | 16% | 5% | 14% | 7% | 59% | 56% | 23% | 20% | 19% | 15% | 19% | 19% |
| Displaced | 19% | 41% | 31% | 60% | 25% | 49% | 39% | 40% | 0% | 2% | 11% | 6% | 78% | 68% | 42% | 29% | 28% | 15% | 17% | 23% |
| **Health District** |  |  |  |  |  |  |  |  |  |  |  |  |  |  |  |  |  |  |  |  |
| Bangui | 24% | 44% | 58% | 78% | 50% | 68% | 25% | 41% | 8% | 3% | 17% | 7% | 54% | 60% | 25% | 21% | 17% | 16% | 19% | 20% |
| Begoua | 12% | 24% | 90% | 79% | 75% | 70% | 5% | 10% | 0% | 17% | 20% | 10% | 70% | 74% | 10% | 3% | 5% | 5% | 15% | 13% |
| Bimbo | 25% | 40% | 67% | 56% | 40% | 57% | 25% | 54% | 35% | 9% | 6% | 6% | 73% | 44% | 18% | 16% | 25% | 13% | 17% | 18% |
| **Setting** |  |  |  |  |  |  |  |  |  |  |  |  |  |  |  |  |  |  |  |  |
| Rural | 14% | 29% | 73% | 64% | 44% | 49% | 31% | 27% | 22% | 17% | 9% | 15% | 80% | 60% | 24% | 14% | 27% | 18% | 7% | 12% |
| Urban | 25% | 43% | 61% | 73% | 48% | 67% | 24% | 44% | 16% | 4% | 14% | 6% | 58% | 56% | 23% | 20% | 18% | 14% | 19% | 20% |

Notes:

Early C19 = the first months of the COVID-19 pandemic

HH = Household

HoH = Head of Household

Table S9: Barriers for not seeking care during the month before data collection (Aug-Sept 2021), CAR

|  | **Barriers for not seeking care** | | | | | |
| --- | --- | --- | --- | --- | --- | --- |
|  | **Financial reasons**  **(too expensive)** | **Illness not severe enough** | **HF too far away** | **Do not trust health care providers** | **Did not know how to access care** | **Security reasons (too dangerous)** |
| **Overall** | 87% | 28% | 22% | 14% | 7% | 2% |
| **Age of HoH** |  |  |  |  |  |  |
| 18-29 | 86% | 34% | 28% | 20% | 1% | 0% |
| 30-59 | 89% | 26% | 19% | 13% | 10% | 3% |
| 60 + | 76% | 28% | 28% | 4% | 0% | 4% |
| **Gender of HoH** |  |  |  |  |  |  |
| Female | 94% | 23% | 23% | 18% | 9% | 3% |
| Male | 71% | 41% | 21% | 7% | 2% | 1% |
| **Displacement Status** |  |  |  |  |  |  |
| Non-displaced | 87% | 28% | 22% | 14% | 7% | 2% |
| Displaced | 93% | 24% | 7% | 0% | 0% | 0% |
| **Health District** |  |  |  |  |  |  |
| Bangui | 79% | 41% | 9% | 12% | 3% | 0% |
| Bégoua | 75% | 38% | 38% | 0% | 25% | 0% |
| Bimbo | 98% | 10% | 40% | 18% | 11% | 5% |
| **Setting** |  |  |  |  |  |  |
| Rural | 86% | 17% | 48% | 12% | 21% | 7% |
| Urban | 87% | 29% | 20% | 15% | 5% | 2% |

Table S10: Factors associated with seeking care during the first months of the COVID-19 pandemic and at the time of data collection, CAR

|  | **Seeking care during first months of COVID-19 pandemic (N=204)** | | | **Seeking care during the 30 days preceding the survey**  **(N=394)** | | |
| --- | --- | --- | --- | --- | --- | --- |
|  | **Odds ratio** | **Confidence interval** | **pvalue** | **Odds ratio** | **Confidence interval** | **pvalue** |
| **Age (ref 18-29)** |  |  |  |  |  |  |
| 30-59 | 2.202145 | .720058 - 6.734793 | 0.163 | 1.162821 | .6356066 - 2.127343 | 0.620 |
| 60 + | 5.657212 | .4828803 - 66.27739 | 0.164 | 4.366886 | .8837768 - 21.5775 | 0.070 |
| **Displacement Status**  **(ref residents)** |  |  |  |  |  |  |
| Displaced | .6557956 | .1664672 - 2.5835 | 0.540 | .9147162 | .4041976 - 2.070041 | 0.829 |
| **Sex (ref male)** |  |  |  |  |  |  |
| Female | 1.618599 | .5867976 - 4.464676 | 0.346 | **2.526121** | 1.004675 - 6.351596 | 0.049 |
| **Religion (ref animist)** |  |  |  | Empty |  |  |
| Christian | .5105945 | .0636536 - 4.095712 | 0.520 | .3025765 | .0512554 - 1.786201 | 0.184 |
| Muslim | 4.481124 | .2975842 - 67.47827 | 0.273 | omitted | - | - |
| **Education (ref none)** |  |  |  |  |  |  |
| Primary | 2.438964 | .3103885 - 19.16484 | 0.390 | 1.288688 | .4846115 - 3.426904 | 0.607 |
| Secondary | **9.31578** | 1.051576 - 82.52734 | 0.045 | **4.317774** | 2.204907 - 8.45531 | 0.000 |
| University | 1.20015 | .0403023 - 35.73893 | 0.915 | 5.424787 | .398224 - 73.89888 | 0.201 |
| **Setting (ref urban)** |  |  |  |  |  |  |
| Rural | .9254409 | .3761906 - 2.276615 | 0.864 | 1.579569 | .7170472 - 3.479601 | 0.253 |
| **Profession (ref none)** |  |  |  |  |  |  |
| Trade | .4950351 | .2346631 - 1.044305 | 0.064 | .5212307 | .2617145 - 1.038083 | 0.063 |
| Agriculture | .2766697 | .0616052 - 1.242526 | 0.092 | **.2260162** | .092632 - .5514656 | 0.001 |
| Public official/ employee | 1.153951 | .1469502 - 9.061588 | 0.890 | 1.369648 | .1177563 - 15.93066 | 0.799 |
| Other | .5995615 | .0672222 - 5.34755 | 0.641 | .7972431 | .0468999 - 13.55219 | 0.874 |
| **Health District (ref Bangui)** |  |  |  |  |  |  |
| Bégoua | **41.35403** | 3.431711 - 498.3392 | 0.004 | 4.576156 | .9093531 - 23.02868 | 0.065 |
| Bimbo | **3.975314** | 1.053123 - 15.00597 | 0.042 | .864283 | .2706733 - 2.759729 | 0.803 |
| **Knowledge of COVID-19 (ref not informed)** |  |  |  |  |  |  |
| Partially Informed | .6045692 | .1548969 - 2.359659 | 0.462 | .3400998 | .1055381 - 1.095982 | 0.070 |
| Informed | 1.295162 | .4295941 - 3.90472 | 0.640 | .9876711 | .2635364 - 3.701555 | 0.985 |
| Well Informed | - | - | - | 2.629322 | .1304352 - 53.00204 | 0.524 |

1. The FGDs with vulnerable persons were mixed gender, with the exception of the FGD of pregnant and nursing women. These FGDs used a slightly adapted questionnaire. [↑](#footnote-ref-1)
2. HDX, FACEBOOK – [*Central African Republic: High Resolution Population Density Maps + Demographic Estimates,*](https://data.humdata.org/dataset/highresolutionpopulationdensitymaps-caf) updated in April 2018. [↑](#footnote-ref-2)
